# Recommendations on the Clinical Application and Future Potential of α-Particle Therapy: A Comprehensive Review of the Results from the SECURE Project

**DOI:** 10.3390/ph18101578

**Published:** 2025-10-18

**Authors:** Valentina Di Iorio, Anna Sarnelli, Stefano Boschi, Maddalena Sansovini, Rosa Maria Genovese, Cipriana Stefanescu, Vlad Ghizdovat, Wael Jalloul, Jennifer Young, Jane Sosabowski, Petra Kolenc, Rachel Roberts, Govert de With, Dimitris Visvikis, Renata Mikolajczak

**Affiliations:** 1 IRCCS Istituto Scientifico Romagnolo per lo Studio dei Tumori “Dino Amadori” IRST, Via Piero Maroncelli 40, 47014 Meldola, Italy; anna.sarnelli@irst.emr.it (A.S.); maddalena.sansovini@irst.emr.it (M.S.); rosamaria.genovese@irst.emr.it (R.M.G.); 2 Department of Pharmacy and Biotechnology, University of Bologna, 40126 Bologna, Italy; stefano.boschi@unibo.it; 3 Biophysics and Medical Physics Department, Faculty of Medicine, “Grigore T. Popa” University of Medicine and Pharmacy, 700115 Iasi, Romania; cipriana.stefanescu@umfiasi.ro (C.S.); vlad.ghizdovat@umfiasi.ro (V.G.); wael.jalloul@umfiasi.ro (W.J.); 4 Barts Cancer Institute—A Cancer Research UK Centre of Excellence, Queen Mary University of London, John Vane Science Centre, Charterhouse Square, London EC1M 6BQ, UK; jennifer.young@qmul.ac.uk (J.Y.); j.k.sosabowski@qmul.ac.uk (J.S.); 5 Division of Nuclear Medicine, University Medical Centre Ljubljana, Zaloska 7, 1000 Ljubljana, Slovenia; petra.kolenc@kclj.si; 6 United Kingdom National Nuclear Laboratory, Building A709, Springfields PR4 0XJ, UK; rachel.fc.roberts@uknnl.com; 7 NRG PALLAS, Utrechtseweg 310, 6800 ES Arnhem, The Netherlands; g.dewith@nrg.eu; 8 INSERM, UMR 1101, University of Brest, 29200 Brest, France; dimitris@univ-brest.fr; 9 Radioisotope Centre POLATOM, National Centre for Nuclear Research, Andrzej Soltan 7, 05-400 Otwock, Poland; renata.mikolajczak@polatom.pl

**Keywords:** TAT, α-emitters, actinium-225, bismuth-213, astatine-211, lead-212, terbium-149, radium-223, thorium-227

## Abstract

This review comprehensively assesses the clinical applications and future potential of alpha-emitting radionuclides available for targeted alpha-particle therapy (TAT) in cancer treatment. The approval of radium-223 therapy in 2013 marked a significant advancement in alpha-emitting therapeutic radiopharmaceuticals, which are primarily used in treatment of prostate cancer. The EU SECURE project was introduced as a major initiative to enhance the sustainability and safety of medical alpha-emitting radionuclides production in Europe. This literature review was conducted by a multidisciplinary team on selected radionuclides, including actinium-225, bismuth-213, astatine-211, lead-212, terbium-149, radium-223 and thorium-227. These were selected based on their clinical significance, as identified in the EU PRISMAP project and subsequent literature searches. The review process involved searching major databases using specific keywords related to alpha-emitter therapy and was limited to articles in English. For each selected radionuclide, the physical characteristics, the radiochemistry, and the pre-clinical and clinical studies are explored. Actinium-225 is the most widely studied alpha emitter, with several preclinical and clinical studies on prostate cancer and neuroendocrine tumours. Other types of tumours (such as glioblastoma) still require preclinical and clinical development. Bismuth-213 bound to antibodies, peptides and nanobodies has shown optimal results in preclinical and clinical studies, with increased median survival and no significant toxicity. Astatine-211 differs from most other α-emitters relevant to TAT, since it yields one α-particle per decay. This offers certain translational advantages, including the simplification of radiation dosimetry calculations and quality control (QC). Lead-212 has the advantage of being an in situ generator with likely widespread availability. Although clinical data are limited, the findings are promising at this stage. The unconventional production of Terbium-149 is the primary reason it has not yet progressed to clinical trials. Overcoming this production obstacle would allow more detailed preclinical investigations. Optimal results with Thorium-227-labelled agents have been observed in preclinical studies, including delays in cellular growth, multiple double-strand breaks and complete regression. Intermediate phase I trial results have also been reported, demonstrating safety and tolerability, as well as an objective response rate of 25%.: The results highlight the advantages of alpha particles in targeting cancer cells with minimal radiation to normal tissue, emphasising the need for high specificity and stability in delivery mechanisms, as well as suggesting that the full clinical potential of alpha particle therapy remains unexplored. Theranostic approach and dosimetric evaluations still represent relevant challenges.

## 1. Introduction

Recent advances in Targeted Radionuclide Therapy (TRT) have expanded cancer treatment options [1]. Whilst Targeted Beta Therapy (TBT) is more advanced in both clinical and commercialised stages, the short range and high linear energy transfer (LET) of alpha (α) particles make them a clear candidate for treating metastatic cancers, single cell tumours, small clusters or non-solid tumours through Targeted Alpha Therapy (TAT) [2,3].

The high LET characteristic results in a high Relative Biological Effectiveness (RBE), with the RBE of α emitters being five times greater than that of beta (β^−^) particles [4]; they are seen to cause irreparable double DNA strand breaks in tumours, whilst minimising the levels of unwanted radiation to surrounding healthy tissue [5].

One of the key considerations in the use of molecules labelled with α emitters is the potential for bond cleavage of the molecule resulting from kinetic energy post-α-decay, termed the recoil effect [6,7,8]. The recoil energy from the α-decay process generally exceeds 100 keV [8] significantly higher than any chemical bond’s binding energy. As a result, the bond between the α-emitter and the chelating agent, carrier system, or entire bioconjugate is likely to break, releasing radioactive daughters. The coordination characteristics of a radionuclide can differ significantly from those of its decay products, which may lead to chemical instability in the metal–chelator complexes. Additionally, for radionuclides with complex decay chains such as actinium-225 and thorium-227, the variations in the coordination chemistry between parent and daughter isotopes become critical [9]. This is because a single chelating agent is generally insufficient to bind all the daughter effective isotopes throughout the decay chain. The recoil effect and changes in coordination are crucial when daughter radionuclides are α-emitters, as they may accumulate in off-target areas and deposit cytotoxic energy at undesired locations if they reach their biological target.

Despite the wide range of alpha-emitting radionuclides in existence, only a small selection are appropriate for use in TAT with criteria for suitability including, amongst others, high specificity and stability in the delivery mechanisms, a suitable route to production without considerable radionuclidic impurities, a sufficiently long half-life to reach the tumour for treatment (>1 h) and a sufficiently short half-life so as not to persist within the body following treatment (<1 month) [2]. The final characteristic can introduce complexity for production and distribution methods of these essential radionuclides, sometimes requiring the production of a longer-lived parent radionuclide from which the relevant α emitter can be separated.

The EU SECURE (Strengthening the European Chain of sUpply for next generation medical RadionuclidEs) project was established to contribute to the sustainability of medical isotope production and its safe application in Europe. The ambition of the SECURE consortium is to identify and efficiently use the current resources for new radionuclides, for alpha emitters, which would create new opportunities for society, healthcare and economics. Among various tasks, the SECURE contributed to recommendations for clinical trials and radiation protection (the reference can be made here to the recent publication [10]. In the current paper, the background information is collected and summarised, which served to achieve these goals (or may be useful for future applications).

Some of the project’s core objectives were to strengthen production routes and to develop a set of recommendations for clinical application of alpha emitters relevant to TAT. To achieve the latter, a list of TAT relevant radionuclides were selected as Actinium-225, Bismuth-213, Astatine-211, Lead-212, Terbium-149, Radium-223, and Thorium-227 (with Lead-212 being the β^−^ emitting in vivo generator to Bi-212) for review, covering their physical characteristics, known production methods, radiochemistry and delivery mechanisms, biodistribution of decay chain daughters, and reported preclinical and clinical studies.

A comparative table of key differences between alpha (α) vs. beta (β) emitters and physical characteristics of the α-radionuclide is shown Table S1 (Supplemental Data).

Where possible, scenarios are considered within the context of different clinical cancer models, including glioblastoma, neuroendocrine tumours, bone metastases, multiple myeloma, and associated standard state-of-the-art treatment techniques.

The EU SECURE project did not include Radium-224, an alpha emitter authorised by the FDA as a medical device. It consists of billions of calcium carbonate microparticles containing Radium-224 for locoregional treatments.

## 2. Results

### 2.1. Actinium-225

#### 2.1.1. Physical Characteristics

Actinium is a radioactive component with atomic number 89 [11]. Only two of its 32 isotopes, Actinium-228 and Actinium-227 are naturally produced by the disintegration of Thorium-232 and Uranium-235, respectively [11,12]. With its long half-life of 21.7 years and predominant β-emission decay, Actinium-227 represents the most common actinium isotope. However, Actinium-228, a β-emitter, is highly uncommon [11,12].

Actinium-225 is the initial element in the actinide family, and its radioactive parents are parts of the now-extinct “neptunium series” [11,13]. This alpha emitter isotope has a long half-life of 9.9 days [14,15].

**Figure 1:**
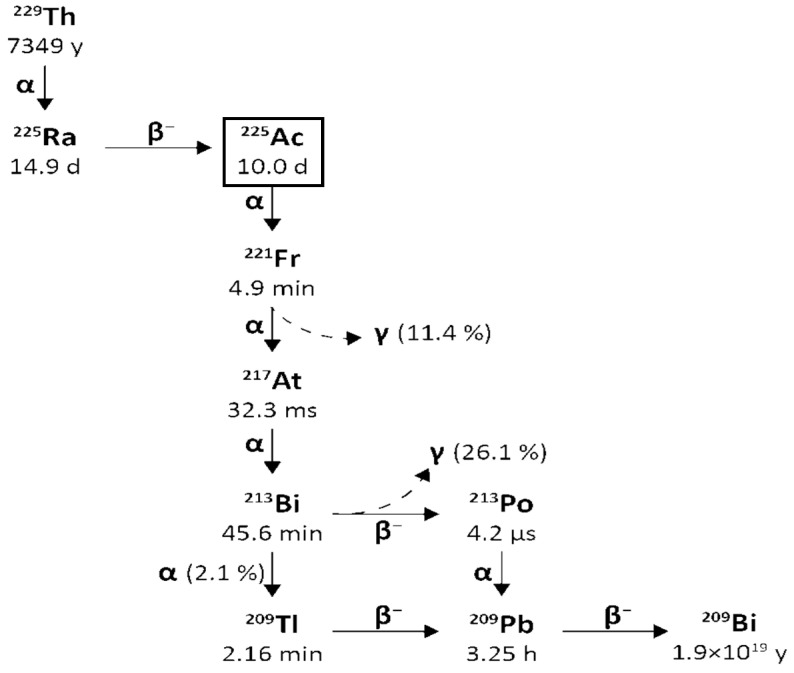
Decay chain of Uranium-233 to Actinium-225 and Bismuth-213.

From Actinium-225 to Bismuth-209 (T1/2 = 1.9 × 10^19^ y), the decay series includes six short-lived radionuclide daughters [14,16]. This radioactive cascade is represented by Francium-221 (T1/2 = 4.8 min; 6.3 MeV α particle and 218 keV γ emission), Astatine-217 (At-217) (T1/2 = 32.3 ms; 7.1 MeV α particle), Bismuth-213 (T1/2 = 45.6 min; 5.9 MeV α particle, 492 keV β^−^ particle and 440 keV γ emission), Polonium-213 (T1/2 = 3.72 µs; 8.4 MeV α particle), thallium-209 (T1/2 = 2.2 min; 178 keV β^−^ particle), Lead-209 (T1/2 = 3.23 h; 198 keV β^−^ particle) and stable Bismuth-209 (Figure 1) [17,18].

Actinium-225 is considered a “nanogenerator” since one decay of this element produces four α, three β particles, and two γ emissions [17]. The α particle emissions and the rapid disintegration of Actinium-225 make it an appealing choice for targeted radionuclide therapy (TAT) [17,19]. However, it is essential to consider the notable Actinium-225 cytotoxicity due to its extended half-life and the various α particles produced throughout its decay chain [14].

Moreover, the potential use of γ disintegrations, produced by the decay of the intermediate francium-221 (218 keV, 11.6% emission probability) and bismuth-213 (440 keV, 26.1% emission probability) [14] in SPECT in vivo imaging, could lead the Actinium-225 radioactive cascade to a possible theragnostic perspective and nuclear medicine applications.

However, the recoil effect remains a challenge that must be carefully managed to maximise its potential. Innovations in encapsulation, intracellular delivery, and chelation chemistry are helping to mitigate recoil and enhance the safety and effectiveness of Actinium-225 in clinical applications.

Planar SPECT imaging faces challenges due to Actinium-225s effectiveness, resulting in low doses and γ emissions. Using Bismuth-213 from Actinium-225 decay is a potential solution, but Bismuth-213’s short half-life (45.6 min) complicates processing, radiolabelling, and radiopharmaceutical delivery. Monitoring these reactions is also challenging due to the necessary radiation and the requirement of a 6 h secular equilibrium for accurate radiochemical yield measurement. Actinium’s chemistry remains underdeveloped due to limited availability and the specific management needed for all Ac isotopes.

As previously mentioned, Actinium-225 is part of the Neptunium-237 decay series, which is no longer found naturally. This radioactive element can be artificially synthesised [11]. In addition to direct production methods, Actinium-225 can be accessed at several key points along its decay chain, including Uranium-233 (half-life = 159,200 years, 100% alpha emission), Thorium-229 (half-life = 7340 years, 100% alpha emission), and Radium-225 (half-life = 14.9 days, 100% beta-minus emission) [13].

Actinium-225 has significantly fewer nucleons than other actinide nuclei, making it less stable than production targets such as thorium-232 and radium-226 [13]. Consequently, production methods typically rely on radioactive decay or high-energy bombardments, with few exceptions.

**Figure 2:**
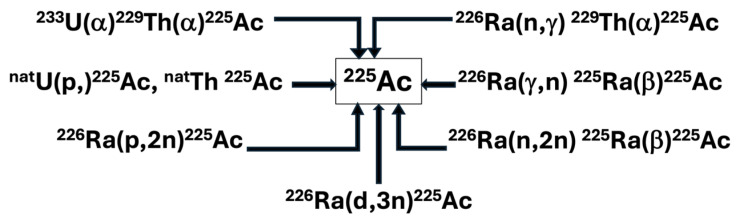
Principal production routes for Actinium-225.

The available production routes of Actinium-225 and its parents are listed below in Figure 2.

##### Radiochemical Extraction from Thorium-229

For over two decades, the primary source of Actinium-225 has been the accumulation of thorium-229 (T1/2 = 7340 y) from the disintegration of uranium-233 (T1/2 = 160,000 y) reserves. All clinical trials and many preclinical studies involving Actinium-225 and bismuth-213 have used this generation route [14].

A large portion of uranium-233 was created between 1954 and 1970 by neutron irradiating thorium-232 while it was under research for nuclear weapons and reactors never wholly implemented [20,21]. Nuclear plants have a significant stockpile of uranium-233 after the thorium fuel cycle was abandoned in favour of fast reactors powered by plutonium at the end of the 1970s [11]. From supplies kept at the Oak Ridge National Laboratory (ORNL, Oak Ridge, TN, USA), thorium-229 produced via uranium-233 disintegrations was recovered between 1995 and 2005 [21]. Currently, there are three principal sources for this Thorium-229: at ORNL (5.55 GBq (150 mCi), or 704 mg) [21,22], at the Directorate for Nuclear Safety and Security of the Joint Research Centre (JRC) of the European Commission (JRC, Karlsruhe, Germany) (1.7 (46 mCi), or 215 mg) formerly known as the Institute for Transuranium Elements (ITU) [22,23], and at the Leipunskii Institute for Physics and Power Engineering (IPPE, Obninsk, Russia) (5.55 GBq (150 mCi), 704 mg) [21,24]. The Canadian Nuclear Laboratories has more recently announced the isolation of a crucial Thorium-229 source [14]. Very pure sources of Thorium-229 were also discovered, prepared, and used for preclinical research at the Belgian Nuclear Research Centre (SCK CEN) in Mol, Belgium [18].

By producing approximately 33 GBq (893,23 mCi) (ORNL) [17] and 13.1 GBq (350 mCi) (JRC) [12,21] of Actinium-225 annually, ORNL and JRC represent, up to now, the principal worldwide providers of Actinium-225 and its parent Radium-225 (T1/2 = 14.9 d). Anion exchange and extraction chromatography are combined to produce Actinium-225 from Thorium-229 at JRC Karlsruhe, whereas anion and cation exchange are used at ORNL [25]. Even though the IPPE source has the same amount of Thorium-229 as the ORNL source, recorded values show that this source intermittently produces Actinium-225 [21,24,26]. According to [27], IPPE Actinium-225 production could reach 22 GBq per year.

Additionally, it has been noted that, beginning in 2019, the extraction of Thorium-229 from historical waste stored by the US Department of Energy is expected to considerably increase the availability of Thorium-229 [25]. According to estimations, up to 45 g of the total Thorium-229 could be available, which could result in a 40-fold boost in the supply of Actinium-225 above current levels [25].

Globally, approximately 68 GBq of actinium-225 is generated annually from thorium-229 [14]. Knowing that the Actinium-225-labelled ligands given activities typically range from 4 to 50 MBq per therapeutic dosage [14], this isotope’s supply is sufficient to treat several hundred patients annually and permits the performance of preclinical research. Although a significant benefit of this production method is that the resulting Actinium-225 is free of other actinium isotopes, the globally generated Thorium-229 is not enough to satisfy the extensive use and implementation in healthcare applications worldwide [21]. Therefore, the development of Actinium-225 radiopharmaceuticals is hindered by the limited supply and high cost, that make Actinium-225 inaccessible to many researchers [21]. In addition, the production of uranium-233 (T1/2 = 160,000 y) is not viewed as a realistic solution for addressing expected short-term Actinium-225 demand because decades of steady growth are necessary to boost Thorium-229 (T1/2 = 7340 y) supply, consequently [13,28,29]. As a result, numerous other techniques for generating Actinium-225 on a wide scale have been researched.

Exposing radium targets to high fluxes of thermal neutrons is considered an effective procedure to induce Thorium-229 production [13]. ORNL researchers have carefully investigated this approach with access to the High Flux Isotope Reactor’s (HRIF) >1015 n cm-2 s-1 thermal flux, noticing the production of Thorium-229 from Radium-226, Radium-228, and Actinium-227 [15]. A HFIR cycle of 26 day generated thorium-229 yields at 74 ± 7.4 MBq/g from Ra-226, 260 ± 10 Bq/g Thorium-229 from Ra-228, and 1200 ± 50 MBq/g from Actinium-227 [27]. The predominant generation pathway from Radium-226 targets, ^226^Ra(n,γ), ^227^Ra(β^−^)^227^Ac (n,γ), ^228^Ac (β^−^), ^228^Th (n,γ) ^229^Th, is driven by a combination of neutron capture probability and decay kinetics [13]. The short half-lives of Radium-227 (T1/2 = 42.2 min, 100% β^−^) and Actinium-238 (T1/2 = 6.15 h, 100% β^−^) represented the crucial restrictions for these possible Thorium-229 generation routes [13]. The magnitude of the ^226^Ra(n, γ) ^229^Th cross-section has the most significant impact on the amount of Thorium-229 that can be produced [13]. Unfortunately, this predominant pathway passes through Th-228. This thorium radionuclide is a dosimetrically undesirable contaminant that can only be eliminated from Thorium-229 by mass isolation or burnup and lowers the yield of Thorium-229 that may be produced [13]. Handling the radium target and generating Thorium-228 (T1/2 = 1.9 y) represent essential challenges of this process [18,30]. In addition, there is still a sizable gap between theoretically predicted and measured yields. In HFIR, ideal 5-cycle activations are expected to provide approximately 0.8 GBq (20 mCi g-1) of Thorium-229 for every gram of Radium-226 [13].

Although pure Actinium-227 or Radium-228 targets are projected to generate somewhat more Thorium-229, the current supply of these radionuclides is substantially less than that of Radium-226 [13].

##### Accelerator-Based Routes

The spallation of thorium-232

This method is based on the spallation of Thorium-232 to produce Actinium-225. As a target material, Thorium-232 (4.1103 Bq/g, 110 nCi/g) is widely accessible, not excessively radioactive, and presents fewer radiation risks [21,31]. Due to its accessibility, recycling Thorium-232 target material may not be an issue.

The irradiation of Thorium-232 with highly energetic protons (0.6–2 GeV) accessible at large accelerators has produced considerable amounts of Actinium-225 [14,32,33]. Production yields of several GBq have been recorded for 10 days’ irradiations utilising highly energetic proton beams [15,34,35]. From the irradiations of 5 g cm^−2^ targets throughout their roughly 8-month annual running durations, Los Alamos National Laboratory can create between 40 and 80 GBq (1–2 Ci) every 10 days. Once the targets are handled and the completed product is delivered from ORNL, irradiations can be carried out at Brookhaven National Laboratory (200 MeV at 165 mA) and Los Alamos National Laboratory (100 MeV at 275 mA) [25].

The co-production of long-lived Actinium-227 (T1/2 = 21.8 years) is the process’s primary constraint [14,36,37,38].

The effects of the isotopic impurity on the therapeutic application of the produced Actinium-225 need to be considered because Actinium-225 and Actinium-227 cannot be chemically separated (0.1–0.2% of the relative activity of Actinium-225) [33]. Even with this limitation, Actinium-225 produced from high-energy accelerators may still be perfectly suitable for manufacturing Actinium-225/Bismuth-213 generators, as all actinium daughters will be kept on the generator [18].

According to preliminary research, the Actinium-227 impurity will not significantly affect patient dosimetry [28]. Recently, new purifying techniques have been developed that enable a reduction in the Actinium-227 level and the recovery of Actinium-225 with better purity, such as isotope separation (Isotope Separation On-Line, ISOL at TRIUMF) or a manufacturing method using radium-225 produced after proton irradiation of thorium-232 [39].

Nonetheless, challenges remain regarding long-lived Actinium-227 licensing and accessibility in medical applications. In addition, with a half-life of 21.8 years, waste management is still a serious issue that will necessitate measures with possibly high related costs.

Proton irradiation of radium-226

Compared to the Thorium-232 spallation reaction, the generation of Actinium-225 from Ra-226 targets by proton irradiation in a cyclotron in the ^226^Ra(p,2n)^225^Ac nuclear reaction has several benefits. In medium-sized cyclotrons, at proton energies below 20 MeV (around 16 MeV), this procedure can be carried out 500 patient doses of 10 MBq Actinium-225, should be produced after 24 h exposure to 50 mg Radium-226 to the highest excitation function at 15–16 MeV with a current of 100 mA protons [18,25,40].

Since no other long-lived actinium isotopes, such as Actinium-227, are created, Actinium-225 with high isotopic purity is obtained. By choosing the correct proton energies, it is possible to reduce the co-production of the short-lived actinium-226 (T1/2 = 29 h) and Ac-224 (T1/2 = 2.9 h) impurities produced by the reactions ^226^Ra(p,n) ^226^Ac and ^226^Ra (p,3n)^224^Ac [14,25].

Furthermore, during the time needed for target cooling and reprocessing, their activity will continue to decrease to low levels. The production, processing, and control of targets containing milligram quantities of radioactive Radium-226 (T1/2 = 1600 years), as well as the control of its highly radiotoxic gaseous decay product radon-222 (T1/2 = 3.8 days) [14,18,41] pose the procedure’s principal difficulties [14,25]. In addition, due to the limited availability of the target material, it is necessary to consider its recycling process [12].

Deuterons irradiation of radium-226

Producing Actinium-225 has been proposed by irradiating Radium-226 with deuterons through the reaction ^226^Ra(d,3n) ^225^Ac [42]. Although experimental measurements of the reaction’s cross-sections are still in development, simulations indicate that the process will have a bit greater production yield than the ^226^Ra(p,2n) ^226^Ac reaction and a maximum cross-section of 864 mb at 18.5 MeV [25].

Since deuteron irradiation might result in an increased co-production of actinium-226 (T1/2 = 29 h), a prolonged cooling time should be considered to allow the actinium-226 decay [25]. This factor is a critical consideration in the production process and must be carefully managed to ensure the quality of the final product.

Photonuclear irradiation of radium-226

The photonuclear reaction ^226^Ra (γ,n) ^225^Ra, followed by the beta decay of Radium-225 to Actinium-225, is a different method for producing Actinium-225 by irradiating Radium-226. It was noticed that the photon energy cut-off for the reaction was 6.4 MeV. However, experimentally established cross-section data are not yet available [25]. A zircaloy capsule containing 1 mg of Radium-226 embedded in an 800 mg of BaCl_2_ matrix underwent 3.5 h of 52 MeV betatron irradiation to generate 0.24 mCi of Actinium-225 [25]. At a maximum photon energy of 24 MeV, a radiation yield of 550 Bq/(mAh mg radium-226) was recorded [43]. For a more precise estimate of production yields, it is essential to quantify the cross-section data in detail for this reaction.

The Ra-226 target recycling requirement and issues with the radon-222 emission represent the principal difficulties regarding this approach [12]. However, large-scale Actinium-225 manufacturing using this procedure is already being implemented at several plants [44].

SCK-CEN and IBA signed a research and development partnership agreement named Pantera for the joint production of Actinium-225 in 2021. Thorium-229 naturally decays to Ra-225 and then Actinium-225, which allows for extracting significant amounts of Actinium-225. This generator is installed in a Pantera production facility hosted on the premises of SCK CEN (EU H2020 project PANTERA -PAN European Technology Energy Research Approach).

#### 2.1.2. Radiochemistry

Actinium commonly forms +3 ions in water, resembling lanthanum’s +3 chemistry. La^3+^ is often used as a nonradioactive surrogate for Ac^3+^. The 6-coordinate ionic radius of La^3+^ (1.03 Å) is smaller than Ac^3+^ (1.12 Å) [45]. The low charge density renders Ac^3+^ a very basic +3 ion. The first hydrolysis constant, pK 1 h, represents the ability of the metal to polarise coordinated water to favour the release of a proton and the formation of Ac(OH)^2+^. For Ac^3+^, this was measured by an ion exchange method and determined to be 9.4 ± 0.1 [46].

This study also measured the pK1h of La(III) as 9.0 + 0.1 under similar conditions. Other studies show the first hydrolysis constant of La^3+^ to be 8.63 by different methods [47]. These studies suggest that the first hydrolysis constants are consistent with the charge densities of Ac^3+^ and La^3+^, indicating that Ac^3+^ is a “hard” metal ion. This information suggests the use of basic conditions for radiolabelling of Ac-complexes.

Spectroscopically, Ac^3+^ is invisible to many forms of routine spectroscopy, such as ultraviolet–visible, fluorescence, electron paramagnetic resonance, etc., due to its electronic configuration (5f 0 6d 0). Ferrier et al., using the long-lived isotope Actinium-227 (t1/2: 21.772 y), measured the L3-edge X-ray absorption near-edge structure (XANES), representing the first actinium XANES measurement. This study bodes well for the study of actinium via X-ray absorption spectroscopy (XAS) [48].

The interpretation of the extended X-ray absorption fine-structure (EXAFS) data from room-temperature solutions containing Ac in HCl demonstrated that the Ac^3+^ was coordinated to ∼3 Cl^−^ and ∼6 H_2_O inner-sphere ligands. The calculated coordination numbers agreed with experimental values. This study showed that Actinium tends to possess more Cl^−^ inner sphere ligands than Americium, which is consistent with the notion that Ac^3+^ is substantially less polarising than the rest of the f-elements and confirms it as a hard acid. Later, the group reported an XAFS study wherein 10.9 + 0.5 water molecules were directly coordinated to the Ac^3+^ cation with an Ac-OH_2_O distance of 2.63 (1)Å [48]. This agreed with the Molecular Dynamics Density Functional Theory (MD-DFT) results.

Having 11 inner sphere water molecules is reasonable for the large Ac^3+^ ion; this is consistent with the coordination numbers determined by EXAFS for other +3 actinide and lanthanide aqua ions. The coordination number of 11 is also consistent with the current ligands and stable bismuth-209 [49].

Since Actinium-225 itself cannot be detected directly with gamma spectroscopy, as it does not emit a detectable gamma ray, time must be allowed for the detectable daughter, Bismuth-213, to grow and be observed by gamma detection.

##### Chelating Agents for Actinium-225

The discovery of a chelating agent that binds Ac(III) with sufficient stability and controls the release of its daughter nuclides remains a challenge. Moreover, the limited global availability of Actinium-225 and the absence of a stable surrogate nuclide have limited the study of this isotope to a handful of institutions worldwide that have secured a reliable Actinium-225 supply. Most initial Actinium-225 chelation studies have focused on screening a variety of commercially available polydentate macrocyclic or acyclic ligands for their ability to bind Actinium-225 and form stable complexes in vitro or in vivo.

Despite the unique coordination preferences of the large +3 actinide, the literature reports very few studies investigating new ligands specifically designed to coordinate Ac(III).

A brief outline of chelators tested with Actinium-225 is shown in Table S2 (Supplemental Data).

##### Actinium-225-Labelled Nanoparticles

Researchers have sought to encapsulate the highly potent alpha-emitter into a nanoparticle structure to circumvent the inevitable loss of Actinium-225 daughters after alpha decay from an actinium–chelate complex. It is hypothesised that the [^225^Ac]Ac^3+^ ion and its decay daughters can be retained within the cavity of the nanoparticle structure. At the same time, the alpha particles are released and able to deposit their therapeutic dose at the intended target site.

However, using nanoparticles as a platform to affix radionuclides or other biomolecular targeting vectors comes with several limitations. The biodistribution of nanoparticles depends on their large size and ability to take advantage of the enhanced permeability and retention (EPR) effect of cancer cells, where ‘leaky’ vessels of poorly vascularized tumours allow for the uptake and retention of large macromolecules. Moreover, the relatively large particles are often primarily excreted through the hepatic pathway, which can cause unwanted high liver uptake. These challenges underscore the complexity of using nanoparticles in Actinium-225 research and highlight the need for further investigation.

The accumulation of a highly toxic alpha-emitter in the liver may damage the organ. Much of the available literature describing Actinium-225-labelled nanoparticles provides only in vitro data [50]. Below is a brief overview of some strategies for preparing Actinium-225-radiolabelled nanoparticles.

The encapsulation of [^225^Ac]Ac^3+ +^ ions in single-walled carbon nanotubes (SWNTs) by co-encapsulation of Gd3+ in an ion cluster was investigated. Although the Gd^3+^ ions remained inside the SWNTs, continual leakage of the [^225^Ac]Ac^3+^ ions was seen when challenged with serum [51].

Some authors employed a multi-layered nanoparticle structure that can contain the recoiling daughters of the in vivo alpha generator at the centre cavity while coupling the outer layer to antibodies without preventing the release of emitted alpha particles. The shells included a radiation-resistant lanthanide phosphate crystal doped with Actinium-225 and layered with a magnetic GdPO_4_ layer, plus a gold outer shell to attach targeting vectors [7].

Polymer vesicles (polymersomes) composed of poly(butadiene-b-ethylene oxide) have also been used to encapsulate Actinium-225 [7]. Preliminary in vitro studies in cells showed that smaller particles were absorbed by the cells and gathered around the cell nucleus. However, experiments and simulations indicated that larger polymerases are needed to retain recoiling daughters [7] correctly.

PEGylated liposomes loaded with Actinium-225 and labelled with mouse anti-human PSMA J951 antibody or with the A10 PSMA aptamer were tested in vitro for their targeting, internalisation, and cytotoxicity on a prostate cancer cell line [52]. These studies demonstrated that anti-PSMA targeted liposomes loaded with Actinium-225 can selectively bind, become internalised, and kill PSMA-expressing cells.

Similarly, Actinium-225-loaded lipid-based nanocarrier was labelled with a PSMA-targeting antibody or small-molecule urea-based agent, and the targeting selectivity and cytotoxicity were compared to those of the radiolabelled antibody on its own [50]. It was found that the loaded lipid vesicles improved the killing efficacy threefold compared to the same levels of activity per cell when delivered by the PSMA-targeting antibody.

##### Assessing the Biodistribution of the Actinium-225 Decay Chain

When evaluating the performance of Actinium-225 radiopharmaceuticals, the biodistribution of each alpha emission in the decay chain must be assessed. The retention or redistribution of francium-221, astatine-217, and Bismuth-213 at the target site impacts the radiopharmaceutical’s efficacy and toxicity.

While the half-life of astatine-217 is short enough that its biodistribution can be assumed to be effectively identical to francium-221, the short half-life of francium-221 makes accurately determining its biodistribution—and also independently determining the biodistribution of its bismuth-213 granddaughter—a challenge using conventional ex vivo counting methods

Speedy harvesting and counting of organs are essential. While successive measurements of the same ex vivo tissue samples over time can be used to estimate the amount of francium-221 or Bismuth-213 present at the time of sacrifice, the uncertainty in these estimates increases the longer after sacrifice the first measurements are made [1].

Imaging-based methods can also help assess the biodistribution of the radionuclides in vivo, and quantitative SPECT imaging of Actinium-225 progeny isotopes has been demonstrated on small-animal SPECT/CT systems for Bismuth-213 alone [53] and both francium-221 and Bismuth-213 simultaneously, via their 218 keV and 440 keV gamma lines, respectively [54]. Unfortunately, quantitative imaging of the high-energy Bismuth-213 photopeak (440 keV) requires a high-energy collimator unavailable on most imaging systems. However, qualitative SPECT imaging of Bismuth-213 has been performed clinically, as has qualitative francium-221 SPECT in preclinical settings [55,56,57,58,59].

Cerenkov imaging has also been demonstrated in vivo for the Actinium-225 decay chain [60]. However, this imaging modality is incapable of quantitative biodistribution measurements and cannot distinguish between individual Actinium-225 decay chain components.

While quantitative SPECT imaging of francium-221 and bismuth-213 with the sub-millimetre spatial resolution has the potential to assess the retention of Actinium-225 progeny within the tumour and determine uptake within whole organs [54], the short range of alpha particles means that information regarding the sub-organ biodistribution—a level of detail not provided by current in vivo imaging modalities—is necessary for alpha-particle dosimetry [61,62].

While ex vivo imaging using alpha-cameras can determine Actinium-225 biodistributions with spatial resolutions sufficient for dosimetry [63,64,65], alpha particle dosimetry itself faces additional challenges that currently limit the translation of preclinical dosimetry data to biological outcomes in the clinic [61,62].

#### 2.1.3. Preclinical Studies

Preclinical studies of Actinium-225 demonstrate its potential as a powerful alpha-emitter for targeted alpha therapy (TAT), showing significant anti-tumour efficacy in animal models by accumulating in and killing cancer cells. Studies focus on developing stable chelators (MACROPA) to prevent premature release of the radioactive isotope and evaluating the biodistribution of [^225^Ac]Ac-conjugates to identify suitable targeting vectors like peptides, antibodies, or small molecules for specific cancers such as neuroendocrine tumours (NETs) and HER2-positive cancers. List of relevant preclinical studies involving Actinium-225 is shown in Table S3 (Supplemental Data).

#### 2.1.4. Clinical Studies

Currently, actinium-225 is the radionuclide with the most clinical trials on ClinicalTrials.gov and is the only one with an active phase III trial (NCT05477576). While most clinical trials are sponsored by pharmaceutical companies, there are also some academic studies, primarily conducted in the USA. Interestingly, two of the trials are sponsored by the National Cancer Institute (NCT06888323 and NCT06802523).

Many studies involve the use of [^225^Ac]Ac-conjugates in neuroendocrine tumours and prostate cancer following failure of previous therapies with beta-emitting conjugates, such as Lu-177. The most widely researched radiopharmaceutical in haematology is [^225^Ac]Ac-lintuzumab, which is also used in combination with other haematological drugs. In this context, two phase I clinical trials are also underway with [^225^Ac]Ac-DOTA-daratumumab (NCT06287944 and NCT05363111).

List of clinical studies involving Actinium-225 reported in the database ClinicalTrials.gov is shown in Table S4 (Supplemental Data).

#### 2.1.5. Conclusions

The emission of multiple alpha-particles in the Actinium-225 decay chain (Figure 1) makes Actinium-225 a particularly effective isotope to kill cancer cells and also challenges the directed delivery of the nuclide and its decay daughters. Due to the conservation of momentum, the emission of an energetic alpha particle (energies shown in Figure 1) imparts a recoil energy to the daughter nucleus, often >100 keV, 1000 times larger than the binding energy for any chemical bond [1]. The subsequent redistribution of the alpha-emitting daughter nuclides in vivo can cause substantial harm to untargeted healthy tissues and reduce the therapeutic effect. Renal toxicity caused by Bismuth-213 limits the use of Actinium-225 in many clinical trials [66]. There are three main strategies for limiting the toxicity of recoil daughters in the literature: fast uptake and internalisation of the alpha emitters in the target tissue, encapsulation of the nuclide in a nanoparticle, or local administration of radioactivity directly into the target site via injection [1]. The studies published until now demonstrate that, even though there was a greater concentration on prostate cancer and NET preclinical and clinical studies, some other tumoral types (like glioblastoma) still need preclinical and clinical development. The studies justify several pros and cons regarding the role of Actinium-225 in TAT. Some observations, like the local administration, could be related to further insights into Actinium-225-radiolabelled vector molecules studies in TAT.

### 2.2. Bismuth-213

#### 2.2.1. Physical Characteristics

Bismuth belongs to Periodic Group 15 and has 35 isotopes. Most have short half-lives (from nanoseconds to a few minutes), and just one is deemed stable, bismuth-209, due to its extremely long half-life of 1.9∙1019 years. From a clinical point of view, only bismuth-212 and bismuth-213 have shown potential properties for research in targeted radionuclide treatment [11].

Bismuth-213 is one of the decay products of Actinium-225 and has a physical half-life of 45.6 min. It decays into polonium-213 via β^−^ emission (Eβ = 1.4 MeV, 97.84%) and into thallium-209 via α emission (Eα = 5.549 MeV, 0.16%, Eα = 5.869 MeV, 2.0%). polonium-213 and thallium-209 later undergo α and β decay, respectively (Eα = 8.375 MeV, Eβ = 1.8 MeV) and transform into lead-209. The latter finally arrives at stable bismuth-209 through β^−^ emission (Eβ = 0.6 MeV). The gamma emissions generated following the disintegration (440 keV, 26.1% emission probability) could be suitable for SPECT imaging and in vivo dosimetry [14].

The energy of the α particle emitted by the polonium-213, corresponding to an 85 μm path length in human soft tissue, is the major contributor to the total α emitted energy per disintegration and is primarily responsible for cytotoxic effects in Bismuth-213 TAT (targeted alfa therapy) [14,18].

**Figure 3:**
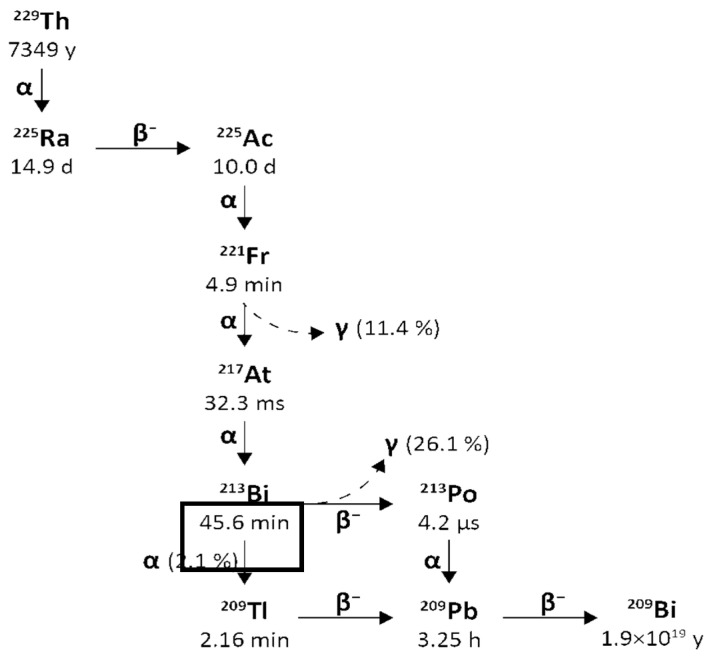
Decay chain of Thorium-229 to Actinium-225 and Actinium-225 to Bismuth-213.

Figure 3 reports a schematic representation of the decay chain of Thorium-229 to Actinium-225 and Bismuth-213.

To produce the short-lived Bismuth-213 (T1/2 = 45.6 min) on-site, Actinium-225 can either be used directly as a therapeutic nuclide or set into Actinium-225/Bismuth-213 generators [11]. The development of several types of these generators was based on selective separation of Bismuth-213 using cation and anion exchange or extraction chromatography [11].

##### Actinium-225/Bismuth-213 Radionuclide Generators

To produce the short-lived Bismuth-213 (T1/2 = 45.6 min) on-site, Actinium-225 can either be utilised directly as a therapeutic nuclide or set into Actinium-225/Bismuth-213 generators [14,25]. All patient investigations with Bismuth-213 up to now have utilised Actinium-225/Bismuth-213 generators. In this well-known approach, the parent Actinium-225 in an acidic solution (for example, 0.05 M HNO_3_) is tightly bound by the sorbent (for example, AG MP-50 cation exchange resin), and Bismuth-213 is eluted [25]. To obtain Bismuth-213 in the forms of [^213^Bi]BiI_4_^−^ and [^213^Bi]BiI_5_^2−^ that may be employed immediately for radiochemistry uses, elution is often conducted with a mixture of 0.1M HCl/0.1M NaI. Furthermore, elution is permitted by the Actinium-225/Bismuth-213’s transitory equilibrium roughly every 3 h [25,26].

The high-activity generator technology created at JRC Karlsruhe enables the generator to function reliably, even with up to 4 GBq of Actinium-225 activity [14,25]. The yields of Bismuth-213 elution may be greater than 80%, while the parent nuclide (Actinium-225) penetration through the generator (breakthrough) is less than 0.2 ppm (parts per million) in activity.

A way to minimise organic resin’s radiolytic degradation and ensure its stable performance over several weeks is the process of the homogeneous distribution of Actinium-225 activity over roughly two-thirds of the generator resin [15,26].

Injection-ready therapeutic dosages of Bismuth-213-labelled peptides with an activity of up to 2.3 GBq have been successfully prepared using the generator in clinical applications [25], for example, in the case of locoregional treatment of the brain [15]. Due to the relatively long parent half-life, which enables the generator to be transported to radiopharmacy facilities over vast distances, these generators may be employed clinically.

#### 2.2.2. Radiochemistry

Bismuth-213 is a radiometal that requires a chelator with an extra reactive functional group to create a covalent connection with the vector molecule and, therefore, a stable complex [18].

Considering the short half-life of the radionuclide and the radioprotection requirements, it is also essential to use chelators suitable for fast and mild radiolabelling conditions to facilitate the manufacturing practice and the manipulation of Bismuth-213 radiopharmaceuticals. The possibility of using Bismuth-213 alone, chelator-free, is discouraged since it accumulates in the kidneys [67,68].

Due to the electronic configuration of [Xe] 4f14 5d10 6s2 6p3, the (+III) oxidation state is the most prevalent form of the bismuth ion, even though (+V) species have been described in some situations. As a hard Lewis acid, it strongly attracts hard donor atoms like oxygen or nitrogen, implying that chelating agents like amino polycarboxylate ligands would form stable complexes with Bi(III) [11].

Bismuth-213 can be stably linked to biomolecules via derivatives of DTPA (diethylene triamine pentaacetic acid) or DOTA (1,4,7,10-tetraazacyclododecane-1,4,7,10-tetraacetic acid). The former shows rapid radiolabelling capability at room temperature. Still, it may have lower stability than DOTA, i.e., a higher risk of compound dissociation and unwanted biodistribution of unbound Bismuth-213 in the body.

**Figure 4:**
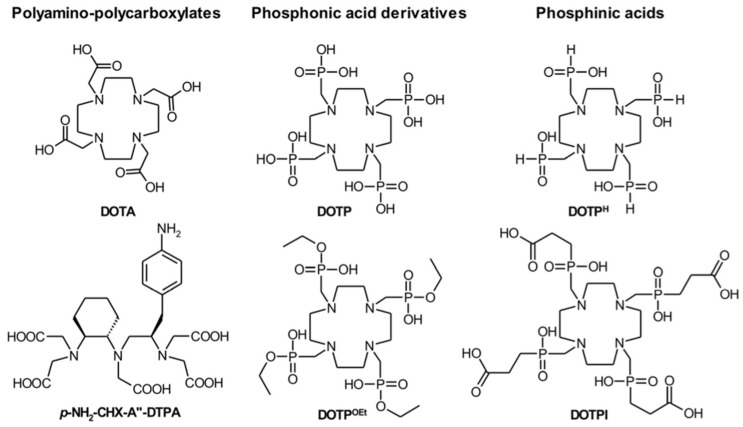
Chelators investigated for Bismuth-213.

Given that Bismuth-213 has a short half-life, reaction time is a critical parameter in radiolabelling chemistry. This was not an issue because of the quick complexation kinetics of DTPA (5 min at room temperature). However, in the case of DOTA complexation, high temperatures and a longer reaction time (30 min) are frequently necessary. Still, these conditions have been overcome by developing a radiolabelling procedure utilising microwaves that allows the complexation of Bismuth-213 in about 5 min at 95 C at pH = 9 [14]. The macrocyclic DOTA chelator is the gold standard bifunctional chelator, allowing for a compound stability of at least two hours [22]. Recently, cyclen-based chelators bearing phosphonic or phosphinic arms were described to form Bismuth-213-complexes in suitable reaction conditions (5 min, 25 °C or 95 °C, pH = 5.5), with high RCYs and promising stability at lower ligand concentration than DOTA or DTPA derivatives [11,69]. Chelators investigated in terms of Bismuth-213 complexation properties are reported in Figure 4 [69].

**Figure 5:**
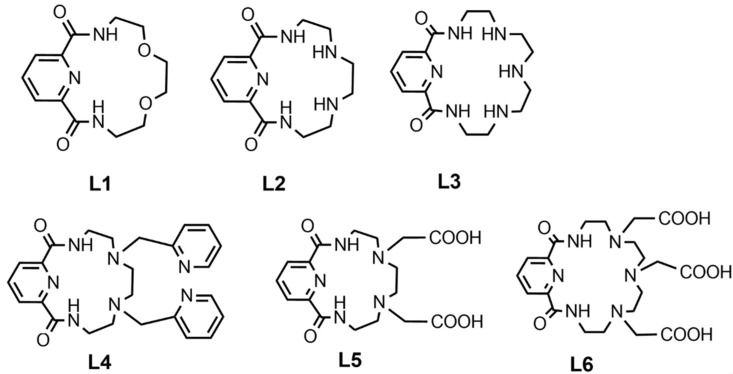
Pyridine-containing azacrown ethers for Bismuth-213.

Alternatively, pyridine-containing azacrown ethers (Figure 5) showed similar results with fast complexation under mild conditions. Among these compounds, the results of in vitro serum stability and in vivo biodistribution studies suggest that the ligand L6 could be promising as a bifunctional chelator for radiopharmaceuticals labelled with Bismuth-213 [70].

#### 2.2.3. Preclinical Studies

Bismuth-213 was one of the first α-emitters to be studied, and the initial in vitro investigations at the beginning of the ‘90s have highlighted the potential of α-particles toward malignant cells [11]. The possibility of using antigen-specific nanobodies, e.g., antibody fragments, as vehicles in TAT has also been explored in the last few years. Their major advantages include an efficient targeting capability and tumour penetration due to their small size, a fast clearance and pharmacokinetics suitable with the short half-life. Administration of the radiocomplex alone and in conjugation with trastuzumab led to an increased survival and low uptake values were observed in normal tissue.

A list of relevant preclinical studies involving Bismuth-213 is shown in Table S3 (Supplemental Data).

#### 2.2.4. Clinical Studies

Bismuth-213 was the first α-emitter to reach the clinical phase with the preparation of [^213^Bi]Bi-lintuzumab for treating AML. In a Phase I trial, 18 patients with relapsed or refractory AML were treated with 10.36 to 37 MBq/kg of [^213^Bi]Bi-lintuzumab. A rapid uptake was noticed in bone marrow, liver, and spleen, privileged sites of leukemic cells. Absorbed dose ratios between these areas and the whole body were measured to be 1000 times more important than analogue radioimmunoconjugates with β-emitters. Even if no complete remission was detected, a significant reduction in marrow blasts was noticed in 14 patients [57].

A phase I/II complementary study demonstrated that sequential administration of cytarabine before treatment with [^213^Bi]Bi-lintuzumab injected doses (18.5 to 46.25 MBq/kg) could induce complete remission in some patients. These results are attributed to the cytarabine’s ability to reduce tumour volume, improving the impact of radiation of [^213^Bi]Bi—lintuzumab [71].

Thereafter, other applications and biological targets were investigated, but these data are not reported in the clinical trial database (ClinicalTrials.gov):

- Bismuth-213-radioimmunoconjugates (Bismuth-213-RICs) were also investigated for therapy of malignant melanoma [72].
- [^213^Bi]Bi-cDTPA-9.2.27 showed an inhibitory effect on metastatic melanoma and no toxicity over the range of administered activities (55–947 MBq) [73,74].
- [^213^Bi]Bi-HuM195 was also successfully attempted for acute myelogenous leukaemia or chronic myelomonocytic leukaemia (CML), involving: 93% of the treated patients had reductions in circulating blasts, and 78% experienced a decline in bone marrow blasts, with no significant extramedullary toxicity reported [57].
- [^213^Bi]Bi-PSMA-617 for mCRPC, resulted in imaging response and a decrease in prostate-specific antigen levels, and [^213^Bi]Bi-DOTATOC in neuroendocrine tumours refractory to beta emitter ^177^Lu/^90^Y-DOTATOC, which led to a significant reduction in targeting agent uptake, i.e., probable reduction in lesion size [58].

##### Locoregional Administration

A pilot study on the feasibility of Bismuth-213 radioimmunoconjugates (Bismuth-213-RICs) for the treatment of carcinoma in situ of the bladder refractory to bacillus Calmette–Guérin was conducted in 12 patients, showing no toxicity and a complete response for three patients, 44, 30 and 3 months after the administration [75]. Bismuth-213-DOTA-Substance P was locally injected in patients with recurrent glioblastoma multiforme. Treatment was safe and well tolerated, and median survival was superior to other alternative therapies [76]. A third example of locoregional injection of Bismuth-213-radiopharmaceutical was performed in the treatment of metastatic skin melanoma: significant reduction in serum marker melanoma-inhibitory-activity protein (MIA) at 2 weeks post-TAT was observed, and the therapy was safe and well tolerated [72].

A list of relevant clinical studies involving Bismuth-213 is shown in Table S4 (Supplemental Data).

#### 2.2.5. Conclusions

Using Bismuth-213 bound to antibodies, peptides, and nanobodies showed optimal results in preclinical and clinical studies, with increased median survival and no relevant signs of toxicity. Bismuth-213 radiopharmaceuticals were tested and demonstrated efficacy in various malignancies, such as acute myelogenous leukaemia, mCRPC, and neuroendocrine tumours refractory to beta emitters.

### 2.3. Astatine-211

Astatine is the rarest of all naturally occurring elements on Earth, situated below iodine in the periodic table. While only short-lived isotopes (t1/2 ≤ 8.1 h) are known, astatine-211 is the object of growing attention due to its emission of high-energy alpha particles. The interest in applications of Astatine-211 in nuclear medicine translates into the increasing number of cyclotrons able to produce it. Yet, many challenges related to the minute amounts of available astatine must be overcome to characterise its physical and chemical properties. This point is of great importance for developing synthetic strategies and addressing the instability of the labelled compounds under the current approach, which limits the use of Astatine-211-labelled radiopharmaceuticals. Despite its discovery in the 1940s, only the past decade has seen a significant rise in understanding astatine’s basic chemical and radiochemical properties, thanks to the development of new analytical and computational tools.

#### 2.3.1. Physical Characteristics

Astatine-211 decays with a 7.21 h half-life. It decays by 58.2% electron capture to short-lived Polonium-211 (T1/2 = 0.516(3) s) that decays in turn with 100% α emission to stable Lead-207. Moreover, Astatine-211 decays by 41.8% α emission to quasi-stable Bismuth-207 (T1/2 = 32.9 years; only 26 Bq Bismuth-207 per 1 MBq of Astatine-211 after decay of the latter) that decays in turn to stable Lead-207. Including its short-lived Polonium-211 daughter, the cumulative α emission is 100% per Astatine-211 decay with an average α energy of 6.78 MeV.

**Figure 6:**
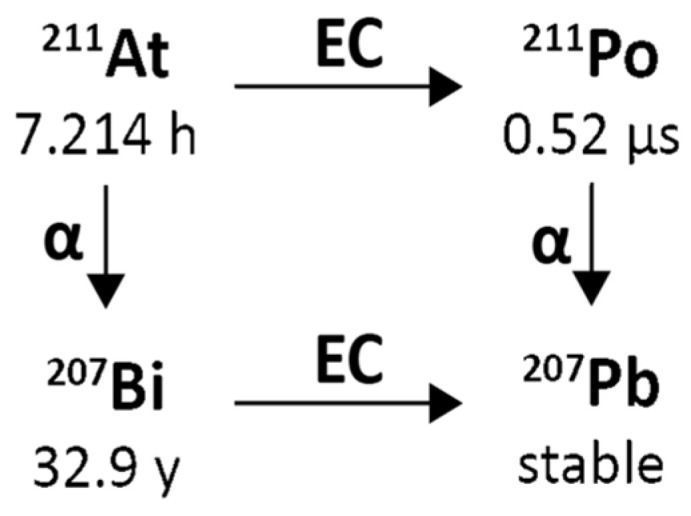
Simplified decay chain of Astatine-211.

The mean α energy per decay is 6785 keV, the mean recoil energy (of Bismuth-207 or Lead-207 recoils, respectively) is 131 keV, the mean electron energy per decay is 3 keV, and the mean photon energy per decay is 43 keV. Figure 6 reports a simplified Astatine-211 decay scheme illustrating the double-branched pathway: by direct alpha decay to Bismuth-207 and by electron capture to Polonium-211, followed by alpha decay to Lead-207.

An essential characteristic of Astatine-211 that is different from most other α-emitters of relevance to Targeted Alpha Therapy (TAT) is that it yields one α-particle per decay, which offers certain translational advantages, including simplification of radiation dosimetry calculations for Astatine-211-labelled TAT agents. Fortuitously, 58.2% of Astatine-211 decays occur via electron capture to polonium-211, producing 77–92 keV polonium x-rays that permit counting Astatine-211 activity with conventional gamma detectors and quantification of Astatine-211 distribution in vivo by planar and SPECT imaging [65]. These x-rays allow measurement of Astatine-211 biokinetics in patients, which can be used for safety and stability monitoring and organ-level assessment of radiation dosimetry of actual treatment doses. The Polonium-211 then decays with a 0.516 s half-life by emission of a 7.45 MeV α-particle to Lead-207, which is stable. The second Astatine-211 decay branch (41.8%) involves the emission of 5.87 MeV α-particles to Bismuth-207 (T1/2 = −32.9 y), which likewise decays (by electron capture) to lead-207.

Two aspects of the Astatine-211 decay scheme could potentially be problematic—the Polonium-211 intermediate and the long-lived Bismuth-207 intermediate stated above. Concerning the first, one must consider the effects of an initial nuclear decay event on the fate of a subsequent α-particle emission. In the decay schemes where the first decay event is also by α-emission, the daughters would undergo chemical transformation, and the α-particle recoil energy would lead to escape and migration from the original decay site [1]. On the other hand, in Astatine-211 decay, the Polonium-211 intermediate is the progeny of electron capture decay, which involves chemical transformation but insignificant daughter recoil energy. Even with the worst possible case assumptions—that this chemical transformation results in an instantaneous release of Polonium-211 from the cell surface and transport by unimpeded thermal diffusion—nearly 100% of Polonium-211 atoms should decay within two cell diameters from the original cell surface [77]. Since electron capture results in a highly charged daughter nucleus [78], which could impede diffusion, the diffusion distance from the original Astatine-211 decay site might be even shorter. In any case, except in the rare instance where cancer presents for treatment as a single-cell distributed disease, the diffusion of the Polonium-211 daughter from the original decay site can be ignored. Second, the long half-life of the Bismuth-207 daughter could lead to potential issues due to its uptake in the bone, liver, and kidneys. However, this should also not be of concern because nearly 100,000 decays of Astatine-211 are needed to produce a single decay of Bismuth-207 [79]. Thus, a 370 MBq (10 mCi) hypothetical patient dose of Astatine-211-labelled TAT agent would generate ~4 kBq (~0.1 μCi) of Bismuth-207, a level that is only 0.1% of the 100 μCi Annual Limit of Intake (ALI) recommended for Bi-207 by the Nuclear Regulatory Commission, making its potential toxicity negligible.

The α-particle emission of Astatine-211, with a mean linear energy transfer of about 100 keV μm−1, is like other TAT-candidate radionuclides [80]. This results in high relative biological effectiveness (RBE) because they can create more than 10 ionizations in a 100 Å diameter × 3 nm column [61,81], an ionisation density close to the diameter of the DNA double helix. Even though Astatine-211 emits fewer α-particles per decay than some other radionuclides under investigation for TAT, Astatine-211-labelled targeted radiotherapeutics are exquisitely cytotoxic, with effective killing achievable with less than 10 atoms bound per cell. Moreover, if uptake of Astatine-211 in the cell nucleus can be achieved, the fact that Astatine-211 also generates an average of 6.2 Auger electrons per decay (comparable to Gallium-67) might be of therapeutic benefit [82].

#### 2.3.2. Radiochemistry

One of Astatine-211’s most attractive properties, contributing to the emerging demand for this radionuclide, is the spectrum of targeting agents compatible with its labelling chemistry and physical half-life [83]. In contrast to other α-emitters, astatine is a halogen with similar chemical properties to iodine, albeit with more metalloid properties [84]. Astatine can exist in several oxidation states [85], providing multiple synthetic options but contributing to its sometimes confounding, capricious behaviour [86].

Although significant differences exist between astatine and iodine in labelling chemistry, carbon–halogen bond strength, lipophilicity, and the in vivo stability of carbon–halogen bond, they are involved in the most common strategy to develop Astatine-211-labelled TAT agents to build on previous studies with radioiodine. Suppose one can demonstrate similar in vivo behaviour for the two labelled compounds. In that case, this suggests the possibility of using a radioiodinated analogue (I-124 for PET, I-123 for SPECT) as an imaging theragnostic partner for the corresponding Astatine-211-labelled therapeutic. Astatine-211 can readily be incorporated by direct substitution into small organic molecules, a potential advantage compared with radiometals, which require somewhat bulky polydentate ligands for stable incorporation. Two of the widely investigated approaches for Astatine-211-labelling are the electrophilic demetallation of tin and silicon precursors [87] and carboranyl precursors. Recently, a novel approach for Astatine-211-labelling that involves Cu-catalysed astatination of boronic esters was demonstrated to have broad applicability, including the labelling of a PARP inhibitor [88]. Another method used a sulfonyl precursor for labelling neopentyl derivatives, providing high in vivo stability against nucleophilic substitution or Cytochrome P450 (CYP) metabolism [89].

As is the case with radiometals, biomolecules, including monoclonal antibodies, affibodies, diabodies, and nanobodies, can be labelled with Astatine-211 using a variety of procedures [90]. For example, this can be accomplished via either the prototypical acylation agent N-succinimidyl 3-[^211^At] astatobenzoate [91], using thiol-Michael addition for site-specific conjugation [92], or N-succinimidyl 3-[^211^At] astato-5-guanidino methyl benzoate. This prosthetic agent provides intracellular radioactivity trapping after internalising receptor-targeted vectors [93,94].

Finally, the remarkable affinity of Astatine-211 for gold has permitted the direct and nearly quantitative Astatine-211-labelling of gold nanoparticles, which can be used alone or with targeting vectors decorating their surface [95]. In this account, we give a concise summary of recent advances in the determination of the physicochemical properties of astatine, putting in perspective the duality of this element, which exhibits the characteristics of both a halogen and a metal. Striking features were evidenced in the recent determination of its Pourbaix diagram, such as identifying stable cationic species, At+ and AtO+, contrasting with other halogens. Like metals, these species were shown to form complexes with anionic ligands and to exhibit a particular affinity for organic species bearing soft donor atoms. On the other hand, astatine shares many characteristics with other halogen elements. For instance, the At– species exists in water but with the least EH–pH stability range in the halogen series. Astatine can form molecular interactions through halogen bonding, and it was only recently identified as the strongest halogen-bond donor. This ability is nonetheless affected by relativistic effects, which translate to other peculiarities for this heavy element. For instance, the spin-orbit coupling boosts astatine’s propensity to form charge-shift bonds, catching up with the behaviour of the lightest halogens (fluorine, chlorine).

All these new data impact the development of radiolabelling strategies to turn Astatine-211 into radiopharmaceuticals. Inspired by the chemistry of iodine, the chemical approaches have sparsely evolved over the past decades and have long been limited to electrophilic halo-demetallation reactions to form astatoaryl compounds. Conversely, recent developments have favoured using the more stable At– species, including the aromatic nucleophilic substitution with diaryliodonium salts or the copper-catalysed halodeboronation of arylboron precursors.

However, new bonding modalities are necessary to improve the in vivo stability of Astatine-211-labelled aryl compounds. The tools and data gathered over the past decade will contribute to instigating original strategies for overcoming the challenges offered by this enigmatic element. Alternatives to the C–At bond, such as the B–At and the metal–At bonds, are typical examples of exciting new research axes [96].

#### 2.3.3. Preclinical Studies

Extensive preclinical work has been performed in the past on different types of malignancies using Astatine-211-labelled compounds. Different targeting agents were used for evaluation, e.g., small molecules, peptides, antibodies, or fractions of Ab and nanoparticles. In the field of prostate cancer, several different approaches were tested in the preclinical setting. Urea-based PSMA-targeted (2S)-2-(3-(1-carboxy-5-(4-[^211^At]astatobenzamido) pentyl) ureido)-pentanedioic acid significantly improved survival in mice bearing PC micrometastases after systemic administration.

List of relevant preclinical studies of Astatine-211-labelled compounds is shown in Table S3 (Supplemental Data).

#### 2.3.4. Clinical Studies

The promising physical characteristics of the radionuclide led to early translation into clinical studies. The early studies were typically performed in a compassionate setting with exhausted available therapeutic options. Additional information about many of the most promising Astatine-211-labelled radiopharmaceuticals that have been investigated for TAT applications, including those that have been evaluated in patients, can be found in several reviews.

As early as 1954 (less than 15 years after the discovery of Astatine-211), the biodistribution of the radionuclide was investigated in a small series of 7 patients with thyroid disorders and a single patient with a locally advanced papillary adenocarcinoma of the thyroid [97]; while there was evident accumulation of the radionuclide in the thyroid gland after surgery, no accumulation was found in the regional lymph node metastases. After a substantial time gap, the radionuclide was used in 1990 to treat an unresectable relapsed carcinoma of the tongue using an intraarterial injection of Astatine-211-labelled HSA microspheres, causing local necrosis of the tumour, later spreading to the rest of the tongue [98]. A series of patients (altogether 18) was treated in 1990 for the recurrence of glioma by injecting [^211^At]At-labelled anti-tenascin molecule directly into the tumour cavity; time to progression was superior to the reports from the literature, including no physiological side effects [99]. Twelve patients with peritoneal metastases of ovarian carcinoma were treated in 2009 with [^211^At]At -labelled antibodies against NaPi2b [65].

Astatine-211-labelled antibody OKT10-B10 targets CD45 on several haematological malignancies and is being tested in patients with multiple myeloma, myelodysplastic syndrome, and several types of acute leukaemia, as well as a conditioning method before hematopoietic stem cell transplantation in non-malignant conditions to reduce graft rejection [100,101]. Meta-[^211^At]At-benzyl guanidine (MABG) is expected to surpass the effectiveness of the [^131^I]I-labelled alternative in systemic targeted therapy of metastatic pheochromocytoma/paraganglioma; up to 18 patients are planned [101]. Finally, Astatine-211 is being investigated as an alternative to 131I in patients with differentiated thyroid cancer [102].

List of clinical studies involving Astatine-211 reported in the database ClinicalTrials.gov is shown in Table S4 (Supplemental Data).

#### 2.3.5. Conclusions

An important characteristic of Astatine-211 that is different from most other α-emitters relevant to Targeted Alpha Therapy (TAT) is that it yields one α-particle per decay, which offers certain translational advantages, including simplification of radiation dosimetry calculations for Astatine-211-labelled TAT agents.

Despite the potential of Astatine-211, the research on this radionuclide remains limited to a few centres because of challenges in production and chemistry. The most significant factor affecting the supply of Astatine-211 is the limited availability of suitable accelerators. Moreover, the short half-life of Astatine-211 restricts delivery.

### 2.4. Lead/Bismuth 212 Pair

#### 2.4.1. Physical Characteristics

**Figure 7:**
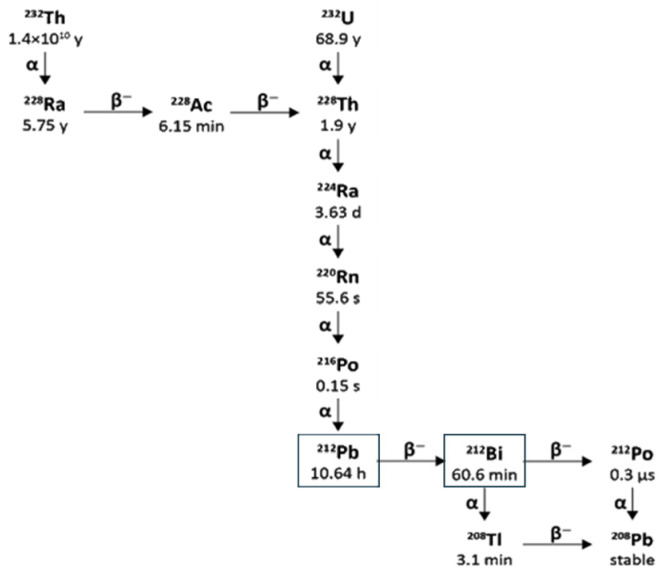
Decay scheme of thorium-232 and uranium-232 to Lead-212/Bismuth-212.

Lead-212 has a half-life of 10.64 h and decays through β^−^ β-emission to bismuth-212, which in turn has a half-life of 60.5 min and decays through two pathways, each with one β^−^ and one α-decay, as shown in Figure 7.

The bismuth-212 β^−^ decay (64% probability) produces polonium-212 with a half-life of just 0.3 µs, which decays by α emission to stable lead-208. The other bismuth-212 decay route (36% probability) is through α emission to thallium-208 with a half-life of 3.05 min, which decays to lead-208 via a β^−^ emission.

There are also significant gamma emissions for the Lead-212 decay scheme that have implications for detection, handling, and safety. Lead-212 emits at 238.6 keV and 300.1 keV, allowing for its detection in lab radiochemistry and use in clinical gamma scintigraphy or SPECT/CT, which supports quantitative measurement and dosimetry. The decay product thallium-208 emits a very high-energy gamma (2614 keV) with 99.75 photons produced per 100 disintegrations. The short half-life of thallium-208 of just 3.05 min also contributes to a high abundance of these high-energy emissions, which requires 15.5 mm of lead shielding to attenuate the radiation by half [103,104]. Therefore, it is essential to have adequate measures to ensure that operators minimise their exposure by reducing the time of operations, increasing their distance from the source, and using sufficient shielding. As thallium-208 is a decay product of Lead-212, the amount present in a source of pure Lead-212 will increase over time until equilibrium is reached with its progeny, which takes approximately 4 h [105]. This also has implications for measuring the activity of purified Lead-212 present using an isotope calibrator, as the activity displayed will increase over time, and a calculated factor must be applied to the reading to accurately determine the activity of Lead-212 present in a sample [106]. When developing novel molecular radiotherapy agents, appropriate screening methods and accurate dosimetry are vital, so the existence of suitable radioisotopes that can be used for imaging is an advantage. Lead-203 offers this for Lead-212. Lead-203 has gamma emissions at 279 keV and a half-life of 51.9 h, making it suitable for gamma cameras and SPECT imaging [107]. It can also be produced on a cyclotron from a solid thallium target using the ^205^Tl(p,3n)^203^Pb nuclear reaction with >20 MeV protons [108].

#### 2.4.2. Radiochemistry

Lead is a group 14 metal with a preferred oxidation state of 2+ and is amenable to chelation [109]. Although Lead-212 is used for targeted alpha therapy, it does not decay by alpha emission itself but by beta emission to yield the alpha emitter bismuth-212, as discussed above. To ensure both Lead-212 and Bi-212 are targeted and accumulate in disease sites, it is ideal for chelators of Lead-212 to remain bound to metals upon decay. Lead-212 has an advantage over alpha-emitting radionuclides in this respect, as the recoil energy of beta emission is in the range of 1 eV [6] compared to the 100,000 times higher recoil energy of an alpha emission (for example, the 212Bi alpha recoil energy is 108–117 keV [110]). While the recoil energy of an alpha emitter will undoubtedly break any bond between a chelator and a metal centre, the 1 eV recoil energy of beta particle emission is insufficient. However, dissociation of the 212Bi from the chelator can occur due to the sudden change in nuclear charge resulting from [^212^Pb]Pb^2+^ converting into [^212^Bi]Bi^3+^ or [^212^Bi]Bi^5+^ and the consequential valence electron shell reorganisation [6]. Experimentally, this has been observed for the DOTA chelator: when [^212^Pb]Pb-DOTA decays to [^212^Bi]Bi-DOTA, 36% becomes unchelated [107]. As Bismuth-212 has a 60.5 min half-life, in vivo redistribution of a proportion of free Bi-212 could occur if no additional cell trapping mechanisms, such as internalisation and residualisation, are at play. However, this is minor compared to the redistribution seen after alpha emission, and appropriate chelator design can potentially mitigate such issues for Lead-212.

**Figure 8:**
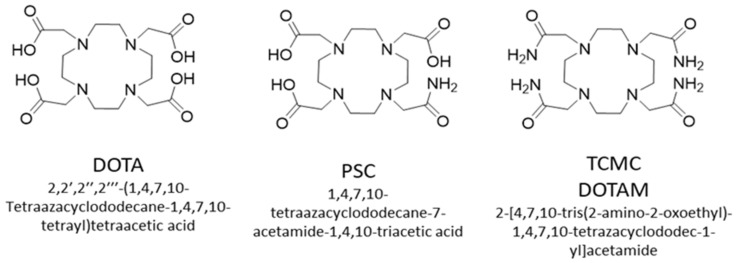
Chemical structures of DOTA, PSC and TCMC chelators.

Chelators suitable for Lead-203/Lead-212 include DOTA, TCMC (DOTAM) [109] and PSC [111] (Figure 8). These complexes will have a different charge when bound to lead (II), which is expected to alter the biodistribution of their corresponding bioconjugates. If one of the carboxyl groups were used for conjugation, the following complexes would result ([Pb(II)-DOTA-bioconjugate]1-[Pb(II)-TCMC-bioconjugate]1+, [Pb(II)-PSC-bioconjugate]0).

Unlike other radionuclides, which are predominantly produced via irradiation using either a research reactor or an accelerator, Lead-212 is instead isolated from its parent radionuclides to create either a Radium-224/Lead-212 generator or a Thorium-228/Lead-212 generator. Both Radium-224 and Lead-212 are part of the Uranium-232 and Thorium-232 decay chain, in which some stockpiles have been created from naturally occurring uranium or previous civil or defence nuclear activities [107]. Several generators are under development with varying designs [112], but currently, the most widely available for research use is the Radium-224/Lead-212 generator from the United States Department of Energy Isotope program. This ion exchange generator contains AG MP-50 resin, loaded with up to 600 MBq of Ra-224 with a radionuclidic purity of >99.9%. The generator is eluted in hydrochloric acid. However, chloride salts of lead are poorly soluble, making radiolabelling difficult, so the first radiochemistry step is often the conversion to lead nitrate or lead acetate, which is achieved via evaporation and extraction, or solid phase extraction, respectively [112].

#### 2.4.3. Preclinical Studies

Early pre-clinical studies with Lead-212 focused on radiolabelling antibodies as proof of principle. However, the 10.6 h half-life of lead-212 is better suited to the biological half-life of peptides and small molecules, and recent work has moved to focus on lead-212 radiopharmaceuticals that utilise these molecules. List of relevant preclinical studies of lead-212-labelled compounds is shown in Table S3 (Supplemental Data).

#### 2.4.4. Clinical Studies

The first phase 1 clinical trial using Lead-212 was a trastuzumab bioconjugate labelled with Lead-212 via a TCMC chelator (NCT01384253) [113]. This was a safety and dose-escalation study where 18 patients with relapsed human epidermal growth factor receptor-2 (HER2) expressing peritoneal metastases were treated with a single intraperitoneal infusion of [^212^Pb]Pb-TCMC-Trastuzumab and the agent was shown to be safe at all administered activities (7.4–27.4 MBq/m^2^, total activity 15–40 MBq) [113].

The first trial evaluating systemic, intravenously administered Lead-212-radiolabelled peptides was conducted using the Alphamedix bioconjugate [^212^Pb]Pb-TCMC-TATE from RadioMedix and OranoMed, which targets somatostatin receptor-positive neuroendocrine cancers. The results of the phase 1 trial [114] of [^212^Pb]Pb-TCMC-TATE (NCT03466216) were reported in 2021, and a phase 2 study is now open (NCT05153772). In the phase 1 trial, the highest administered activity was 2.5 MBq/Kg (max activity per cycle 203.5 MBq) for up to 4 cycles 8 weeks apart (maximum total activity per subject 888 MBq). The dosing regime was determined from a previous clinical study using [^203^Pb]Pb-TCMC-TATE (unpublished), but patients for this study were screened with [^68^Ga]Ga-DOTA-TATE [114]. This treatment was well tolerated with no severe treatment-emergent adverse events related to the study drug, and objective radiological responses were seen for 8 of 10 subjects treated at the highest activity regime for four cycles. Information about the dose delivered by this radiopharmaceutical to disease sites or dose-limiting organs is not yet available; dosimetry data were collected for six subjects enrolled in this trial and will be reported separately [114].

OranoMed has also opened a phase 1 clinical trial (NCT05283330) assessing the safety and tolerability of [^212^Pb]Pb-DOTAM-GRPR1 in adults with cancers that express the gastrin-releasing peptide receptor BBR (Cervical Cancer, Prostate Cancer, Metastatic Breast Cancer, Colon Cancer, Non-Small Cell Lung Cancer, Cutaneous Melanoma). In 2023, two new early-phase trials have started recruiting to evaluate Lead-212 radiopharmaceuticals in metastatic castration-resistant prostate cancer: A) NCT05720130, a phase 1/2 safety and efficacy study of [^212^Pb]Pb-ADVC001, for which AdvanCell Isotopes Pty Limited is the sponsor. This is being conducted in Australia, and B) NCT05725070, a Phase 0/1 Study, in Norway of [^212^Pb]Pb-NG001, for which ArtBio is the sponsor.

More recently, Pb-203 is being used to scope the potential for new Lead-212 radiopharmaceuticals:

- [^212^Pb Pb-VMT-α-NET ([^212^Pb]Pb-PSC-PEG2-TOC) for somatostatin expressing neuroendocrine tumour (NCT06479811, NCT06427798)
- [^212^Pb]Pb-VMT01 ([^212^Pb]Pb-DOTA-PEG2-α-MSH for melanoma tumours expressing the melanocortin sub-type 1 receptor (MC1R) (NCT05655312) [115].

List of clinical studies involving Lead-212 reported in database ClinicalTrials.gov is shown in Table S4 (Supplemental Data).

#### 2.4.5. Conclusions

Lead-212 is of high interest for TAT as an in situ generator of the alpha emitter Bi-212 due to its well-matched half-life for peptide-based targeting moieties and the opportunity to conduct dosimetry studies with Pb-203. However, its most significant advantage is likely to become its wide availability due to stockpiles of its parent radionuclides and the investment in programmes to develop Thorium-228/Lead-212 and Radium-224/Lead-212 generators. Currently, there is limited preclinical and clinical data with Lead-212, but the results obtained so far are very promising.

The short half-life of Lead-212 offers substantial clinical advantages (patient and waste management) in comparison with Actinium-225. To manage the secondary α-emission of Lead-212, the stability of Bismuth, improved chelator system and different incorporating strategies should be developed.

### 2.5. Terbium-149

The concept of using terbium-149 for potential α-therapy and terbium-152 for imaging/dosimetry was proposed by Beyer and Allen et al. in the late 1990s [116,117] and was further pursued by addressing the potential of terbium radioisotopes towards theranostics. The quadruplet of terbium radionuclides, i.e., terbium-152 (T1/2 = 17.5 h; PET) and terbium-155 (T1/2 = 5.3 d; SPECT) for imaging, while Terbium-149 (T1/2 = 4.1 h; α-emitter) and terbium-161 (T1/2 = 6.9 d; β-emitter) proposed as potentially effective for radionuclide therapy, are recommended as true theranostic radiometals [118].

Terbium-149 represents a powerful alternative to the currently employed α-emitters [119]. These physical properties make it particularly well suited for application with small-molecular-weight targeting agents, including peptides, which are quickly cleared from the body [120]. The absence of α-emitting daughters is regarded as an additional favourable feature of Terbium-149 since the toxicity of α-emitters with multiple α-emitting daughters has been identified as an issue for clinical application [1]. In vivo use of Terbium-149 appears feasible, as it does not pose a risk of harmful α-particle emission from released daughter radionuclides. The decay scheme of Terbium-149 is complex [121], and the potential radiotoxicity of the resulting radio lanthanides remains to be determined [121].

#### 2.5.1. Physical Characteristics

**Figure 9:**
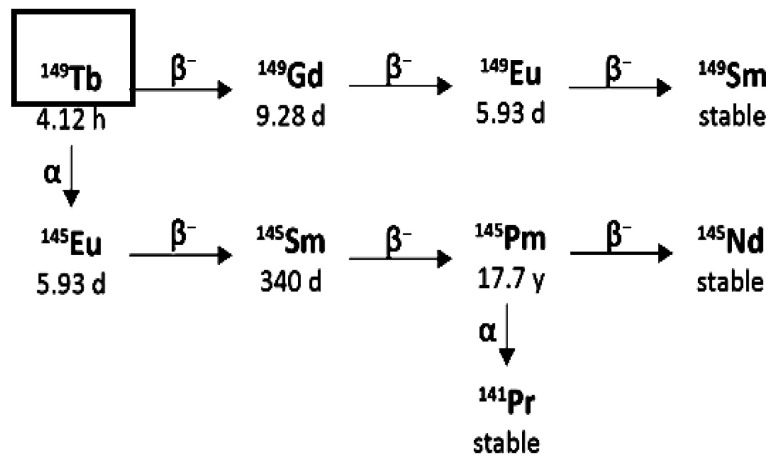
Simplified decay chain of Terbium-149.

Terbium-149 decays in a complex decay scheme [4] with a half-life of 4.12 h, by emitting predominantly low-energy alpha-particles (3.97 MeV, 17%), EC-process (76%) and β+-emission (7%). Alpha-particle tissue range is around 25 μm and the LET of 140 keV/μm. The absence of alpha-emitting daughters is a favourable feature of Terbium-149 for clinical applications [1]. However, the daughter products of Tb-149 are long-lived radionuclides, like Gadolinium-149 (9.28 d), Europium-145 (5.93 d), Samarium-145 (340 d), Europium-149 (93.1 d), etc. More research is required to elucidate any complexity arising due to the in vivo presence of these Terbium-149 decay products (Figure 9).

#### 2.5.2. Radiochemistry

As a trivalent radiolanthanide, Terbium-149 can be stably coordinated with the conventional macrocyclic 1,4,7,10-tetraazacyclododecane-1,4,7,10-tetraacetic acid (DOTA) chelator [118,122]. These circumstances allow the use of Terbium-149 with DOTA-functionalized compounds that are (pre)clinically established for ^177^Lu-based radionuclide therapy. Thus, existing approaches for labelling chelated bioconjugates with Lutetium-177, as well as with Holmium166, Samarium-153, Bismuth-213 or Actinium-225, can be directly applied to Terbium-149.

Reactions to produce Terbium-149 have been suggested using protons [123] and heavy ions [124,125,126], with reviews available [116,127,128].

Terbium-149 was produced in the spallation reaction Ta(p, spall) using the online isotope separator facility ISOLDE at CERN (Geneva, Switzerland) [129]. A tantalum-foil target (120 g/cm^2^) was irradiated with 1.0- or 1.4-GeV protons delivered from the CERN PS-Booster accelerator. The radio-lanthanides generated in the spallation process were released from the target material, which was kept at about 2200 °C, ionised by surface ionisation and accelerated to 60 keV. From the obtained radioactive ion beams, the A = 149 isobars (dysprosium-149, Terbium-149 and molecular ions Ce(Ce-133)O+ and [^133^La]LaO^+^ were implanted (60 keV) and thus collected in thin layers of KNO3 (10 mg/cm^2^) on aluminium backings. The Terbium was separated from its daughters (Gadolinium-149 and Europium-145) and the pseudo-isobars Cesium-133 and Lantanum-133 by cation exchange chromatography using Aminex A5 resin and α-hydroxyisobutyric acid as eluent. The Terbium-149 fraction (150–200 μL) was evaporated to dryness and re-dissolved in 50 μL of 100 mM HCl. The final Terbium-149 concentration was 2 GBq/mL (54 mCi/mL) at the end of chromatographic separation (EOS).

Production of Terbium-149 in the nuclear reactions ^152^Gd(p,4n)^149^Tb and ^142^Nd(12C,5n)^149^Dy → Terbium-149 at the U-200 cyclotron (LNR, JINR, Dubna) [116] was also experimentally confirmed. However, long-lived Eu, Pm, Gd, and Sm radionuclides in the Terbium-149 decay chain and problems with Terbium-149 production are significant drawbacks preventing the routine use of Terbium-149 in nuclear medicine [130].

Recently, Terbium-149 was produced by proton-induced spallation of a tantalum target, followed by an online isotope separation process at ISOLDE/CERN (Geneva, Switzerland). The mass-separated ion beam was implanted into a zinc-coated gold catcher foil, which was shipped to Paul Scherrer Institute (PSI, Villigen-PSI, Switzerland) for processing. As previously reported, Terbium-149 was separated from isobar and pseudo-isobar impurities by cation exchange chromatography [118]. The separation yield was 100 MBq (~99%) of highly pure Terbium-149 in α-hydroxybutyric acid solution (pH 4.7), sufficient for preclinical application. The radiolabelling was carried out directly in the eluent solution by the addition of DOTANOC and incubation of the reaction mixture for 15 min at 95 °C. [^149^Tb]Tb-DOTANOC was obtained with >98% radiochemical purity at a high specific activity (5 MBq/nmol), as confirmed by high-performance liquid chromatography (HPLC)-based quality control [131].

As an alternative, it was proposed to obtain Terbium-149 by irradiating Eu-151 targets with 3He nuclei and the thick target yields in the energy range 70 → 40 MeV were experimentally determined [132,133]. Preliminary results showed that Terbium-149 yields can be high enough to produce therapeutic amounts of radionuclide. The method does not depend on mass separation and has the advantage of the availability of target material. Nevertheless, its drawback is the limited availability of high-intensity 3He beams, and it does not allow Terbium-149 free of impurities, and its possibility of clinical application is yet to be proven [134,135].

#### 2.5.3. Preclinical Studies

To date, there are only a few preclinical data published and available with [^149^Tb]Tb-labelled molecules. Several existing preclinical models have already been successfully tested (antibody labelling in haematological malignancies, folate receptor targeting with potential application in gynaecological, gastrointestinal and renal neoplasms, PSMA receptor targeting in prostate cancer. However, the limited availability of the radionuclide has so far precluded the initiation of clinical trials.

List of relevant preclinical studies involving Terbium-149 is shown in Table S3 (Supplemental Data).

#### 2.5.4. Clinical Studies

Due to the valuable combination of physical characteristics (i.e., helpful alpha emission for therapeutic applications and positron emission for follow-up of distribution and possibly dosimetry), Terbium-149 is one of the most promising radionuclides for clinical translation. The amount of injected activity is crucial for PET imaging. So far, the amount of activity required for therapeutic application in clinics is unknown. The sensitivity of the tumour type and other parameters will critically depend on the targeting agent and the degree of its accumulation in the tumour tissue. Whether the quantity of radioactivity would allow for PET imaging remains to be determined in patients [131]. The radionuclide’s restricted availability has prevented the start of clinical trials; therefore, to date, there are no clinical studies documented in the database on ClinicalTrials.gov.

#### 2.5.5. Conclusions

The unconventional production of Terbium-149 was the main reason why Terbium-149 had not yet reached clinical trials, as stated in several reports previously [13]. Currently, endeavours worldwide are focused on establishing new radionuclide production centres, clearly offering new perspectives for producing radionuclides like Tb, which are dependent on mass separation facilities. Such production centres, which exploit spallation production combined with isotope separation online (ISOL), are already in operation at the Isotope Separator and Accelerator (ISAC) at TRIUMF, Canada’s National Laboratory for Particle and Nuclear Physics (Vancouver, Canada) and at Investigation of Radioactive Isotopes on Synchrocyclotron (IRIS), at the Petersburg Nuclear Physics Institute (PNPI, Gatchina, Russia). Other facilities are in the planning stage or under construction at the Radioactive Isotope Beam Factory (RIBF, East Lansing, MI, USA), at the Belgium Nuclear Research Center ISOL facility (ISOL@MYRRHA, Mol, Belgium) and the Japan Proton Accelerator Research Complex (J-PARC ISOL, Tokai, Japan). MEDICIS, a new radionuclide production centre dedicated to medical applications, is currently being built at CERN (Geneva, Switzerland) [27]. MEDICIS aims to produce medically interesting but not yet thoroughly investigated radionuclides, including Terbium-149, in quantities sufficient to address the requirements of pilot investigations in patients. The perspective of overcoming the obstacle of production holds great promise for more detailed preclinical investigations and first clinical trials shortly using Tb for α- α-therapy, combined with PET.

### 2.6. Radium-223

#### 2.6.1. Physical Characteristics

**Figure 10:**
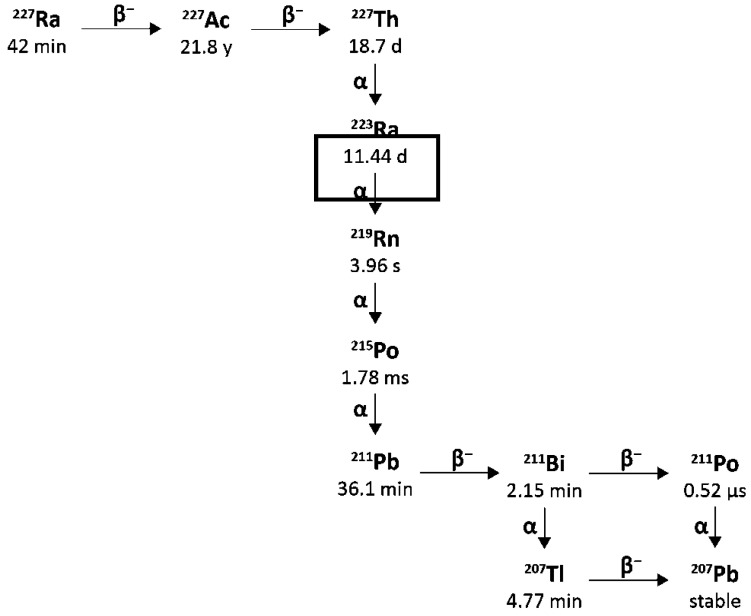
Decay chain of Radium-223.

Radium-223 (half-life of 11.43 days) is formed naturally in trace amounts by the decay of uranium-235. It is usually produced artificially by exposing natural radium-226 to neutrons to produce Radium-227 (half-life of 42 min), which decays to Actinium-227 (half-life of 21.8 years) and then via Thorium-227 (half-life of 18.7 days) to Radium-223. This decay path makes obtaining it from the Actinium-227/Thorium-227 generator convenient. Radium-223 has a complex decay that produces 4 high-energy α particles, 2 β-particles and different Υ rays, with a total emitted energy of 28 MeV. The α particles contribute the most (95.3%) to this quantity and allow the deposition of a relevant absorbed dose. The decay scheme of Radium-223, including the closest radionuclide parents, is reported in Figure 10.

The six-stage-decay of Radium-223 involving radon-219 (half-life of 4 s), polonium-215 (half-life of 1.8 ms), lead-211(half-life of 36 min), bismuth-211 (half-life of 2.2 min) and thalium-207 (half-life of 4.8 min) leads to stable lead-207 and occurs via short-lived daughters, and is accompanied by several alpha, beta and gamma emissions with different energies and emission probabilities. The fraction of energy emitted from Radium-223 and its daughters as alpha-particles is 95.3% (energy range of 5.0–7.5 MeV). The fraction emitted as beta-particles is 3.6% (average energies are 0.445 MeV and 0.492 MeV), and the fraction emitted as gamma-radiation is 1.1% (energy range of 0.01–1.27 MeV).

#### 2.6.2. Radiochemistry

Radium is an alkaline earth metal, mainly found in the +2 oxidation state, with chemical properties similar to magnesium, barium, and calcium [12,136]. With a [Rn] 7s2 electronic configuration, the corresponding divalent cation [^223^Ra]Ra^2+^ is the only species formed. Concerning its potential chelation as a hard acceptor, a more pronounced affinity to complex donor atoms such as oxygen is expected [11]. Different compounds (DTPA, kryptofix 2.2.2, calix [4]-tetraacetic acid, DOTA) were tested as chelating agents for [^223^Ra]Ra^2+^ ion, but they all resulted in extremely unstable [134,137] and unsuitable for in vivo studies. However, Whilson and Thorec, in this work, showed that macropa, an 18-membered bis-picolinate diazacrown macrocycle, is an effective chelator of [^223^Ra]Ra^2+^ demonstrating rapid complexation kinetics and profound in vivo stability. The authors also investigated [^223^Ra]Ra^2+^ chelation utilising a bifunctional derivative of macropa conjugated to a single amino acid, β-alanine, or a prostate cancer-targeting agent, DUPA [135]. The possibility of radiolabelling through encapsulation in biomaterials or nanomolecules [138] was also explored, using PEGylated liposomal doxorubicin, lanthanum phosphate nanoparticles (LaPO_4_), iron oxide nanoparticles (Fe_2_O_3_) and nanozeolite NaA [136,137], barium sulphate (BaSO_4_) [139,140], barium ferrite nanoparticles (BaFeNPs) [141], titanium dioxide (TiO_2_) [142] and hydroxyapatite. Those solid-state nanoparticles stabilise [^223^Ra]Ra^2+^ and alter their biodistribution properties. However, this scenario must be further investigated and tested in clinical studies. Ivanow and colleagues from Oak Ridge National Laboratory (ORNL) in Tennessee, US, used quantum chemical calculations that allow them to peer inside radium to see its electronic structure. They also examined how the ligand molecule orbitals overlap with vacant orbitals on radium. As a result, they found that the bonding is ionic and that electrostatic attraction plays a huge role [143]. Currently, Radium-223 is mainly used in its chloride salt form [^223^Ra]RaCl_2_, which naturally targets hydroxyapatite and bone matrix.

#### 2.6.3. Preclinical Studies

Preclinical studies with radium-223 demonstrated its capability of targeting bone surfaces, releasing high-absorbed doses to neoplastic areas while sparing nearby healthy tissues. Radium-223 mimics calcium and selectively targets bone, specifically areas of bone metastases, by forming complexes with the bone mineral hydroxyapatite. Effects on the tumour microenvironment including osteoblasts and osteoclasts also contribute to the in vivo efficacy. List of relevant preclinical studies involving Radium-223 is shown in Table S3 (Supplemental Data).

#### 2.6.4. Clinical Studies

Clinical studies in this context focused on the use [^223^Ra]RaCl_2_ for the treatment of mCRPC, especially in cases of chemotherapy or hormone therapy resistance. To date, the Phase III ALpharadin in SYMptomatic Prostate CAncer patients (ALSYMPCA) study is the trial with the largest cohort (n = 921) of patients for the evaluation of Radium-223 antitumoral effect and survival analysis in cases of mCRPC. Patients in the Radium-223 arm, receiving six radiopharmaceutical injections at 55 kBq/kg every 4 weeks, showed longer overall survival and a longer time to a first skeletal event than those in the placebo arm [144,145]. Following the ALSYMPCA study, [^223^Ra]RaCl_2_ was validated by the FDA and EMA in 2013 for the treatment of bone metastases in mCRPC cases and became the first radiopharmaceutical approved for TAT. Evaluation of all secondary efficacy endpoints and myelosuppression also benefited the Radium-223-treated patients [145]. A large, randomised phase 3 trial (ERA 223) also assessed the combination of Radium-223 with abiraterone acetate plus prednisone or prednisolone: this combined therapy was associated with an increased frequency of bone fractures, so it was not recommended in mCRPC [146]. Based on the results of the ERA study, the EMA Pharmacovigilance Risk Assessment Committee (PRAC) provided a benefit/risk review for Radium-223, which ended with a restriction of therapeutic indications (EPAR 11 October 2018) and confirmation of temporary contraindication measures. Due to the increased risk of fractures and possible increased mortality observed with the combination of Radium-223 with abiraterone acetate and prednisone/prednisolone, this triple combination remains contraindicated. Furthermore, the initiation of Radium-223 treatment is not recommended in the first 5 days following the last dose of abiraterone and prednisone/prednisolone. Subsequent systemic anticancer treatment should not be started for at least 30 days after the previous dose of Radium-223. Several combinations are currently studied in phase I or phase II trials, especially with enzalutamide (an androgen receptor signalling inhibitor), pembrolizumab (a monoclonal antibody against PD1 protein), niraparib or olaparib (both are inhibitors of poly-ADP-ribose polymerase). Even if advanced prostate cancer is the primary pathology targeted with Radium-223, this radionuclide is under investigation in other pathologies associated with bone metastases, such as breast or renal cancer. The work on clinical applications with Radium-223 is considerable, and detailed reviews can provide more information about the global state of the art on Radium-223 and ongoing clinical trials [11].

List of clinical studies involving Radium-223 reported in database ClinicalTrials.gov is shown in Table S4 (Supplemental Data).

#### 2.6.5. Conclusions

The use of Radium-223 for treating bone metastases from mCRPC is largely diffused and standardised due to the FDA and EMA approval of [^223^Ra]RaCl_2_ in 2013. However, several combinations with monoclonal antibodies or different inhibitors are being studied, and the possibility of treating skeletal metastases from other primary tumours is being evaluated. Another important challenge in expanding the clinical use of Radium-223 for treating tumours is an effort to develop new chelators forming Radium-223 forming in-vivo stable complexes. So far, only macropa seems to be the most effective, even with some limitations.

### 2.7. Thorium-227

#### 2.7.1. Physical Characteristics

**Figure 11:**
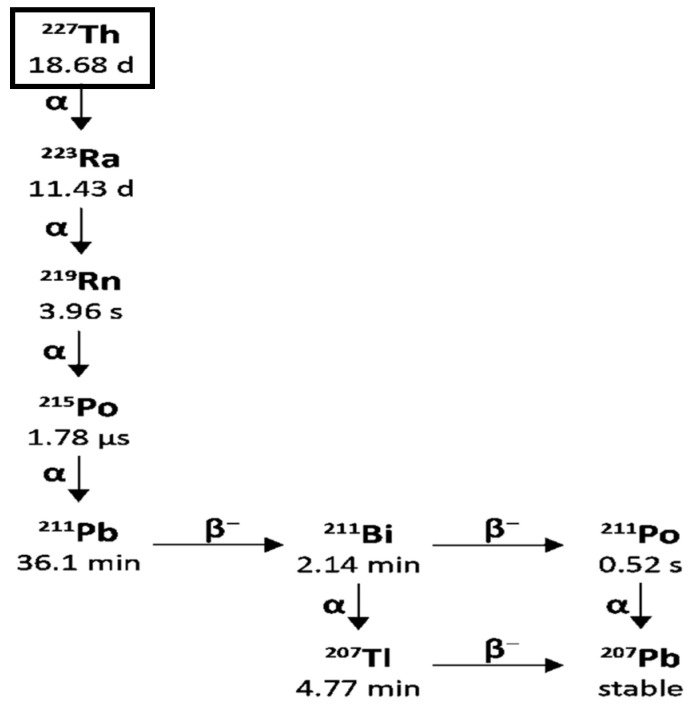
Decay chain of Thorium-227.

Thorium-227 is an alpha-emitting radionuclide with a physical half-life of 18.7 days. It is part of the actinium series, and it decays into Radium-223, releasing a 5.7 MeV (average energy) alpha particle. Four gamma emissions in the 200–350 keV energy range are associated with the decay, 236 keV (13%) being the most abundant. The latter can be used for imaging purposes, along with the 269 keV peak of the daughter radionuclide Radium-223 [136]. A summary of the decay of Thorium-227 is reported in Figure 11.

#### 2.7.2. Radiochemistry

Thorium-227 can have multiple oxidation states, but in the aqueous medium, the most stable one is +4. Free thorium showed high targeting capability for hydroxyapatite, a mineral form of calcium apatite largely present in vertebral bones. Still, its use has been discouraged considering its tendency to also accumulate in the kidneys [11]. Therefore, chelation with phosphonate derivatives, which have a high affinity for bones, has been tested. More precisely, Thorium-227-complexes with DTMP, DOTMP and EDTMP demonstrated high and selective bone uptake and chemical stability [11,147]. The well-known DOTA also resulted in a suitable solution for Thorium-227 chelation, although high temperature and a two-step procedure were necessary. Different synthetic analogues were developed to overcome these limitations, the most promising one being hydroxypyridinone moiety (HOPO). Polydentate HOPO ligands are stable and exhibit low in vivo toxicity [11].

#### 2.7.3. Preclinical Studies

Thorium-227 complexes evaluated in preclinical studies used monoclonal antibodies (rituximab, trastuzumab) and biomarkers (CD33, CD70) as targeting agents. [^227^Th]Th–DOTA–Rituximab was tested on Non-Hodgkin lymphoma and CD20-positive lymphoma cell lines and a delay in cellular growth was observed. [^227^Th]Th–trastuzumab has also been used. A list of relevant preclinical studies involving Thorium-227 is shown in Table S3 (Supplemental Data).

#### 2.7.4. Clinical Studies

The literature of clinical trials involving Thorium-227-labelled agents is still limited, even though four phase I trials, registered in ClinicalTrials.gov, are now completed:

- BAY2287411 (or MSLN-TTC) for solid tumours expressing mesothelin (NCT03507452).
- BAY2701439 (or HER2-TTC) for cancers with HER2 expression as breast cancer or gastric cancer (NCT04147819).
- BAY2315497 (or PSMA-TTC) for mCRPC (NCT03724747). Intermediate results from different studies have already been reported.
- BAY 1862864, which is a [^227^Th]Th-labelled CD22-targeting antibody, was injected into patients with CD22-positive relapsed/refractory B cell non-Hodgkin lymphoma (R/R-NHL) (NCT02581878), and the therapy resulted in safe and well-tolerated, with an objective response rate of 25%.

List of relevant clinical studies involving Thorium-227 is shown in Table S4 (Supplemental Data).

#### 2.7.5. Conclusions

The literature on clinical trials involving Thorium-227-labelled agents is still scarce, but optimal results were observed in preclinical studies with delays in cellular growth, multiple double-strand breaks, and complete regression. Intermediate phase I trial results are also reported, and safety, tolerability, and an objective response rate of 25% are shown.

## 3. Materials and Methods

Alpha emitters identified as clinical key players in the 2022 PRISMAP report, which surveyed European facilities and research institutions, were selected [148]. Moreover, a secondary search in the literature and Symposium articles focused on the most promising alpha emitters in molecular radiation therapy confirmed this selection [11]. The decay schemes were reproduced from the same source: https://epa-prgs.ornl.gov/radionuclides/chain/chain.php (accessed on 3 August 2025). We conducted a literature search of the most important databases, including the selected radionuclides (some examples: European Medicines Agency (EMA) database, Medline, PubMed, Embase, Scopus, Clinical Trials.gov).

Regarding criteria applied for literature search, to find the potentially relevant articles, the following keywords were used: “X-n” OR “alpha-emitter” OR “radionuclide therapy” OR “targeted alpha particle therapy” OR “radiolabelled therapy” OR “peptide receptor radionuclide therapy” AND “preclinical studies” AND “clinical studies” AND “physics characteristics” AND “radiochemistry. Only articles in the English language were included.

Key details on the SPECT settings for the image acquisitions, the time points at which the acquisitions are obtained for dosimetry purposes, when the information is available in the literature, are shown in Table S5 (Supplemental Data).

## 4. Discussion, Recommendations and Conclusions

Alpha-emitting radionuclides show great promise in clinical trials, highlighting their potential to provide substantial patient benefit. However, the advancement of such therapies relies on overcoming several critical challenges. Sustained access to high-quality and scalable radionuclide supplies is essential, as the availability of suitable isotopes often dictates the feasibility and scope of clinical trials as much as the intrinsic chemical or physical properties of the radionuclides themselves. Production is further complicated by restrictions imposed by half-lives, isotopic purity, and complex radiochemistry. Promising approaches to address these limitations include the development of novel chelating agents and precursors for radiopharmaceuticals, as well as the exploration of alternative production methods, such as accelerator-based routes.

Another important consideration is that many alpha-emitting radionuclides decay into radioactive progeny rather than stable elements. The recoil energy generated during alpha decay can release daughter nuclides from the targeting moiety both during transport and after administration, creating potential off-target effects with implications for dosimetry, radiation safety, and therapeutic efficacy. The design of clinical trials must therefore account for these challenges, including careful integration of dosimetry and the optimisation of chelating agents to minimise dissociation of the “free” radionuclide. Standardised dosimetry methodologies and harmonised reporting of trial outcomes would help ensure comparability and reproducibility across studies, thereby accelerating clinical translation.

Although early results are promising, limited clinical trial data prevent recommending specific alpha-emitting radionuclides for certain diseases or stages. Ongoing and future studies should therefore be supported not only to validate therapeutic efficacy but also to enable reverse translation and improve our understanding of the underlying radiobiology. Within this context, the seven isotopes reviewed in this work illustrate the multidisciplinary nature of this field, combining nuclear physics, radiochemistry, oncology, and dosimetry to optimise therapeutic applications.

Dosimetry in targeted alpha therapy (TAT) presents significant challenges due to the unique characteristics of alpha-emitting radionuclides and their short-range, high-linear energy transfer emissions. Unlike beta emitters, for which dosimetry can often rely on macroscopic dose calculations, alpha particles deposit their energy within a few cell diameters. This necessitates a more granular approach to dosimetry. A major challenge is the difficulty of accurately quantifying the microscopic distribution of alpha-emitting radiopharmaceuticals within tissues and at a cellular level. Current imaging techniques, such as SPECT and PET, provide information on macroscopic biodistribution, but lack the spatial resolution required to visualise the precise localisation of individual alpha-emitting atoms within tumour cells or critical normal tissues. For instance, when a therapeutic agent is designed to target specific receptors on cancer cells, the actual dose delivered to each cell depends on the number of internalised radionuclides and how they are distributed inside the cell, which cannot be observed directly using current clinical imaging techniques. This limitation directly affects patient-level dosimetry in clinical trials, as the calculated macroscopic absorbed dose may not accurately reflect the true dose delivered to individual cells. This can lead to underestimation of toxicity to healthy cells or suboptimal therapeutic efficacy in tumour cells. Another challenge lies in the complex biological effects of high-LET radiation. The radiobiological effectiveness (RBE) of alpha particles can vary significantly depending on factors such as the dose rate, the type of cell and the oxygenation status. Standard dosimetry models often use a fixed RBE value, which may not accurately reflect the actual biological impact in all clinical scenarios. This uncertainty in RBE complicates the translation of absorbed dose to biological effect, limiting the ability to predict therapeutic outcomes or adverse events with precision.

Overall, the therapeutic potential of alpha-emitting radionuclides lies in their ability to deliver high linear energy transfer over short ranges, effectively targeting and destroying cancer cells while sparing surrounding healthy tissues. This is particularly valuable in refractory or metastatic cancers where conventional therapies are insufficient. Nevertheless, progress in clinical translation will depend on optimising production processes, ensuring isotope stability and safety, and addressing the challenges of complex decay schemes and limited imaging capabilities. By integrating advances in isotope production, radiochemistry, dosimetry, and clinical trial design, alpha-emitting radionuclide therapies may evolve from promising preclinical findings to scalable and effective cancer treatments.

## Data Availability

No new data were created or analyzed in this study. Data sharing is not applicable to this article.

## Supplementary Materials

The following supporting information can be downloaded at: https://www.mdpi.com/article/10.3390/ph18101578/s1, References [28,49,58,60,92,98,103,111,122,129,149,150,151,152,153,154,155,156,157,158,159,160,161,162,163,164,165,166,167,168,169,170,171,172,173,174,175,176,177,178,179,180,181,182,183,184,185,186,187,188,189,190,191,192,193,194,195,196,197,198,199,200,201,202,203,204,205,206,207,208,209,210,211,212,213,214,215,216,217,218,219,220,221,222,223,224,225,226,227,228,229,230,231,232,233,234,235,236] are cited in the supplementary materials. Table S1. Comparative table of Key Differences alpha (α) vs beta (β) emitters and physical characteristics and chelators of the a-radionuclide. Table S2. Tested Actinium-225 chelates coupled with targeting vectors for in vitro or in vivo application and their radiolabelling yields (RCY) and stabilities. Table S3. List of relevant preclinical studies. Table S4: Overview of some of the current/ongoing clinical studies registered in ClinicalTrials.gov. Table S5. Key details on the SPECT settings for the image acquisitions, the time points at which the acquisitions are obtained for dosimetry purposes.
