# Supplementary material for: Recommendations on the Clinical Application and Future Potential of α-Particle Therapy: A Comprehensive Review of the Results from the SECURE Project
Source: Pharmaceuticals (Basel). 2025 Oct 18;18(10):1578. doi: 10.3390/ph18101578 (PMC12567000; doi:10.3390/ph18101578)
Supplement: Supplementary file 1 [file pharmaceuticals-18-01578-s001.zip › pharmaceuticals-3837230-supplementary.pdf]

Supplemental Data

Table S1. Comparative table of Key Differences alpha (a) vs beta (b) emitters and physical characteristics and chelators of the a-radionuclide.

Feature	a Emitters	b Emitters
**Energy & Range**	High energy, very short range	Moderate energy, longer range
**DNA Damage**	Dense ionization, clustered double-strand breaks	Widely spaced ionization, single-strand breaks
**Targeting**	Highly selective for small, homogenous tumors	Broad reach, beneficial for large or heterogeneous tumors
**Toxicity**	Low off-target toxicity, but potential for daughter escape	Higher risk of off-target toxicity due to longer range
**Resistance**	Less dependent on tumor oxygenation	Efficacy can be dependent on oxygenation

Radionuclide	Half-life	Decay types	a-emission energies (MeV)	Tissue range (mm)	Imaging feasibility (D=daughters)	Chelators
**Actinium-225**	9.9 d	4α, 2β	5.8-8.4	70	D (Fr-221, Bi-213)	DOTA, H_2-macropa, Crown
**Bismuth-213**	46 min	1α, 2β	5.8-8.4	40-100	γ = 440.45 keV (25.94%)<br>β^- = 24,6-492,2 KeV (97.8%)<br>D (Tl-209)	DOTA, DOTP, DTPA, Pyrydine-aza-crown ethers
**Astatine-211**	7.2 h	1α, 1 EC	5.9 or 7.4	60	x-ray = 76.86 keV (12.4%)<br>79.29 keV (20.7%)	Covalent bond
**Lead-212/Bi-212**	10.6 h	1α, 2β	6,1-7.8	600	238.6 keV (43,6%), 300.1 keV<br>Bi-212 γ = 727.33 keV (6.67%)<br>β^- = 130.1–834.2 keV (64.06%)	DOTA, TCMC, DOTAM, PSC
**Terbium-149**	4.1 h	1α, 1 EC	3.9	3.9	γ = 164.98 keV (26.4%)<br>352.24 keV (29.4%)<br>388.57 keV (18.4%)<br>464.85 keV (5.65%)<br>652.12 keV (16.2%)<br>817.1 keV (11.6%)<br>853.43 keV (15.5%)<br>861.86 keV (7.5%)<br>β^+ = 200.1–1107.2 keV (7.1%)	DOTA, CHX-A′′-DTPA
**Radium-223**	11.4 d	4α, 2β	5.7-7.4	60	γ = 154.208 keV (5.7%)<br>269.463 keV (13.9%)<br>x-ray = 81.069 keV (15%)<br>83.787 keV (24.7%)	Coordination chemistry very limited, macropa
**Thorium-227**	18.7 d	5α, 2β	5.7-7.4	50-80	γ = 154.208 keV (5.7%)<br>269.463 keV (13.9%)<br>x-ray = 81.069 keV (15%)<br>83.787 keV (24.7%)<br>D (Radium-223,Bi-211)	DTMP, DOTMP, EDTMP, HOPO

Table S2. Tested Actinium-225 chelates coupled with targeting vectors for in vitro or in vivo application and their radiolabelling yields (RCY) and stabilities.

Chelate (and corresponding tested bifunctional analogues	Donor Set (CN#)	Grade	Radiolabelling Conditions & RCY	Ref.
DOTA 1,4,7,10-tetraazacyclododecane-1,4,7,10-tetraacetic acid 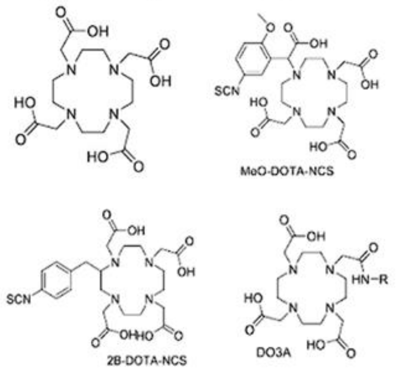	**N_4O_4 CN = 8**	Green -orange	0.02 M ligand, NH_4Ac pH 6, 37 °C, 2 h, RCY = 99%	[1]
DOTPA 1,4,7,10-tetraazacyclododecane-1,4,7,10-tetrapropionic acid 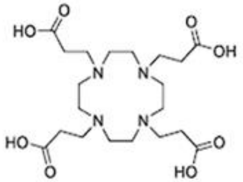	**N_4O_4 CN = 8**	Red	0.02 M ligand, NH_4Ac pH 6, 37 °C, 2 h, RCY = 0%	[2]
DOTMP 1,4,7,10-tetraazacyclododecane-1,4,7,10-tetramethylene-phosphinic acid 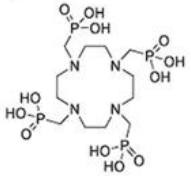	**N_4O_4 CN = 8**	Red	0.02 M ligand, NH_4Ac pH 6, 37 °C, 2 h, RCY = 78%	[2]
TETPA 1,4,8,11-tetraazacyclotetradecane-1,4,8,11-tetrapropionic acid 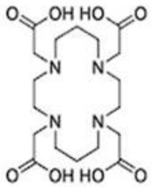	**N_4O_4 CN = 8**	Red	0.02 M ligand, NH_4Ac pH 6, 37 °C, 2 h, RCY = 0%	[2]

DTPA diethylenetriaminepentaacetic acid<br>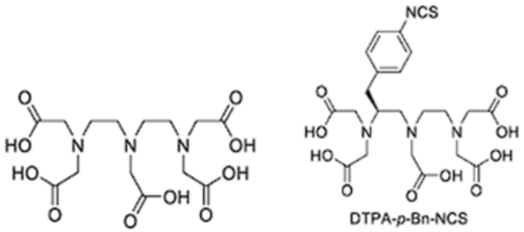	**N_3O_5**<br>**CN = 8**	Red	0.02 M ligand,<br>NH_4Ac pH 6,<br>37 °C,<br>2 h,<br>RCY = 0%	[2]
PEPA 1,4,7,10,13-pentaazacyclopentadecane-*N,N',N'',N''',N''''*-pentaacetic acid<br>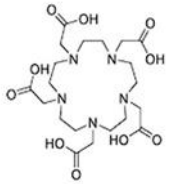	**N_5O_5**<br>**CN = 10**	Red	0.02 M ligand,<br>NH_4OAc pH 5.8,<br>40 °C,<br>30 min,<br>RCY = 80%	[3]
HEHA 1,4,7,10,13,16-hexaazacyclohexadecane-N,N',N'',N''',N'''',N'''''-hexaacetic acid<br>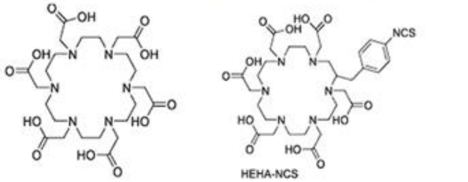	**N_6O_6**<br>**CN = 12**	Orange	0.01 M ligand, NH_4OAc pH 5.8, 40 °C, 30 min, RCY > 95% or > 98% after 2 h	[3,4]
CHX-A''-DTPA<br>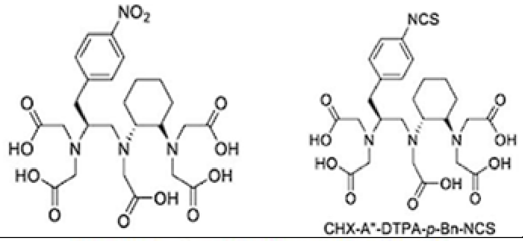	**N_3O_5**<br>**CN = 8**	Red	0.01 M ligand,<br>0.02 NH_4OAc pH 5.8,<br>0.03 40 °C,<br>0.04 30 min,<br>0.05 RCY ><br>95%	[3,5]
EDTA ethylenediaminetetraacetic acid<br>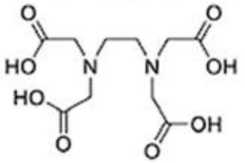<br>EDTA	**N_2O_4**<br>**CN = 6**	Red	0.01 M ligand,<br>0.02 NH_4OAc pH 5,<br>0.03 40 °C, 30 min,<br>0.04 RCY = 80-90%	[5]

Table S3. List of relevant preclinical studies.

	Preclinical model	Radiopharmaceutical	Activity/no of cycles	Main findings	Ref.
Actinium-225	AR42J cells	[^{225}Ac]Ac -DOTA-CCK-66	37 kBq / 1 cycle	Substantial increase in mean survival of AR42J tumour-bearing mice upon treatment with the minigastrin derivative	[6]
Actinium-225	Human ovarian carcinoma HER2-positive(SKOV-3 cell line)	[^{225}Ac]Ac -H_4py4pa	10.1±0.7 kBq / 1 cycle	Stability study, in vitro and biodistribution	[7]
Actinium-225	Human breast cancer cell lines SUM-225 and MDA-MB-231	[^{225}Ac]Ac -DOTA-trastuzumab	0.37 kBq / 0.74 Bq / 1.48 Bq / 1 cycle	In vitro, biodistribution (comp. with In-111-DTPA-trastuzumab), optical imaging and therapy study.	[8]
Actinium-225	Human HER2- positive cell lines SKOV-3 (ovarian cancer) and MDA-MB-231 (breast cancer)	[^{225}Ac]Ac- DOTA-Nb (nanobod)	30.2 ± 1.4 kBq / 1 cycle	In vitro and biodistribution In vitro, therapy study, dosimetry and toxicity	[9]
Actinium-225	Human HER2- positive cell lines SKOV-3 (ovarian cancer) and MDA-MB-231 (breast cancer)	[^{225}Ac]Ac-DOTA-Nb (nanobod)	81.67 ± 28.87 kBq / 3 cycles	In vitro and biodistribution In vitro, therapy study, dosimetry and toxicity	[10]
Actinium-225	U87mg human glioblastoma tumour cells	[^{225}Ac]Ac-DOTA-c(RGDyK)	10 kBq / 20 kBq / 40 kBq/ 1 cycle	Biodistribution, optical imaging and therapy study	[11]
Actinium-225	Human glioblastoma cell line U251	[^{225}Ac]Ac-Pep-1L	40 kBq / 1 cycle	Bioluminescent imaging, therapy study (comparison with Cu-64-PepL1)	[12]
Actinium-225	NT2.5 mammary tumour cell line	[^{225}Ac]Ac-DOTA-anti-PD-L1-BC	15 KBq / 1 cycle	Biodistribution (comparison with In-111-DTPA anti-PD-L1-BC), imaging, dosimetry	[13]
Actinium-225	Human prostatic carcinoma cells LNCaP	[^{225}Ac][Ac-(macropa)]$^{+}$	26 kBq / 1 cycle	In vitro and biodistribution	[14]
Actinium-225		[^{225}Ac][Ac-(macropa)]$^{+}$	37 KBq / 74 KBq / 148 KBq / 1 cycle	In vitro, biodistribution, therapy study and dosimetry	[15]
Actinium-225	Human pancreatic cell line BxPC3	[^{225}Ac]Ac-DOTA Human antibody 5B1	18.5 kBq / 1 cycle	Biodistribution, luminescence imaging, therapy studies (pre-targeting or conventional) and toxicity	[16,17]
Actinium-225	Mammary carcinoma cell lines MFM-223 and BT-474	[^{225}Ac]Ac-hu11B6H435A	11.1 kBq / 1 cycle	In vitro, biodistribution and therapeutic study	[18]
Actinium-225	Triple-negative breast cancer model SUM149T	[^{225}Ac]Ac-DOTA-cixutumumab	8.32 kBq / 1 cycle	In vitro, imaging, biodistribution (comp.with In-111-cixutumumab) and efficacy study	[19]
Actinium-225	Malignant melanoma cell line B16F10	[^{225}Ac][Ac-octapa]$^{-}$[^{225}Ac][At-(CHX-octapa)]$^{-}$; [^{225}Ac][Ac(DOTA-CycMSH)]	12–20 kBq / 1 cycle	Stability study and biodistribution	[20]
Actinium-225	Human cutaneous melanoma cells A375 andA375/MC1R and human uveal melanoma cells MEL270	[^{225}Ac]Ac-DOTA-MC1RL	148 kBq (±10%) / 1 cycle	In vitro, pharmacokinetic, biodistribution, therapy study and dosimetry	[21]

Actinium-225	Human cutaneous melanoma cells A375 and A375/MC1R	[^{225}Ac]Ac-DOTA-Ahx-MC1RL (^{225}Ac-Ahx); [^{225}Ac]Ac-DOTA-di-d-Glu-MC1RL (^{225}Ac-di-d-Glu)	94.84 kBq ±7.11% / 56.52 kBq ±8.2 / 1 cycle	Biodistribution, pharmacokinetics, therapy study and toxicity	[22]
Actinium-225	Malignant melanoma cell line B16F10	[^{225}Ac]Ac-DOTA-Anti-VLA-4	14.8 kBq / 1 cycle	In vitro, biodistribution, imaging dosimetry and therapeutic efficacy	[23]
Actinium-225	Human embryonic kidney epithelial cells HEK-293T and HEK-293T-Hx16	[^{225}Ac]Ac-DOTA- SC16.56 (radioimmunoconjugate- Humanised site-specific antibodiesN149)	18.9 – 55.5 kBq / 1 cycle	In vitro, biodistribution and efficacy study (comparison with Lu-177-DOTA-MMA)	[24]
Actinium-225	Colorectal cancer (SW1222), breast cancer (BT-474) or neuroblastoma (IMR32)	[^{225}Ac]Ac-Proteus-DOTA (Humanised A33 and C825 (huA33-C825)	0, 9.25, 18.5, 37, 74, 148, or 296 kBq / 1 cycle	Biodistribution (comparison with ^{111}In-Pr, imaging) therapy study and toxicity(Pretargeted radioimmunotherapy)	[25]
Actinium-225	Human pancreatic cell lines PANC-1 and MIA PaCa-2	[^{225}Ac]Ac-FAPI-04	34 kBq / 1 cycle	In vitro, biodistribution and efficacy study	[26]
Actinium-225	Human squamous carcinoma A431 cell line	[^{225}Ac]Ac-DOTA-PP-F11N	45 kBq or 60 kBq / 1 cycle	In vitro, biodistribution and therapy study	[27]
Actinium-225	Hepatoblastoma cell line HepG2 and squamous carcinoma A431 (GPC3+)	[^{225}Ac]Ac-Macropa-GC33	9.25 kBq or 18.5 kBq / 1 cycle	In vitro, biodistribution, therapy study and toxicity	[28]
Bismuth-213	Multiple myeloma	[^{213}Bi]Bi-anti CD138	3.7 MBq (single dose)	Increased median survival to 80 days, compared with 37 days for the untreated control group	[29,30]
Bismuth-213	Bladder carcinoma	[^{213}Bi]Bi-anti-EGFR-mAb	0.94 MBq (fractioned dose)	Overall survival of 141.5 days on average, in contrast with 65.4 and 57.6 days for the two control groups	[31]
Bismuth-213	AR42J tumour- bearing mice; H69 human small-cell lung carcinoma; CA20948 rat pancreatic tumour	[^{213}Bi]Bi-DOTATATE	2–4 MBq/0.3 nmol/ 200 µL	Significant tumour burden reduction and improved overall survival	[32,33]
Astatine-211	syngeneic immunocompetent rat model	[^{211}At]At-BR96	2.5 or 5 MBq	Possibility of treating small, solid colon carcinoma tumours with tolerable toxicity	[34,35]
Astatine-211	U87MG cells<br>Nude mice bearing xenograft tumours	[^{211}At]At-iRGD- C6-lys-C6-DA7R	180, 370 and 740 kBq	Inhibition of cell viability, induced cell apoptosis, arrested the cell cycle in the G2/M phase, and increased intracellular ROS levels in a dose-dependent manner; inhibition of tumour growth and prolongation of the survival of mice	[36]
Astatine-211	T98G glioma cell line	[^{211}At]At-Rh[16aneS4]- SP5–11	75–1200 kBq/mL	Cytotoxic effect on glioma cells	[37,32]
Astatine-211	DBTRG-05MG glioma cell line, female BDIX rats with intracranial glioblastomas	2-[^{211}At]At-Phenylalanine<br>4- [^{211}At]At-Phenylalanine	1000 kBq (1 or 2 cycles)	Enhanced survival time of rats with intracranial glioblastomas	[38,39]
Astatine-211	Athymic mice bearing subcutaneous D-54 MG human glioma xenografts	[^{211}At]At-ch81C6	74 kBq	Calculation of human radiation dose for i.v. and intrathecal administration	[40]
Astatine-211	HNSCC-Bearing female nude mice (balb/c nu/nu)	[^{211}At]At-U36 (Chimeric mAb)	200 kBq	Specific binding to the glycoprotein and efficient therapeutic response	[41]

Astatine-211	HL-60 and CI-1 cells	[^{211}At]At -rituximab; [^{211}At]At -gemtuzumab; [^{211}At]At gemtuzumab ozogamicin.	0.03 to 9.29 kBq (to 106 cells)	The affinity and specificity of the respective epitopes are not compromised	[42]
Astatine-211	leukemic SJL/J mice	[^{211}At]At-30F11 (anti-murine CD45; mAb)	444, 740 and 888 kBq	Improvements in overall survival when combined with bone marrow transplantation in a disseminated model of murine leukaemia with minimal renal toxicity	[43]
Astatine-211	Female BALB/c mice	[^{211}At]At-30F11- ADTM	74, 370, 740 and 1850 kBq	more effective at myelosuppression than Bismuth-213, no significant non hematopoietic toxicity	[44]
Astatine-211	Human ML xenograft model in male hymic BALB/c nude mice	[^{211}At]At -CXCR4 (mAb)	320 kBq	clearance from blood and the tumour uptake matched the physical half-life of Astatine-211; tumour uptake was relatively low	[45]
Astatine-211	Female and male NOD-Rag1null IL2rɣnull/J (NRG) mice	[^{211}At]At-B10 (conjugated anti-CD123; mAb)	185, 370, 740 or 1480 kBq	decreased tumour burden and significantly prolonged dose-dependent survival	[46]
Astatine-211	Female athymic nude mice (s.c. injected Ramos cells)	[^{211}At]At-1F5- B10	Up to 1776 kBq	highly efficacious in minimal residual disease, no significant renal or hepatic toxicity	[47]
Astatine-211	Normal Kunming (KM) mice, BALB/c nude mice (s.c. injected A549 cells)	[^{211}At]At -SPC-octreotide	2294 kBq	more lethal effect than control groups (PBS, octreotide and free ^{211}At), a possible treatment option for NSCLC	[48]
Astatine-211	Human melanoma- xenografted nude mice	[^{211}At]At-MTB (methylene blue)	3,5 MBq	highly effective, no adverse effects of TAT	[49]
Astatine-211	Female and male NOD.Cg Rag1tm1Mom Il2rgtm1Wjl/SzJ (NRG) mice	[^{211}At]At-OKT10- B10	277 to 1665 kBq	potential to eliminate residual MM cell clones in low-disease-burden settings with minimal toxicity	[50]
Astatine-211	KaLwRij C57/BL6 mice (i.v. injected 5T33 cells)	[^{211}At]At-9E7.4	370, 555, 740 or 1110 kBq	the activity of 740 kBq showed 65% overall survival 150 days after the treatment with no evident sign of toxicity in MDR of multiple myeloma.	[51]
Astatine-211	NB-EBC1x tumour- bearing mouse model (female SCID CB17 mice)	[^{211}At]At-parthanatine (PTT)	185 kBq	maximum tolerated dose (MTD 36 MBq/kg/fraction x4), complete tumour response was observed in 81.8% with reversible haematological and marrow toxicity	[52]
Astatine-211	Male ICR mice (6 weeks old)	[^{211}At]At-MABG (astatobenzylguanidine)	185 kBq (biodistribution) 1.1, 2.2, 3.3, 4.4 MBq (body weigth studies)	the MTD was 3.3 MBq for ICR mice.	[53,54]
Astatine-211	female BALB/c nude mice s.c. inoculated with NIH: OVCAR-3 cells	[^{211}At]At-farletuzumab	700 kBq	the tumour-free fraction (TFF) was shown to be 91% for i.p. administered 211At-farletuzumab	[55]
Astatine-211	nude Balb/c nu/nu mice (i.p. inoculated with OVCAR-3 cells)	[^{211}At]At-MX35 (mAb)	800 kBq and 3× ~267 kBq ~400 kBq and 3× ~133 kBq ~50 kBq or 3× ~17 kBq	no advantage in the therapeutic efficacy of a fractionated regimen compared with a single administration and lower side effects	[56]
Astatine-211	nude Balb/c nu/nu mice (i.p. inoculated with OVCAR-3 cells)	[^{211}At]At-MX35 (mAb)	350 - 540 kBq	micrometastatic growth of an ovarian cancer cell line was reduced with no considerable signs of toxicity	[57]

Astatine-211	nude Balb/c nu/nu mice (i.p. inoculated with SKOV-3 cells)	[^{211}At]At-trastuzumab (mAb)	100 – 800 kBq	statistically significant dose-response relationship for a single i.p. injection, a combination of 500 µg trastuzumab and 400 kBq ^{211}At-trastuzumab had the greatest effect	[58]
Astatine-211	s.c. and PMGC (peritoneal metastasis of gastric cancer) xenograft mice	[^{211}At]At-trastuzumab (mAb)	100 and 1000 kBq	locoregionally administered [^{211}At]At-trastuzumab significantly prolonged the survival time	[59]
Astatine-211	Female nude BALB/c (nu/nu) mice (s.c. inoculated with SKOV-3 cells)	N-succinimidyl- 3-[^{211}At]At-5-guanidinomethyl benzoate	700 kBq	fast and high accumulation in a HER2+ tumour mouse model with a low non- target organ uptake	[60]
Astatine-211	female athymic mice (s.c. inoculation of 9BT474 xenografts)	Iso-[^{211}At]At SAGMB-5F7<br>Iso-[^{211}At]At SAGMB- VHH_2001	130 - 175 kBq	significant tumour growth delay and survival prolongation in a murine model of HER2-expressing breast cancer with no apparent normal- tissue toxicities	[61]
Astatine-211	C.B17/Icr-scid mice (s.c. implantation of MDA-361/DYT2 cells)	[^{211}At]At-SAPS C6.5 (diabody);<br>[^{211}At]At-SAPS T84.66 (diabody;<br>[^{211}At]At-SAPS (anti-MISIIR GM17 diabody)	740, 1110 or 1665 kBq	single i.v. treatment resulted in dose- dependent delays in tumour growth	[62]
Astatine-211	Athymicmice bearing PSMA+ PC3, PIP and PSMA-<br>PC3 flu flank xenografts	(2S)-2-(3-(1-carboxy-5-(4-[^{211}At]At astatobenzamido)pentyl)ureido)-pentanedioic acid	200 kBq, 740 kBq	specific PC cell kill in vitro and in vivo after systemic administration and late nephrotoxicity	[63]
Astatine-211	LNCaP xenograft mice, normal ICR mice	[^{211}At]At-PSMA1;<br>[^{211}At]At-PSMA5;<br>[^{211}At]At-PSMA6	110 – 400 kBq	[^{211}At]At-PSMA5 exhibited excellent tumour growth suppression in xenograft models of prostate cancer, with minimal side effects.	[64]
Astatine-211	Male nude BALB/c nu/nu mice (s.c. inoculated with PC3- PSCA tumour cells)	[^{211}At]At-A11 (anti-PSCA mini body)	260 ± 20 kBq,<br>800 kBq and 1500 kBq	growth inhibition on both macro tumours and intratibial micro tumours and multiple fractions resulted in radiotoxicity	[65]
Astatine-211	Male nude BALB/c nu/nu mice (s.c. inoculated with PC-3 cells)	[^{211}At]At-AB-3	85 kBq	poor in vivo stability	[66]
Astatine-211	NIS-6 cells	[^{211}At]At-astatide	50-100 kBq	uptake is shown to be NIS-dependent	[67,68]
Astatine-211	NMRI-nu/nu nude mice (s.c. inoculated with xenografts of a human papillary thyroid carcinoma cell line, K1)	[^{211}At]At-astatide	100, 500 and 1000 kBq	high tumouricidal potential in NIS gene–transfected tumours without major side effects	[69]
Astatine-211	Healthy male Balb/C nu/nu mice	[^{211}At]At-AuNP (gold nanoparticles)	900 kBq	high in vitro and in vivo stability	[70]
Astatine-211	Male nude BALB/c- nu-nu (s.c. inoculated PANC-1 cells)	[^{211}At]At-FAPI-1;<br>[^{211}At]At- FAPI-5	540 – 970 kBq	higher tumour retention of [^{211}At]At- FAPI(s) compared with [^{131}I]I -FAPI(s)	[71]
Lead-212	Model A - Female naïve CD-1Elite mice;<br>Model B – Female Athymic mice bearing AR42J tumour	[^{212}Pb]Pb-PSC-PEG-T	Model A- Single injection of 74 kBq;<br>Model B- Single injection of 3.7 MBq	Model A - fast clearance from blood circulation, cleared through the kidneys.<br>Model B - prolonged accumulation in tumour and minimal retention in kidneys (0.9%ID in tumour; 1%ID in kidneys)	[72]

	Xenografts				
Lead-212	Female athymic-NCR- nude mice with SK-OV-3 tumour xenografts: Model A - tumour volume 15 mm3 Model B – tumour volume 146 mm3	[^{212}Pb]Pb-DOTA-AE1	Model A - Single injection of 740 kBq; Model B – Single injection of 925 kBq	Model A – the rate of tumour growth was inhibited in the period after the [^{212}Pb]Pb-DOTA-AE1 therapy; Model B - [^{212}Pb]Pb-DOTA-AE1 did not provide effective therapy for large established tumours.	[73]
Lead-212	Male non-obese, diabetic/Shi-scid/IL-2rgnull (NSG) mice: Model A - bearing PSMA(+) PC3 PIP tumour xenografts. Tumour volume 60–100 mm3. Model B - PSMA(+) micrometastatic model, mice were injected intravenously with 1 x 106 PC3-ML-Luc-PSMA cells	[^{212}Pb]Pb-L2	Model A - Single dose of 3.7 MBq Model B - 0, 0.7, 1.5, or 3.7 MBq	Model A - A single administration of 1.5 or 3.7 MBq showed significant tumour growth delay only in PSMA(+) Model B - the median survival time for the mice administered [^{212}Pb]Pb-L2 (3.7 MBq) was 58 days, demonstrating moderate but significant improvement.	[74]
Lead-212	Athymic Nude-Foxn1nu mice bearing C4-2 tumour xenografts. Tumour volume 250-1000 mm3	[^{212}Pb]Pb-NG001; [^{212}Pb]Pb-PSMA-617	Single dose of 10-56 kBq of [^{212}Pb]Pb-NG001; A single dose of 79 kBq of [^{212}Pb]Pb-PSMA-617	The uptake values (%ID/g) for tumour and kidneys at 2-hour post-injection were 17.61±6.76 and 21.07±10.33 for [^{212}Pb]Pb-NG001 and 17.93±2.90 and 52.82±26.62 for [^{212}Pb]Pb-PSMA-617	[75]
Lead-212	SCID mice bearing PC3 tumour xenografts	[^{212}Pb]Pb-RM2	Single dose of 1.85 MBq or 3.7 MBq	Both [^{212}Pb]Pb-RM2 treatment groups (1.85 MBq or 3.7MBq) demonstrated initial tumour control for 4-5 weeks post-treatment. 18 days pi, tumour regression was observed in the 3.7 MBq group (maximum per cent change of -49.3%) 40 days pi, tumour regrowth was observed in the 3.7 MBq group (+91.6% change from predose)	[76]
Terbium-149	SCID mouse model of leukaemia	[^{149}Tb]Tb-rituximab	5.5MBq labelled antibody conjugate (1.11GBq/mg) 2 days after an intravenous graft of 5106 Daudi cells	Tumour-free survival for >120 days in 89% of treated animals	[77]
Terbium-149	Tumour-bearing mice	[^{149}Tb]Tb-cm09 (DOTA-folate conjugate)	Group A: saline only Group B: 2.2 MBq; Group C: 3.0 MBq;	A significant tumour growth delay was found in treated animals resulting in an increased average survival time of mice which received 149Tb-cm09 (B: 30.5 d; C: 43 d) compared to untreated controls (A: 21 d).	[78]
Radium-223	Balb/c	[^{223}Ra]$RaCl_2$	450 kBq/kg of ^{223}Ra	High activity concentration in bone; High retention in the kidney and spleen among OARs	[79]
Radium-223	Balb/c	[^{223}Ra]$RaCl_2$	1250, 2500, 3750 kBq/kg	Minimal to moderate depletion of osteocytes and osteoblasts	[80]

Radium-223	Intratibial LNCaP or LuCaP 58	[^{223}Ra]RaCl_2	300 kBq/kg – 2 cycles	Inhibition of tumour cellular growth	[81]
Thorium-227	Human lymphoma Raji	[^{22t}Th]Th -Rituximab	50, 200, 1000 kBq/kg	Complete regression in 60% of mice treated with 200 kBq/kg	[82]
Thorium-227	HER2-overexpressing subcutaneous SKOV-3 or SKBR-3	[^{22t}Th]Th-trastuzumab	1000 kBq/kg - 1 cycle; 250 kBq/kg - 4 cycles	Survival with a tumour diameter of less than 16 mm was prolonged	[83]
Thorium-227	subcutaneous xenograft mouse model using HL- 60 cells at a single dose regimen	[^{22t}Th]Th-CD33-TTC	50, 150, or 300 kBq/kg – 1 cycle a second injection of 150 kBq/kg for some animals	Dose- dependent significant survival benefit	[84]
Thorium-227	NCI-H716, SNU- 16, and MFM-223	[^{22t}Th]Th-FGFR2-TTC	500 kBq/kg	significant inhibition of tumour growth at a dose of 500 kBq/kg	[85]

Table S4: Overview of some of the current/ongoing clinical studies registered in ClinicalTrials.gov.

NCT Number	Radio	Radiopharma-ceutical	Study Title	Study Status	Conditions	Sponsor	Phases
**NCT06939036**	Act-225	[^{225}Ac]Ac-SSO110	Study of [^{225}Ac]Ac-SSO110 in Subjects With ES-SCLC or MCC (SANTANA-225)	Ongoing, estimated completion 2026-12	Small Cell Lung Cancer Extensive Stage|Merkel Cell Carcinoma	Ariceum Therapeutics GmbH	Phase I/II
**NCT06888323**	Actinium-225	[^{225}Ac]Ac-lintuzumab	Testing an Anti-cancer Radio-Active Immunotherapy Called [^{225}Ac]Ac-lintuzumab in Patients With High-Risk Myelodysplastic Syndrome That Has Not Responded to Other Treatment	Not yet recruting	Refractory Myelodysplastic Syndrome	National Cancer Institute (NCI)	Phase I
**NCT06881823**	Actinium-225	[^{225}Ac]Ac-PSMA-R2 (AAA802); [^{177}Lu]Lu-PSMA-R2 (AAA602)	Study to Assess [^{177}Lu]Lu-PSMA-R2 (AAA602) and [225Ac]Ac-PSMA-R2 (AAA802) in Participants With PSMA-positive HRLPC	Not yet recruting	Prostate Cancer	Novartis Pharmaceuticals	Phase I/II
**NCT06879041**	Actinium-225	[^{225}Ac]Ac-AZD2284	A Phase I Study of [^{225}Ac]Ac-AZD2284 in Patients With Metastatic Castration-Resistant Prostate Cancer	Ongoing, estimated completion 2029-04	Metastatic Castration-Resistant Prostate Cancer	AstraZeneca	Phase I
**NCT06802523**	Actinium-225	[^{225}Ac]Ac-lintuzumab	Testing the Combination of Targeted Radiotherapy With Anti-Cancer Drugs, Venetoclax and ASTX-727, to Improve Outcomes for Adults With Newly Diagnosed Acute Myeloid Leukemia	Not yet recruting	Acute Myeloid Leukemia	National Cancer Institute (NCI)	Phase I
**NCT06736418**	Actinium-225	[^{225}Ac]Ac-ABD147	Study of [^{225}Ac]Ac-ABD147to Establish Optimal Dose in Patients With SCLC and LCNEC of the Lung That Previously Received Platinum-based Chemotherapy	Ongoing, estimated completion 2027-01	Small-Cell Lung Cancer (SCLC)|Large Cell Neuroendocrine Carcinoma of the Lung	Abdera Therapeutics Inc.	Phase I
**NCT06726161**	Actinium-225	[^{225}Ac]Ac-RYZ811; [^{225}Ac]Ac-RYZ801	Study of the Theranostic Pair RYZ811 (Diagnostic) and RYZ801 (Therapeutic) to Identify and Treat Subjects With GPC3+ Unresectable HCC	Ongoing, estimated completion 2031-01	HCC	RayzeBio, Inc.	Phase I
**NCT06590857**	Actinium-225	[^{225}Ac]Ac-DOTATATE (RYZ101)	Trial of [^{225}Ac]Ac-DOTATATE (RYZ101) in Subjects with ER+, HER2-negative Unresectable or Metastatic Breast Cancer Expressing SSTRs.	Ongoing, estimated completion 2033-01	Metastatic Breast Cancer HER2-negative ER+	RayzeBio, Inc.	Phase I/II
**NCT06287944**	Actinium-225	[^{225}Ac]Ac-DOTA-Daratumumab	[^{225}Ac]Ac-DOTA -Anti-CD38 Daratumumab Monoclonal Antibody With Fludarabine, Melphalan and Total Marrow and Lymphoid Irradiation as Conditioning Treatment for Donor Stem Cell Transplant in Patients With High-Risk Acute Myeloid Leukemia, Acute Lymphoblastic Leukemia and Myelodysplastic Syndrome	Ongoing, estimated completion 2028-05	Acute Lymphoblastic Leukemia; Acute Myeloid Leukemia; Myelodysplastic Syndrome	City of Hope Medical Center	Phase I

**NCT06229366**	Actinium-225	[225Ac]Ac-PSMA-62	[225Ac]Ac-PSMA-62 Trial in Oligometastatic Hormone Sensitive and Metastatic Castration Resistant Prostate Cancer	Ongoing, estimated completion 2027-09	Prostate Cancer	Eli Lilly and Company	Phase I
**NCT05983198**	Actinium-225	[225Ac]Ac-PSMA-R2	Phase I/II Study of [225Ac]Ac-PSMA-R2 in PSMA-positive Prostate Cancer, With/ Without Prior [177Lu]Lu-PSMA RLT (SatisfACtion)	Ongoing, estimated completion 2029-11	mCRPC treated with prior ARPI in post- 177Lu and pre-177Lu settings	Novartis Pharmaceuticals	Phase I/II
**NCT05605522**	Actinium-225	[225Ac]Ac-FPI-2059	A Study of [225Ac]Ac-FPI-2059 in Adult Participants With Solid Tumours	Active not recruting, estimated completion 2025-09	NTSR1-positive solid tumours refractory to standard therapies	Fusion Pharmaceuticals Inc.	Phase I
**NCT05595460**	Actinium-225	[225Ac]Ac-DOTATATE (RYZ101)	Study of RYZ101 in Combination With SoC in Subjects With SSTR+ ES-SCLC	Ongoing, estimated completion 2029-03	SSTR2-positive extensive-stage small- cell lung cancer	RayzeBio, Inc.	Phase I
**NCT05567770**	Actinium-225	[225Ac]Ac-J591	Actinium-J591 Radionuclide Therapy in PSMA-Detected Metastatic HOrmone-Sensitive Recurrent Prostate CaNcer	WITHDRAWN	Prostate Cancer Metastatic	Weill Medical College of Cornell University	Phase I
**NCT05477576**	Actinium-225	[225Ac]Ac-DOTATATE (RYZ101)	Study of RYZ101 Compared With SOC in Pts w Inoperable SSTR+ Well-differentiated GEP-NET That Has Progressed Following 177Lu-SSA Therapy	Ongoing, estimated completion 2028-07	SSTR2-positive gastroenteropancreatic neuroendocrine tumours with prior 177Lu therapy	RayzeBio, Inc.	Phase III
**NCT05363111**	Actinium-225	[225Ac]Ac-DOTA-daratumuab	Radioimmunotherapy [111I]I/[225Ac]Ac-DOTA -daratumumab) for the Treatment of Relapsed/Refractory Multiple Myeloma	Ongoing, estimated completion 2025-06	Relapsed or refractory multiple myeloma after at least 2 lines of prior therapy	City of Hope Medical Center	Phase I
**NCT05219500**	Actinium-225	[225Ac]Ac-FPI-2265 (PSMA-I&T)	Targeted Alpha Therapy With [225Ac]Ac-FPI-2265-Prostate Specific Membrane Antigen (PSMA)-I&T of Castration-resISTant Prostate Cancer (TATCIST)	Active, not recruting, estimated completion 2025-07	mCRPC with prior ARPI	Fusion Pharmaceuticals	Phase II
**NCT05204147**	Actinium-225	[225Ac]Ac-DOTA-M5A	Actinium 225 Labeled Anti-CEA Antibody ([225Ac]Ac-DOTA-M5A) for the Treatment of CEA Producing Advanced or Metastatic Cancers	Ongoing, estimated completion 2025-08	Metastatic solid tumours expressing CEA	City of Hope Medical Center	Phase I
**NCT04946370**	Actinium-225	[225Ac]Ac-J591	Phase I/II Trial of Pembrolizumab and Androgen-receptor Pathway Inhibitor With or Without [225Ac]Ac-J591for Progressive Metastatic Castration Resistant Prostate Cancer	Ongoing, estimated completion 2028-06	mCRPC treated with prior ARPI	Weill Medical College of Cornell University	Phase I/II
**NCT04886986**	Actinium-225	[225Ac]Ac-J591 with [177Lu]Lu-PSMA-I&T	Phase I/II [225Ac]Ac-J591 Plus [177Lu]Lu-PSMA-I&T for Progressive Metastatic Castration Resistant Prostate Cancer	Suspended, estimated completion 2027-12	mCRPC treated with prior ARPI	Weill Medical College of Cornell University	Phase I/II
**NCT04644770**	Actinium-225	[225Ac]Ac DOTA-h11B6 (JNJ-69086420)	A Study of JNJ-69086420, an Actinium-225-Labeled Antibody Targeting Human Kallikrein-2 (hK2) for Advanced Prostate Cancer	Ongoing, estimated completion 2025-12	mCRPC with prior ARPI	Janssen Research & Development, LLC	Phase I
**NCT04597411**	Actinium-225	[225Ac]Ac-PSMA-617	Study of [225Ac]Ac-PSMA-617 in Men With PSMA-positive Prostate Cancer	Ongoing, estimated completion 2027-01	mCRPC	Endocyte	Phase I

**NCT04576871**	Actinium-225	[^{225}Ac]Ac-J591	Re-treatment [^{225}Ac]Ac-J591for mCRPC	Active non recruting, estimated completion 2026-12	mCRPC treated with prior ARPI	Weill Medical College of Cornell University	Phase I
**NCT04506567**	Actinium-225	[^{225}Ac]Ac-J591	Fractionated and Multiple Dose [^{225}Ac]Ac-J591for Progressive mCRPC	Active non recruting, estimated completion 2027-06	mCRPC treated with prior ARPI	Weill Medical College of Cornell University	Phase I/II
**NCT03932318**	Actinium-225	[^{225}Ac]Ac-Lintuzumab	Venetoclax, Azacitidine, and [^{225}Ac]Ac-Lintuzumab in AML Patients	WITHDRAWN	Acute Myeloid LeukemiaRelapsed Adult AML	Actinium Pharmaceuticals	Phase I/II
**NCT03867682**	Actinium-225	[^{225}Ac]Ac-Lintuzumab	Venetoclax and [^{225}Ac]Ac-Lintuzumab in AML Patients	Unknown status	Relapsed/refractory AML	Actinium Pharmaceuticals	Phase I/II
**NCT03746431**	Actinium-225	[^{225}Ac]Ac-FPI-1434	A Phase 1/2 Study of [^{225}Ac]AcFPI-1434 Injection	Ongoing, estimated completion 2026-06	IGF-1R-positive solid tumours refractory to standard therapies	Fusion Pharmaceuticals	Phase I/II
**NCT03705858**	Actinium-225	[^{225}Ac]Ac-Lintuzumab	[^{225}Ac]Ac -Lintuzumab in Patients With Acute Myeloid Leukemia	WITHDRAWN	Acute Myeloid Leukemia	Joseph Jurcic, Columbia University	Phase I
**NCT03441048**	Actinium-225	[^{225}Ac]Ac-Lintuzumab	[^{225}Ac]Ac-Lintuzumab in Combination with Cladribine + Cytarabine + Filgastrim + Mitoxantrone (CLAG-M) for Relapsed/Refractory Acute Myeloid Leukemia	Completed; 2024-05	Acute Myeloid Leukemia	Medical College of Wisconsin	Phase I
**NCT03276572**	Actinium-225	[^{225}Ac]Ac-J591	Phase I Trial of [^{225}Ac]Ac-J591 in Patients With mCRPC	Completed with results, 2023-09	mCRPC treated with prior ARPI	Weill Medical College of Cornell University	Phase I
**NCT02998047**	Actinium-225	[^{225}Ac]Ac-Lintuzumab	A Phase I Study of [^{225}Ac]Ac-Lintuzumab in Patients With Refractory Multiple Myeloma	Terminated, 2020-05	Refractory Multiple Myeloma	Actinium Pharmaceuticals	Phase I
**NCT00672165**	Actinium-225	[^{225}Ac]Ac-Lintuzumab	Targeted Atomic Nano-Generators (Actinium-225-Labeled Humanised Anti-CD33 Monoclonal Antibody HuM195) in Patients With Advanced Myeloid Malignancies	Completed, 2015-02	Leukemia, Myelodisplastic syndrome	Memorial Sloan Kettering Cancer Center	Phase I
**NCT00014495**	Bismuth-213	[213Bi]Bi-Lintuzumab-(Bi213 MOAB M195)	Chemotherapy and Monoclonal Antibody Therapy in Treating Patients With Advanced Myeloid Cancer	Completed, 2009-12	LeukemiaMyelodysplastic SyndromesMyelodysplastic/Myeloproliferative Neoplasms	Memorial Sloan Kettering Cancer Center	Phase I/II
**NCT06441994**	Astatine-211	PSW-1025 ([^{211}At]At-PSMA-5)	Clinical Trial of Targeted Alpha Therapy Using [^{211}At]At-PSMA-5] for Prostate Cancer	Ongoing, estimated completion 2027-03	Prostate Cancer	Osaka University	Phase I
**NCT05275946**	Astatine-211	TAH-1005 ([^{211}At] NaAt)	Targeted Alpha Therapy Using Astatine-211 Against Differentiated Thyroid Cancer	Completed, 2025-03	Thyroid Cancer	Osaka University	Phase I
**NCT04579523**	Astatine-211	[^{211}At]At - OKT10-B10	[^{211}At]At -OKT10-B10and Fludarabine Alone or in Combination With Cyclophosphamide and Low-Dose TBI Before Donor Stem Cell Transplant for the	Not yet recruting, estimated completion 2028-12	Multiple Myeloma∣Recurrent Multiple Myeloma∣Refractory Multiple Myeloma	Fred Hutchinson Cancer Center	Phase I

			Treatment of Newly Diagnosed, Recurrent, or Refractory High-Risk Multiple Myeloma				
**NCT04466475**	Astatine-211	[^{211}At]At-OKT10-B10	Radioimmunotherapy [^{211}At]At -OKT10-B10 and Chemotherapy (Melphalan) Before Stem Cell Transplantation for the Treatment of Multiple Myeloma	WITHDRAWN	Plasma Cell Myeloma	Fred Hutchinson Cancer Center	Phase I
**NCT04461457**	Astatine-211	[^{211}At]At-MX35 F(ab')2	Targeted Radiation Therapy for Ovarian Cancer: Intraperitoneal Treatment With [^{211}At]At-MX35 F(ab')2	Completed, 2012-01	Ovarian Cancer	Vastra Gotaland Region	Early Phase I
**NCT04083183**	Astatine-211	[^{211}At]At-BC8-B10 Monoclonal Antibody	Total Body Irradiation and [^{211}At]At-BC8-B10 Monoclonal Antibody for the Treatment of Nonmalignant Diseases	Ongoing, estimated completion 2028-01	Non-Malignant Neoplasm	Fred Hutchinson Cancer Center	Phase I/II
**NCT03670966**	Astatine-211	[^{211}At]At-BC8-B10	[^{211}At]At-BC8-B10 Followed by Donor Stem Cell Transplant in Treating Patients With Relapsed or Refractory High-Risk Acute Leukemia or Myelodysplastic Syndrome	Ongoing, estimated completion 2029-03	hematology plan	Fred Hutchinson Cancer Center	Phase I/II
**NCT00003461**	Astatine-211	[^{211}At]At-monoclonal antibody 81C6	Radiolabeled Monoclonal Antibody Therapy in Treating Patients With Primary or Metastatic Brain Tumours	Completed, 2005-02	Brain and Central Nervous System TumoursMetastatic CancerNeuroblastoma	Duke University	Phase I/II
**NCT06710756**	Lead-212	[^{212}Pb]Pb-At PSV359	[^{212}Pb]Pb-At PSV359 Therapy for Patients With Solid Tumours	Ongoing, estimated completion 2032-05	Pancreatic Ductal AdenocarcinomaGastric CancerEsophageal CancerColorectal CancerOvarian CancerHead and Neck Cancer	Perspective Therapeutics	Phase I/II
**NCT06479811**	Lead-212	[^{203}Pb]Pb-VMT-alpha-NET; [^{212}Pb]Pb-VMT-alpha-NET	[^{212}Pb]Pb-VMT-Alpha-NET in Metastatic or Inoperable Somatostatin-Receptor Positive Gastrointestinal Neuroendocrine Tumours, Pheochromocytoma/Paragangliomas, Small Cell Lung, Renal Cell, and Head and Neck Cancers	Not yet recruting, estimated completion 2032-01	Head and Neck TumoursKidney CancersSmall Cell Lung CancersPheochromocytoma/ParagangliomasGastrointestinal Neuroendocrine TumoursSomatostatin Receptor Positive	National Cancer Institute (NCI)	Phase I
**NCT06427798**	Lead-212	[^{203}Pb]Pb-VMT-alpha-NET; [^{212}Pb]Pb]VMT-alpha-NET	Somatostatin-Receptors (SSTR)-Agonist [^{212}Pb]Pb-VMT-alpha-NET in Metastatic or Inoperable SSTR+ Gastrointestinal Neuroendocrine Tumour and Pheochromocytoma/Paraganglioma Previously Treated With Systemic Targeted Radioligand Therapy	Ongoing, estimated completion 2039-07	Somatostatin Receptor PositiveGastrointestinal Neuroendocrine TumoursPheochromocytomaParagangliomas	National Cancer Institute (NCI)	Phase I/II
**NCT06148636**	Lead-212	[^{212}Pb]Pb-VMT-alpha-NET; [^{212}Pb]Pb-VMT-alpha-NET	A Safety Study of [^{212}Pb]Pb-VMT-alpha-NET in Patients With Neuroendocrine Tumours	Active not recruting, estimated completion 2027-11	Neuroendocrine Tumours	David Bushnell	Early Phase I
**NCT05725070**	Lead-212	[^{212}Pb]Pb -NG001	Phase 0/1 Study of [^{212}Pb]Pb -NG001 in mCRPC	Completed, 2023-07	Metastatic Castration-resistant Prostate Cancer	ARTBIO Inc.	Early Phase I
**NCT05720130**	Lead-212	[^{212}Pb]Pb-ADVC001	Phase Ib/IIa Dose Escalation and Expansion Study of [^{212}Pb]Pb-ADVC001 in Metastatic Castration Resistant Prostate Cancer (TheraPb - Phase I/II Study).	Ongoing, estimated completion 2029-12	mCRPC with prior ARPI and no prior exposure to 177Lu	AdvanCell Pty Limited	Phase I/II

**NCT05655312**	Lead-212	[^{203}Pb]Pb-VMT01; [^{212}Pb]Pb-VMT01	MC1R-targeted Alpha-particle Monotherapy and Combination Therapy Trial With Nivolumab in Adults With Advanced Melanoma	Ongoing, estimated completion 2029-12	Melanoma	Perspective Therapeutics	Phase I/II
**NCT05636618**	Lead-212	[212Pb]VMT-α-NET; [212Pb]VMT-α-NET	Targeted Alpha-Particle Therapy for Advanced SSTR2 Positive Neuroendocrine Tumours	Ongoing, estimated completion 2029-12	Metastatic Castration-resistant Prostate Cancer	Perspective Therapeutics	Phase I/II
**NCT05557708**	Lead-212	[^{203}Pb]Pb-Pentixather; [^{212}Pb]Pb-Pentixather	A Safety Study of [^{212}Pb]Pb-Pentixather Radioligand Therapy	Not yet recruting, estimated completion 2030-06	Carcinoid Tumour LungNeuroendocrine Tumour of the LungCarcinoma, Small-Cell Lung	Yusuf Menda	Early Phase I
**NCT05283330**	Lead-212	[^{212}Pb]Pb-DOTAM-GRPR1	Safety and Tolerability of [^{212}Pb]Pb-DOTAM-GRPR1 in Adult Subjects With Recurrent or Metastatic GRPR-expressing Tumours	Ongoing, estimated completion 2027-08	GRPR1-positive solid tumours refractory to standard therapies	Orano Med LLC	Phase I
**NCT05153772**	Lead-212	[^{212}Pb]Pb-DOTAMTATE	Targeted Alpha-emitter Therapy of PRRT Naïve and Previous PRRT Neuroendocrine Tumour Patients	Active not recruting, estimated completion 2028-10	Neuroendocrine Tumours	Orano Med LLC	Phase II
**NCT03466216**	Lead-212	[^{212}Pb]Pb-DOTAMTATE	Phase 1 Study of AlphaMedix™ in Adult Subjects With SSTR (+) NET	Terminated, 2023-04	SSTR2-positive neuroendocrine tumours refractory to standard therapies	Radiomedix and Orano Med	Phase I
**NCT01384253**	Lead-212	[^{212}Pb]Pb-TCMC-Trastuzumab	Safety Study of [^{212}PbPb -TCMC-Trastuzumab Radio Immunotherapy	Completed, 2016-07	Breast NeoplasmsPeritoneal NeoplasmsOvarian NeoplasmsPancreatic NeoplasmsStomach Neoplasms	Orano Med LLC	Phase I
**NCT05924672**	Radium-223	[^{223}Ra]RaCl$_2$	Efficacy of Radium-223 in PSMA PET Optimally Selected Patients	Ongoing, estimated completion 2028-05	Castration-Resistant Prostate Carcinoma丨Metastatic Malignant Neoplasm in the Bone丨Stage IVB Prostate Cancer AJCC v8	University of California, San Francisco	Phase II
**NCT05301062**	Radium-223	[^{223}Ra]RaCl$_2$ (BAY88-8223)	A Research Called CREDIT Studies How Safe the Study Treatment Radium-223 is and How Well it Works in Chinese Men With Advanced Prostate Cancer That Has Spread to the Bones and Does Not Respond to Treatments for Lowering Testosterone Levels	Terminated, 2023-06	Metastatic Castration-resistant Prostate Cancer; Bone Metastases	Bayer	observational
**NCT05133440**	Radium-223	[^{223}Ra]RaCl$_2$	A Study of Stereotactic Body Radiation Therapy and [^{223}Ra]RaCl$_2$ in Prostate Cancer That Has Spread to the Bones	Active not recruting, estimated completion 2027-11	Prostate Cancer	Memorial Sloan Kettering Cancer Center	Phase II
**NCT04681144**	Radium-223	[^{223}Ra]RaCl$_2$ (BAY88-8223)	A Study to Learn More About How Radium-223 Affects the Quality of Life of Colombian Patients With Prostate Cancer That Has Not Responded to Testosterone Lowering Treatment and Has Spread to the Bones, and to Better Understand Its Safety	Completed, 2022-11	Prostate Cancer	Bayer	observational

**NCT04597125**	Radium-223	[^{223}Ra]$RaCl_2$ (BAY88-8223)	Investigation of [^{223}Ra]$RaCl_2$ (Xofigo), a Treatment That Gives Off Radiation That Helps Kill Cancer Cells, Compared to a Treatment That Inactivates Hormones (New Antihormonal Therapy, NAH) in Patients With Prostate Cancer That Has Spread to the Bone Getting Worse on or After Earlier NAH	Active not recruting, estimated completion 2026-10	Metastatic Castrate Resistant Prostate Cancer (mCRPC)	Bayer	Phase IV
**NCT04587427**	Radium-223	[^{223}Ra]$RaCl_2$	A Study to Learn More About How Radium-223 is Being Used With Other Treatments in European Patients Who Have Not Received Radium-223 Before	Completed, 2023-05	Bone Metastatic Castration-resistant Prostate Cancer	Bayer	observational
**NCT04521361**	Radium-223	[^{223}Ra]$RaCl_2$ (BAY88-8223)	A Study to Assess How Radium-223 Distributes in the Body of Patients With Prostate Cancer Which Spread to the Bones	Active not recruting, estimated completion 2025-09	Bone Metastatic Castration-resistant Prostate Cancer	Bayer	Phase I
**NCT04516161**	Radium-223	[^{223}Ra]$RaCl_2$ (BAY88-8223)	EPIX, a Study to Gather More Information About Characteristics of Patients and Other Factors Which May Contribute to Survival Over a Long Period of Time in Patients With Metastatic Castration-resistant Prostate Cancer (mCRPC) Treated With Radium-223 (Xofigo)	Completed, 2021-03	Metastatic Castration Resistant Prostate Cancer (mCRPC)	Bayer	observational
**NCT04489719**	Radium-223	[^{223}Ra]$RaCl_2$	Impact of DNA Repair Pathway Alterations on Sensitivity to Radium-223 in Bone Metastatic Castration-resistant Prostate Cancer	Ongoing, estimated completion 2029-08	Castration-Resistant Prostate Carcinoma; Metastatic Malignant Neoplasm in the Bone	University of Washington	observational
**NCT04281147**	Radium-223	[^{223}Ra]$RaCl_2$ (BAY88-8223)	Study to Gather Information About the Use of Healthcare Services and the Way the Disease is Cared for in Canadian Patients With Prostate Gland Cancer Which Spread Throughout the Body	Completed, 2021-06	Prostate Cancer	Bayer	observational
**NCT04256993**	Radium-223	[^{223}Ra]$RaCl_2$ (BAY88-8223)	PRECISE, a Study to Gather More Information About Bone Fractures and Survival in Castration-resistant Prostate Cancer (CRPC) patients Treated With Radium-223 in Routine Clinical practIce in SwedEn	Completed, 2021-06	Metastatic Castration-Resistant Prostate Cancer	Bayer	observational
**NCT04237584**	Radium-223	[^{223}Ra]$RaCl_2$	A Study Comparing ARB With Radium-223 vs ARB Therapy With Placebo and the Effect Upon Survival for mCRPC Patients	Terminated, 2022-03	Metastatic Castration-resistant Prostate Cancer	MANA RBM	Phase III
**NCT04232761**	Radium-223	[^{223}Ra]$RaCl_2$ (BAY88-8223)	Study to Gather Information on the Safety and How [^{223}Ra]$RaCl_2$, an Alpha Particle-emitting Radioactive Agent, Works Under Routine Clinical Practice in Taiwan in Patients With Castration-resistant Prostate Cancer (CRPC) Which Has Spread to the Bone	Completed, 2024-04	Castration-resistant Prostate Cancer	Bayer	observational

**NCT04110782**	Radium-223	$[^{223}Ra]RaCl_2$	Radical Prostatectomy and External Beam Radiotherapy in mCRPC With $[^{223}Ra]RaCl_2$ (RaProRad)	UNKNOWN	Prostate Cancer	Azienda Policlinico Umberto I	observational
**NCT04090398**	Radium-223	$[^{223}Ra]RaCl_2$	Testing the Addition of Radium Therapy ($[^{223}Ra]RaCl_2$) to the Usual Chemotherapy Treatment (Paclitaxel) for Advanced Breast Cancer That Has Spread to the Bones	Active not recruting, estimated completion 2026-06	Anatomic Stage IV Breast Cancer; Metastatic HER2-Negative Breast Carcinoma; Metastatic Malignant Neoplasm in the Bone	National Cancer Institute (NCI)	Phase II
**NCT04071236**	Radium-223	$[^{223}Ra]RaCl_2$	Radiation Medication ($[^{223}Ra]RaCl_2$) Versus $[^{223}Ra]RaCl_2$ Plus Radiation Enhancing Medication (M3814) Versus $[^{223}Ra]RaCl_2$ M3814 Plus Avelumab (a Type of Immunotherapy) for Advanced Prostate Cancer Not Responsive to Hormonal Therapy	Ongoing, estimated completion 2026-04	Metastatic Castration-Resistant Prostate Carcinoma; Metastatic Malignant Neoplasm in the Bone; Metastatic Malignant Neoplasm in the Lymph Nodes; Stage IVB Prostate Cancer	National Cancer Institute (NCI)	Phase I/II
**NCT04071223**	Radium-223	$[^{223}Ra]RaCl_2$	Testing the Addition of a New Anti-cancer Drug, $[^{223}Ra]RaCl_2$, to the Usual Treatment (Cabozantinib) for Advanced Renal Cell Cancer That Has Spread to the Bone, RadiCaL Study	Ongoing, estimated completion 2025-10	Advanced Renal Cell Carcinoma; Chromophobe Renal Cell Carcinoma; Clear Cell Renal Cell Carcinoma; Collecting Duct Carcinoma; Kidney Medullary Carcinoma; Metastatic Malignant Neoplasm in the Bone; Papillary Renal Cell Carcinoma|Stage IV Renal Cell Cancer; Unclassified Renal Cell Carcinoma	National Cancer Institute (NCI)	Phase II
**NCT03996473**	Radium-223	$[^{223}Ra]RaCl_2$ (BAY88-8223)	Study to Test the Safety and How $[^{223}Ra]RaCl_2$ an Alpha Particle-emitting Radioactive Agent Works in Combination With Pembrolizumab an Immune Checkpoint Inhibitor in Patients With Stage IV Non-small Cell Lung Cancer With Bone Metastases	Terminated, 2023-01	Carcinoma, Non-Small-Cell Lung	Bayer	Phase I
**NCT03903835**	Radium-223	$[^{223}Ra]RaCl_2$	ProBio: A Biomarker Driven Study in Patients With Metastatic Prostate Cancer	Ongoing, estimated completion 2026-12	Metastatic Castration-resistant Prostate Cancer (mCRPC); Metastatic Hormone-Sensitive Prostate Cancer (mHSPC)	Karolinska Institutet	Phase III
**NCT03896984**	Radium-223	$[^{223}Ra]RaCl_2$ (BAY88-8223)	Descriptive Analysis of Clinical Outcomes in Patients With Prostate Gland Cancer, Which Spreads to Other Parts of the Body, Who Were Treated First With Novel Anti-hormone Therapy Followed by a Second Line Treatment With Novel Anti-Hormone Therapy or RadIum-223 (Xofigo).	Completed, 2020-12	Metastatic Castration-resistant Prostate Cancer (mCRPC)	Bayer	observational
**NCT03737370**	Radium-223	$[^{223}Ra]RaCl_2$	Fractionated Docetaxel and Radium-223 in Metastatic Castration-Resistant Prostate Cancer	Active not recruting, estimated completion 2026-12	Metastatic Castrate Resistant Prostate Cancer	Tufts Medical Center	Phase I
**NCT03563014**	Radium-223	$[^{223}Ra]RaCl_2$ (Xofigo, Bay88-8223)	A Local Retrospective Observational Study to Evaluate the Treatment Patterns of mCRPC Patients in Belgium Treated With Radium-223	Completed, 2019-01	Prostatic Neoplasms, Castration-Resistant	Bayer	observational

**NCT03458559**	Radium-223	$[^{223}Ra]RaCl_2$	Rhenium-188-HEDP vs. $[^{223}Ra]RaCl_2$ in Patients With Advanced Prostate Cancer Refractory to Hormonal Therapy	UNKNOWN	Prostate Cancer Metastatic to Bone	Amsterdam UMC, location VUmc	Phase III
**NCT03419442**	Radium-223	$[^{223}Ra]RaCl_2$	Multi-academic Center Study of Xofigo Patients	Completed, 2019-10	Prostate Cancer, Castration Resistant	Bayer	observational
**NCT03368989**	Radium-223	$[^{223}Ra]RaCl_2$	The Effects of $[^{223}Ra]RaCl_2$ Therapy on Radionuclide Bone Scan Lesions.	Completed, 2017-02	Bony Metastases From Castrate Refractory Prostate Cancer	The University of Texas Health Science Center, Houston	observational
**NCT03361735**	Radium-223	$[^{223}Ra]RaCl_2$	Radium $[^{223}Ra]RaCl_2$, Hormone Therapy and Stereotactic Body Radiation Therapy in Treating Patients With Metastatic Prostate Cancer	Active not recruting, estimated completion 2026-02	Prostate Adenocarcinoma	City of Hope Medical Center	Phase II
**NCT03344211**	Radium-223	$[^{223}Ra]RaCl_2$	Enzalutamide With or Without $[^{223}Ra]RaCl_2$ in Patients With Metastatic, Castration-Resistant Prostate Cancer	Active not recruting, estimated completion 2025-11	Bone Metastatic Castration-resistant Prostate Cancer	University of Southern California	Phase II
**NCT03325127**	Radium-223	$[^{223}Ra]RaCl_2$	Outcomes of mCRPC Patients Treated With Radium-223 Concomitant With Abiraterone or Enzalutamide- A Chart Review Study	WITHDRAWN	Prostatic Neoplasms, Castration-Resistant	Bayer	observational
**NCT03317392**	Radium-223	$[^{223}Ra]RaCl_2$	Testing the Safety of Different Doses of Olaparib Given Radium-223 for Men With Advanced Prostate Cancer With Bone Metastasis	Active not recruting, estimated completion 2026-04	Castration-Resistant Prostate Carcinoma; Metastatic Prostate Adenocarcinoma	National Cancer Institute (NCI)	Phase I/II
**NCT03315260**	Radium-223	$[^{223}Ra]RaCl_2$ (BAY88-8223)	Treatment Satisfaction With Radium-223 in Japan	Completed, 2023-03	Prostatic Neoplasms	Bayer	observational
**NCT03304418**	Radium-223	$[^{223}Ra]RaCl_2$	Radium-223 and Radiotherapy in Hormone-Naïve Men With Oligometastatic Prostate Cancer to Bone	Completed, 2023-08	Prostate Cancer Metastatic to Bone	University of Utah	Phase II
**NCT03223597**	Radium-223	$[^{223}Ra]RaCl_2$	Registry of Treatment Outcomes of Symptomatic Metastasized Castration Resistant Prostate Cancer Treated With Radium-223	Completed, 2018-03	Prostate Cancer Metastatic; Bone Metastases	The Netherlands Cancer Institute	observational
**NCT03093428**	Radium-223	$[^{223}Ra]RaCl_2$	Study Evaluating the Addition of Pembrolizumab to Radium-223 in mCRPC	Completed, 2025-02	Prostate Cancer	Dana-Farber Cancer Institute	Phase II
**NCT03076203**	Radium-223	$[^{223}Ra]RaCl_2$	Phase IB Trial of Radium-223 and Niraparib in Patients With Castrate Resistant Prostate Cancer (NiraRad)	Completed, 2022-11	Bone-only Metastatic Castration-Resistant Prostate Cancer (CRPC)	Sidney Kimmel Cancer Center at Thomas Jefferson University	Phase I
**NCT03062254**	Radium-223	$[^{223}Ra]RaCl_2$	Metabolic Change in Prostate Cancer Bone Metastases on $[^{68}Ga]Ga$-HBED-CC-PSMA PET/CT Following Radium-223 Therapy	Completed, 2021-07	Prostate Cancer	Sir Mortimer B. Davis - Jewish General Hospital	Phase II
**NCT02928029**	Radium-223	$[^{223}Ra]RaCl_2$ (BAY88-8223)	Study Testing $[^{223}Ra]RaCl_2$ in Relapsed Multiple Myeloma	Terminated, 2019-03	Multiple Myeloma	Bayer	Phase I/II

**NCT02925702**	Radium-223	$[^{223}Ra]RaCl_2$ 55mBq/Kg every 4 weeks intravenously	PRORADIUM: Prospective Multi-centre Study of Prognostic Factors in mCRPC Patients Treated With Radium-223.	UNKNOWN	Advanced Prostate Cancer|Castration Resistant	Centro Nacional de Investigaciones Oncologicas CARLOS III	observational
**NCT02903160**	Radium-223	$[^{223}Ra]RaCl_2$	Prostate Cancer Intensive, Non-Cross Reactive Therapy (PRINT) for Castration Resistant Prostate Cancer (CRPC)	Completed, 2021-11	Prostate Cancer	Icahn School of Medicine at Mount Sinai	Phase II
**NCT02899104**	Radium-223	$[^{223}Ra]RaCl_2$ (BAY88-8223)	Navigant Study- Treatment Patterns in mCRPC (Metastatic Castrate Resistant Prostate Cancer)	Completed, 2019-03	Prostatic Neoplasms, Castration-Resistant	Bayer	observational
**NCT02880943**	Radium-223	$[^{223}Ra]RaCl_2$	Dose-finding, Safety and Efficacy Study of $[^{223}Ra]RaCl_2$ (XOFIGO) in RCC Patients With Bone Metastases. (EIFFEL)	UNKNOWN	Clear-cell Metastatic Renal Cell Carcinoma; Bone Metastases	Association Pour La Recherche des Thérapeutiques Innovantes en Cancérologie	Phase I/II
**NCT02814669**	Radium-223	$[^{223}Ra]RaCl_2$	Safety and Tolerability of Atezolizumab (ATZ) in Combination With $[^{223}Ra]RaCl_2$ (R-223-D) in Metastatic Castrate-Resistant Prostate Cancer (CRPC) Progressed Following Treatment With an Androgen Pathway Inhibitor	Completed, 2019-07	Castrate-Resistant Prostate Cancer	Hoffmann-La Roche	Phase I
**NCT02803437**	Radium-223	$[^{223}Ra]RaCl_2$ (BAY88-8223)	Drug Use Investigation of Xofigo, Castration Resistant Prostate Cancer With Bone Metastases	Completed, 2024-12	Prostatic Neoplasms, Castration-Resistant	Bayer	observational
**NCT02729103**	Radium-223	$[^{223}Ra]RaCl_2$	Treatment Patterns in Metastatic Prostate Cancer	Completed, 2017-01	Prostatic Neoplasm	Bayer	observational
**NCT02656563**	Radium-223	$[^{223}Ra]RaCl_2$	Radium-223 Following Intermittent ADT	WITHDRAWN	Prostate Cancer	Canadian Urology Research Consortium	Phase II
**NCT02605356**	Radium-223	$[^{223}Ra]RaCl_2$ (BAY88-8223)	Phase 1b/2 Study Testing $[^{223}Ra]RaCl_2$/Bortezomib/Dexamethasone Combination in Relapsed Multiple Myeloma	WITHDRAWN	Multiple Myeloma	Bayer	Phase I/II
**NCT02582749**	Radium-223	$[^{223}Ra]RaCl_2$	Androgen Deprivation Therapy +/- $[^{223}Ra]RaCl_2$ in Metastatic Prostate Cancer With Bone Metastases	Terminated, 2017-09	Prostate Cancer|Bone Metastases|Prostate Neoplasms	Ajjai Alva, MD	Phase II
**NCT02518698**	Radium-223	$[^{223}Ra]RaCl_2$ (BAY88-8223)	Treatment Patterns in Castrate Resistant Prostate Cancer Patients With Bone Metastases in a Medicare Population	Completed, 2017-09	Prostate Cancer	Bayer	observational
**NCT02507570**	Radium-223	$[^{223}Ra]RaCl_2$	Open Label Phase Two Study of Enzalutamide With Concurrent Administration of $[^{223}Ra]RaCl_2$ in Castration-Resistant (Hormone-Refractory) Prostate Cancer	Completed, 2019-01	Prostate Carcinoma Metastatic to the Bone	Carolina Research Professionals, LLC	Phase II

			Subjects With Symptomatic Bone Metastasis				
**NCT02484339**	Radium-223	[^{223}Ra]RaCl$_2$	Treatment of Advanced Castration Resistant Prostate Carcinoma With Limited Bone Metastases (α-RT)	UNKNOWN	Prostate Carcinoma	University Hospital Freiburg	Phase II
**NCT02463799**	Radium-223	[^{223}Ra]RaCl$_2$	Study of Sipuleucel-T W/ or W/O Radium-223 in Men With Asymptomatic or Minimally Symptomatic Bone-MCRPC	Completed, 2019-12	Prostate Cancer	Sidney Kimmel Comprehensive Cancer Center at Johns Hopkins	Phase II
**NCT02450812**	Radium-223	[^{223}Ra]RaCl$_2$ (BAY88-8223)	Non-interventional Study With Radium-223 Dichloride Assessing Overall Survival and Effectiveness Predictors for mCRPC Patients in a Real Life Setting in Germany	Completed, 2020-09	Prostatic Neoplasms, Castration-Resistant	Bayer	observational
**NCT02442063**	Radium-223	[^{223}Ra]RaCl$_2$	Phase Ib Study of Radium Ra 223 Dichloride in Combination With Paclitaxel in Cancer Subjects With Bone Lesions	Completed, 2016-10	Neoplasms;Bone Diseases	Bayer	Phase I
**NCT02406521**	Radium-223	[^{223}Ra]RaCl$_2$	Exploratory Study of Radium-223 and VEGF-Targeted Therapy in Patients With Metastatic Renal Cell Carcinoma and Bone Mets	Completed, 2019-12	Metastatic Renal Cell Carcinoma	Dana-Farber Cancer Institute	Phase I
**NCT02398526**	Radium-223	[^{223}Ra]RaCl$_2$ (BAY88-8223)	Pain Evaluation in Radium-223 Treated Castration Resistant Prostate Cancer Patients With Bone Metastases	Completed, 2020-07	Castration-Resistant Prostatic Cancer	Bayer	observational
**NCT02396368**	Radium-223	[^{223}Ra]RaCl$_2$	A Study of Radium-223 in Combination With Tasquinimod in Bone-only Metastatic Castration-Resistant Prostate Cancer	WITHDRAWN	Bone-only Metastatic Castration-Resistant Prostate Cancer (CRPC)	Sidney Kimmel Comprehensive Cancer Center at Johns Hopkins	Phase I
**NCT02390934**	Radium-223	[^{223}Ra]RaCl$_2$	Efficacy of Radium 223 in Radioactive Iodine Refractory Bone Metastases From Differentiated Thyroid Cancer	Completed, 2019-04	Thyroid Cancer	Gustave Roussy, Cancer Campus, Grand Paris	Phase II
**NCT02366130**	Radium-223	[^{223}Ra]RaCl$_2$	Trial of [^{223}Ra]RaCl$_2$ in Combination With Hormonal Therapy and Denosumab in the Treatment of Patients With Hormone-Positive Bone-Dominant Metastatic Breast Cancer	Completed, 2020-12	Breast Cancer	M.D. Anderson Cancer Center	Phase II
**NCT02346526**	Radium-223	[^{223}Ra]RaCl$_2$	A Biomarker Study of Standard-of-care [^{223}Ra]RaCl$_2$ for Metastatic Castration-resistant Prostate Cancer	Completed, 2020-12	Prostate Cancer; Castration-resistant Prostate Cancer; Castration-resistant Prostate Cancer Metastatic to Bone	Massachusetts General Hospital	Phase II
**NCT02331303**	Radium-223	[^{223}Ra]RaCl$_2$ (BAY88-8223)	A Drug Utilization Study of Radium-223 in Sweden	Completed, 2017-12	Neoplasms	Bayer	observational
**NCT02283749**	Radium-223	[^{223}Ra]RaCl$_2$	BrUOG L301 With Non-Small Cell Lung Cancer and Bone Metastases	Completed, 2018-11	Non Small Cell Lung Cancer With Bone Metastatses	Angela Taber MD	Phase II

**NCT02278055**	Radium-223	[^{223}Ra]$RaCl_2$	Non-Randomized Trial Assessing Pain Efficacy With Radium-223 in Symptomatic Metastatic Castration-Resistant Prostate Cancer	Completed, 2022-02	Metastatic Prostate Cancer|Pain	Memorial Sloan Kettering Cancer Center	Phase II
**NCT02258464**	Radium-223	[^{223}Ra]$RaCl_2$	Study of [^{223}Ra]$RaCl_2$ Versus Placebo and Hormonal Treatment as Background Therapy in Subjects With Bone Predominant HER2 (Human Epidermal Growth Factor Receptor 2) Negative Hormone Receptor Positive Metastatic Breast Cancer	Terminated, 2019-08	Breast Neoplasms	Bayer	Phase II
**NCT02258451**	Radium-223	[^{223}Ra]$RaCl_2$ (BAY88-8223)	Study of [^{223}Ra]$RaCl_2$ in Combination With Exemestane and Everolimus Versus Placebo in Combination With Exemestane and Everolimus in Subjects With Bone Predominant HER2 Negative Hormone Receptor Positive Metastatic Breast Cancer	Completed, 2022-10	Breast Neoplasms	Bayer	Phase II
**NCT02199197**	Radium-223	[^{223}Ra]$RaCl_2$	Radium-223 With Enzalutamide Compared to Enzalutamide Alone in Men With Metastatic Castration Refractory Prostate Cancer	Completed, 2019-10	Prostate Cancer	University of Utah	Phase II
**NCT02194842**	Radium-223	[^{223}Ra]$RaCl_2$	Phase III Radium-223 mCRPC-PEACE III	Active not recruting, estimated completion 2028-12	Prostate Cancer	European Organisation for Research and Treatment of Cancer - EORTC	Phase III
**NCT02141438**	Radium-223	[^{223}Ra]$RaCl_2$ (BAY88-8223)	Observational Study for the Evaluation of Long-term Safety of Radium-223 Used for the Treatment of Metastatic Castration Resistant Prostate Cancer	Completed, 2024-10	Metastatic Castration-resistant Prostate Cancer	Bayer	observational
**NCT02135484**	Radium-223	[^{223}Ra]$RaCl_2$ Alpharadin	Radium-223 in Castrate Resistant Prostate Cancer Bone Metastases	Completed, 2020-12	Prostate Cancer	M.D. Anderson Cancer Center	NA
**NCT02097303**	Radium-223	[^{223}Ra]$RaCl_2$	Open Label Phase Two Trial of [^{223}Ra]$RaCl_2$ With Concurrent Administration of Abiraterone Acetate Plus Prednisone in Symptomatic Castration-Resistant (Hormone-Refractory) Prostate Cancer Subjects With Bone Metastasis	Completed, 2015-12	Prostate Cancer	Carolina Research Professionals, LLC	Phase II
**NCT02043678**	Radium-223	[^{223}Ra]$RaCl_2$ (BAY88-8223)	[^{223}Ra]$RaCl_2$ and Abiraterone Acetate Compared to Placebo and Abiraterone Acetate for Men With Cancer of the Prostate When Medical or Surgical Castration Does Not Work and When the Cancer Has Spread to the Bone, Has Not Been Treated With Chemotherapy and is Causing no or Only Mild Symptoms	Completed, 2024-02	Prostatic Neoplasms	Bayer	Phase III

**NCT02034552**	Radium-223	[^{223}Ra]$RaCl_2$ (BAY88-8223)	A Randomized Phase IIa Efficacy and Safety Study of [^{223}Ra]$RaCl_2$ With Abiraterone Acetate or Enzalutamide in Metastatic Castration-resistant Prostate Cancer (CRPC)	Completed, 2018-06	Prostatic Neoplasms	Bayer	Phase II
**NCT02023697**	Radium-223	[^{223}Ra]$RaCl_2$ (BAY88-8223)	Standard Dose Versus High Dose and Versus Extended Standard Dose [^{223}Ra]$RaCl_2$ in Castration-resistant Prostate Cancer Metastatic to the Bone	Completed, 2018-08	Prostatic Neoplasms	Bayer	Phase II
**NCT01934790**	Radium-223	[^{223}Ra]$RaCl_2$ (BAY88-8223)	Re-treatment Safety of [^{223}Ra]$RaCl_2$ in Castration-resistant Prostate Cancer With Bone Metastases	Completed, 2017-04	Prostatic Neoplasms	Bayer	Phase I/II
**NCT01929655**	Radium-223	[^{223}Ra]$RaCl_2$ (BAY88-8223)	Japanese BAY88-8223 Monotherapy Phase II Study	Completed, 2017-05	Prostatic Neoplasms	Bayer	Phase II
**NCT01810770**	Radium-223	[^{223}Ra]$RaCl_2$ (BAY88-8223)	[^{223}Ra]$RaCl_2$ Asian Population Study in the Treatment of CRPC Patients With Bone Metastasis	Completed, 2017-09	Prostatic Neoplasms	Bayer	Phase III
**NCT01798108**	Radium-223	[^{223}Ra]$RaCl_2$ (BAY88-8223)	Dose Escalation Study of [^{223}Ra]$RaCl_2$ in Patients With Advanced Skeletal Metastases	Completed, 2003-06	Neoplasm Metastasis	Bayer	Phase I
**NCT01618370**	Radium-223	[^{223}Ra]$RaCl_2$ (BAY88-8223)	[^{223}Ra]$RaCl_2$ (Alpharadin) in Castration-Resistant (Hormone-Refractory) Prostate Cancer Patients With Bone Metastases	Completed, 2016-02	Prostatic Neoplasms	Bayer	Phase III
**NCT01565746**	Radium-223	[^{223}Ra]$RaCl_2$ (BAY88-8223)	Safety, Biodistribution, Radiation Dosimetry and Pharmacokinetics Study of BAY88-8223 in Japanese Patients	Completed, 2016-04	Prostatic Neoplasms	Bayer	Phase I
**NCT01106352**	Radium-223	[^{223}Ra]$RaCl_2$ (Xofigo, BAY88-8223)丨DRUG: Docetaxel	A Study of Alpharadin With Docetaxel in Patients With Bone Metastasis From Castration-Resistant Prostate Cancer (CRPC)	Completed, 2015-06	Bone Metastases丨Castration-Resistant Prostate Cancer	Bayer	Phase I/II
**NCT01070485**	Radium-223	[^{223}Ra]$RaCl_2$ (BAY88-8223)	BAY88-8223, Alpharadin, Breast Cancer Patients With Bone Dominant Disease	Completed, 2012-01	Breast Cancer丨Bone Metastases	Bayer	Phase II
**NCT00748046**	Radium-223	[^{223}Ra]$RaCl_2$ (BAY88-8223)	Alpharadin™ ([^{223}Ra]$RaCl_2$) Safety and Dosimetry With HRPC That Has Metastasized to the Skeleton	Completed, 2009-10	Prostate Cancer丨Metastases丨Pharmacokinetics	Bayer	Phase I
**NCT00699751**	Radium-223	[^{223}Ra]$RaCl_2$ (BAY88-8223)	A Phase III Study of [^{223}Ra]$RaCl_2$ in Patients With Symptomatic Hormone Refractory Prostate Cancer With Skeletal Metastases	Completed, 2014-02	Hormone Refractory Prostate Cancer丨Bone Metastases	Bayer	Phase III
**NCT00667537**	Radium-223	[^{223}Ra]$RaCl_2$ (BAY88-8223)	PK in Pts With HRPC & Skeletal Metastes	Completed, 2008-12	Prostatic Neoplasms	Bayer	Phase I
**NCT00667199**	Radium-223	[^{223}Ra]$RaCl_2$ (BAY88-8223)	BAY88-8223, Does Response Study in HRPC Patients	Completed, 2009-10	Hormone Refractory Prostate Cancer; Bone Metastases	Bayer	Phase II
**NCT00459654**	Radium-223	[^{223}Ra]$RaCl_2$ (BAY88-8223)	A Placebo-controlled Phase II Study of Bone-targeted Radium-223 in	Completed, 2007-05	Prostate Cancer丨Neoplasm Metastasis	Bayer	Phase II

			Symptomatic Hormone-refractory Prostate Cancer				
**NCT00337155**	Radium-223	$[^{223}Ra]RaCl_2$ (BAY88-8223)	BAY88-8223, Dose Finding Study in Patients With HRPC	Completed, 2009-12	Prostate Cancer丨Neoplasm Metastasis	Bayer	Phase II
**NCT04147819**	Thorium-227	BAY2701439	A First in Human Study of BAY2701439 to Look at Safety, How the Body Absorbs, Distributes and Excretes the Drug, and How Well the Drug Works in Participants With Advanced Cancer Expressing the HER2 Protein	Completed, 2023-09	Cancers With HER2 Expression	Bayer	Phase I
**NCT03724747**	Thorium-227	BAY2315497	Study to Evaluate the Safety, Tolerability,Pharmacokinetics, and Antitumour Activity of a Thorium-227 Labeled Antibody-chelator Conjugate Alone and in Combination With Darolutamide, in Patients With Metastatic Castration Resistant Prostate Cancer	Completed, 2024-10	Metastatic Castration Resistant Prostate Cancer (mCRPC)	Bayer	Phase I
**NCT03507452**	Thorium-227	BAY2287411	First-in-human Study of BAY2287411 Injection, a Thorium-227 Labeled Antibody-chelator Conjugate, in Patients With Tumours Known to Express Mesothelin	Completed, 2022-03	Advanced Recurrent Malignant Pleural Epithelioid Mesothelioma; Advanced Recurrent Malignant Peritoneal Epithelioid Mesothelioma; Advanced Recurrent Serous Ovarian Cancer; Advanced Pancreatic Ductal Adenocarcinoma	Bayer	Phase I
**NCT02581878**	Thorium-227	BAY1862864	Safety and Tolerability of BAY1862864 Injection in Subjects With Relapsed or Refractory CD22-positive Non-Hodgkin's Lymphoma	Completed, 2019-11	Lymphoma, Non-Hodgkin	Bayer	Phase I

Table S5. Key details on the SPECT settings for the image acquisitions, the time points at which the acquisitions are obtained for dosimetry purposes.

**Radioisotope**	**Emission window (KeV) ± half window width (%)**	**Collimator**	**Scatter window (keV) ± half window width (%)**	**Time points (h p.i)**	**Ref.**
Thorium-227	88 keV ± 14%<br>238 keV ±9.5%	ME, HE	**		[86]
Thorium-227	50 keV ± 17.5%<br>82 keV ± 19%<br>239 keV ±9%	ME, HE	36 keV ± 13.8 %<br>62.5 keV ± 5.6 %<br>200 keV ± 5 %	**	[87]
**Radium-223**	82 keV ± 10%	ME	**	0-4, 24,48,96,144	[88]
**Radium-223**	82 keV ± 10%<br>154 keV ± 10%	ME	**	1-5, 18-27, 44-52, 72-120, 168-576	[89]
**Radium-223**	85 keV ±20%<br>154 keV ± 20%<br>270 keV ± 10%	ME, HE	57 ± 17.5%<br>113 ± 8.8%<br>226.5 ± 7.2%	**	[87]
**Actinium-225**	218 keV ± 10%<br>440 keV ± 10%	HE	**		[90]
**Actinium-225**	80 keV ±20%<br>217.5 keV ± 8%<br>410 keV ± 6.1%	ME, HE	57 keV ± 10.7%<br>165 keV ± 21.2%<br>365.5 ± 4.8%	**	[87]
**Actinium-225**	78 keV ± 25%<br>218 keV ± 10%<br>440 keV ± 10%	HE	**	24, 48	[91]
**Bismuth-213**	440 keV ± 10%	HE	**	0.75	[92,93]
**Lead-212**	79 keV ± 20%<br>239 keV ± 10%	ME, HE	55 keV ± 10%<br>103 keV ± 10%<br>209 keV ± 2.5%<br>268 keV ± 2.5%		[94,96]
**Lead-212**	78 keV ± 20%<br>239 keV ± 10%	HE		1.5, 5, 20, 28	[97]
**Astatine-211**	79 keV ± 10%	ME	**	1.5, 5, 11.5,19.5	[98]
Astatine-211	80 keV ± 10 %	ME	61 keV ± 17.5 %		[99]

Acquisition energy windows and scatter windows, window widths, and collimators type (ME=medium-energy, HE=high energy) used to image the main radioisotopes. The time points used for image acquisition in dosimetry protocols are also reported when available in literature. **Not used or reported.

References

1. McDevitt, M.R.; Ma, D.; Simon, J.; Frank, R.K.; Scheinberg, D.A. Design and Synthesis of 225Ac Radioimmunopharmaceuticals. *Appl. Radiat. Isot.* **2002**, *57*, 841–847. https://doi.org/10.1016/S0969-8043(02)00167-7.
2. McDevitt, M.R.; Ma, D.; Simon, J.; Frank, R.K.; Scheinberg, D.A. Design and Synthesis of 225Ac Radioimmunopharmaceuticals. *Appl. Radiat. Isot.* **2002**, *57*, 841–847. https://doi.org/10.1016/S0969-8043(02)00167-7.
3. Deal, K.A.; Davis, I.A.; Mirzadeh, S.; Kennel, S.J.; Brechbiel, M.W. Improved in Vivo Stability of Actinium-225 Macrocyclic Complexes. *J. Med. Chem.* **1999**, *42*, 2988–2992. https://doi.org/10.1021/jm990141f.
4. Chappell, L.L.; Deal, K.A.; Dadachova, E.; Brechbiel, M.W. Synthesis, Conjugation, and Radiolabeling of a Novel Bifunctional Chelating Agent for ^{225}Ac Radioimmunotherapy Applications. *Bioconjug. Chem.* **2000**, *11*, 510–519. https://doi.org/10.1021/bc990153f.
5. Davis, I.A.; Glowienka, K.A.; Boll, R.A.; Deal, K.A.; Brechbiel, M.W.; Stabin, M.; Bochsler, P.N.; Mirzadeh, S.; Kennel, S.J. Comparison of 225actinium Chelates: Tissue Distribution and Radiotoxicity. *Nucl. Med. Biol.* **1999**, *26*, 581–589. https://doi.org/10.1016/s0969-8051(99)00024-4.
6. Holzleitner, N.; Vilangattil, M.; Swaidan, A.; Garcia-Prada, C.D.; Taddio, M.F.; Jeanjean, P.; Mona, C.E.; Lapa, C.; Casini, A.; Günther, T.; et al. Preclinical Evaluation of ^{225}Ac-Labeled Minigastrin Analog DOTA-CCK-66 for Targeted Alpha Therapy. *Eur. J. Nucl. Med. Mol. Imaging* **2025**, *52*, 458–468. https://doi.org/10.1007/s00259-024-06927-z.
7. Li, L.; Rousseau, J.; Jaraquemada-Peláez, M.D.G.; Wang, X.; Robertson, A.; Radchenko, V.; Schaffer, P.; Lin, K.-S.; Bénard, F.; Orvig, C. ^{225}Ac-H_4Py4pa for Targeted Alpha Therapy. *Bioconjug. Chem.* **2021**, *32*, 1348–1363. https://doi.org/10.1021/acs.bioconjchem.0c00171.
8. Yoshida, T.; Jin, K.; Song, H.; Park, S.; Huso, D.L.; Zhang, Z.; Liangfeng, H.; Zhu, C.; Bruchertseifer, F.; Morgenstern, A.; et al. Effective Treatment of Ductal Carcinoma in Situ with a HER-2-Targeted Alpha-Particle Emitting Radionuclide in a Preclinical Model of Human Breast Cancer. *Oncotarget* **2016**, *7*, 33306–33315. https://doi.org/10.18632/oncotarget.8949.
9. Pruszynski, M.; D'Huyvetter, M.; Bruchertseifer, F.; Morgenstern, A.; Lahoutte, T. Evaluation of an Anti-HER2 Nanobody Labeled with ^{225}Ac for Targeted α-Particle Therapy of Cancer. *Mol. Pharm.* **2018**, *15*, 1457–1466. https://doi.org/10.1021/acs.molpharmaceut.7b00985.
10. Puttemans, J.; Dekempeneer, Y.; Eersels, J.L.; Hanssens, H.; Debie, P.; Keyaerts, M.; Windhorst, A.D.; Van Der Aa, F.; Lecocq, Q.; Breckpot, K.; et al. Preclinical Targeted α- and β^--Radionuclide Therapy in HER2-Positive Brain Metastasis Using Camelid Single-Domain Antibodies. *Cancers* **2020**, *12*, 1017. https://doi.org/10.3390/cancers12041017.
11. Pandya, D.N.; Hantgan, R.; Budzevich, M.M.; Kock, N.D.; Morse, D.L.; Batista, I.; Mintz, A.; Li, K.C.; Wadas, T.J. Preliminary Therapy Evaluation of ^{225}Ac-DOTA-c(RGDyK) Demonstrates That Cerenkov Radiation Derived from ^{225}Ac Daughter Decay Can Be Detected by Optical Imaging for In Vivo Tumour Visualization. *Theranostics* **2016**, *6*, 698–709. https://doi.org/10.7150/thno.14338.
12. Sattiraju, A.; Sai, K.K.S.; Xuan, A.; Pandya, D.N.; Almaguel, F.G.; Wadas, T.J.; Herpai, D.M.; Debinski, W.; Mintz, A. IL13RA2 Targeted Alpha Particle Therapy against Glioblastomas. *Oncotarget* **2017**, *8*, 42997–43007. https://doi.org/10.18632/oncotarget.17792.
13. Nedrow, J.R.; Josefsson, A.; Park, S.; Bäck, T.; Hobbs, R.F.; Brayton, C.; Bruchertseifer, F.; Morgenstern, A.; Sgouros, G. Pharmacokinetics, Microscale Distribution, and Dosimetry of Alpha-Emitter-Labeled Anti-PD-L1 Antibodies in an Immune Competent Transgenic Breast Cancer Model. *EJNMMI Res.* **2017**, *7*, 57. https://doi.org/10.1186/s13550-017-0303-2.
14. Thiele, N.A.; Brown, V.; Kelly, J.M.; Amor-Coarasa, A.; Jermilova, U.; MacMillan, S.N.; Nikolopoulou, A.; Ponnala, S.; Ramogida, C.F.; Robertson, A.K.H.; et al. An Eighteen-Membered Macrocyclic Ligand for Actinium-225 Targeted Alpha Therapy. *Angew. Chem. Int. Ed.* **2017**, *56*, 14712–14717. https://doi.org/10.1002/anie.201709532.
15. Kelly, J.M.; Amor-Coarasa, A.; Ponnala, S.; Nikolopoulou, A.; Williams, C.; Thiele, N.A.; Schlyer, D.; Wilson, J.J.; DiMagno, S.G.; Babich, J.W. A Single Dose of ^{225}Ac-RPS-074 Induces a Complete Tumour Response in an LNCaP Xenograft Model. *J. Nucl. Med.* **2019**, *60*, 649–655. https://doi.org/10.2967/jnumed.118.219592.
16. Poty, S.; Membreno, R.; Glaser, J.M.; Ragupathi, A.; Scholz, W.W.; Zeglis, B.M.; Lewis, J.S. The Inverse Electron-Demand Diels–Alder Reaction as a New Methodology for the Synthesis of ^{225}Ac-Labelled Radioimmunoconjugates. *Chem. Commun.* **2018**, *54*, 2599–2602. https://doi.org/10.1039/C7CC09129J.

17. Poty, S.; Carter, L.M.; Mandleywala, K.; Membreno, R.; Abdel-Atti, D.; Ragupathi, A.; Scholz, W.W.; Zeglis, B.M.; Lewis, J.S. Leveraging Bioorthogonal Click Chemistry to Improve 225Ac-Radioimmunotherapy of Pancreatic Ductal Adenocarcinoma. *Clin. Cancer Res.* **2019**, *25*, 868–880. https://doi.org/10.1158/1078-0432.CCR-18-1650.

18. Thorek, D.L.J.; Ku, A.T.; Mitsiades, N.; Veach, D.; Watson, P.A.; Metha, D.; Strand, S.-E.; Sharma, S.K.; Lewis, J.S.; Abou, D.S.; et al. Harnessing Androgen Receptor Pathway Activation for Targeted Alpha Particle Radioimmunotherapy of Breast Cancer. *Clin. Cancer Res.* **2019**, *25*, 881–891. https://doi.org/10.1158/1078-0432.CCR-18-1521.

19. Solomon, V.R.; Alizadeh, E.; Bernhard, W.; Hartimath, S.V.; Hill, W.; Chekol, R.; Barreto, K.M.; Geyer, C.R.; Fonge, H. ^{111}In- and ^{225}Ac-Labeled Cixutumumab for Imaging and α-Particle Radiotherapy of IGF-1R Positive Triple-Negative Breast Cancer. *Mol. Pharm.* **2019**, *16*, 4807–4816. https://doi.org/10.1021/acs.molpharmaceut.9b00542.

20. Ramogida, C.F.; Robertson, A.K.H.; Jermilova, U.; Zhang, C.; Yang, H.; Kunz, P.; Lassen, J.; Bratanovic, I.; Brown, V.; Southcott, L.; et al. Evaluation of Polydentate Picolinic Acid Chelating Ligands and an α-Melanocyte-Stimulating Hormone Derivative for Targeted Alpha Therapy Using ISOL-Produced ^{225}Ac. *EJNMMI Radiopharm. Chem.* **2019**, *4*, 21. https://doi.org/10.1186/s41181-019-0072-5.

21. Tafreshi, N.K.; Tichacek, C.J.; Pandya, D.N.; Doligalski, M.L.; Budzevich, M.M.; Kil, H.; Bhatt, N.B.; Kock, N.D.; Messina, J.L.; Ruiz, E.E.; et al. Melanocortin 1 Receptor–Targeted α-Particle Therapy for Metastatic Uveal Melanoma. *J. Nucl. Med.* **2019**, *60*, 1124–1133. https://doi.org/10.2967/jnumed.118.217240.

22. Tichacek, C.J.; Tafreshi, N.K.; Kil, H.; Engelman, R.W.; Doligalski, M.L.; Budzevich, M.M.; Gage, K.L.; McLaughlin, M.L.; Wadas, T.J.; Silva, A.; et al. Biodistribution and Multicompartment Pharmacokinetic Analysis of a Targeted α Particle Therapy. *Mol. Pharm.* **2020**, *17*, 4180–4188. https://doi.org/10.1021/acs.molpharmaceut.0c00640.

23. Cortez, A.; Josefsson, A.; McCarty, G.; Shtekler, A.E.; Rao, A.; Austin, Z.; Nedrow, J.R. Evaluation of [^{225}Ac]Ac-DOTA-Anti-VLA-4 for Targeted Alpha Therapy of Metastatic Melanoma. *Nucl. Med. Biol.* **2020**, *88–89*, 62–72. https://doi.org/10.1016/j.nucmedbio.2020.07.006.

24. Lakes, A.L.; An, D.D.; Gauny, S.S.; Ansoborlo, C.; Liang, B.H.; Rees, J.A.; McKnight, K.D.; Karsunky, H.; Abergel, R.J. Evaluating ^{225}Ac and ^{177}Lu Radioimmunoconjugates against Antibody–Drug Conjugates for Small-Cell Lung Cancer. *Mol. Pharm.* **2020**, *17*, 4270–4279. https://doi.org/10.1021/acs.molpharmaceut.0c00703.

25. Cheal, S.M.; McDevitt, M.R.; Santich, B.H.; Patel, M.; Yang, G.; Fung, E.K.; Veach, D.R.; Bell, M.; Ahad, A.; Vargas, D.B.; et al. Alpha Radioimmunotherapy Using ^{225}Ac-Proteus-DOTA for Solid Tumours-Safety at Curative Doses. *Theranostics* **2020**, *10*, 11359–11375. https://doi.org/10.7150/thno.48810.

26. Watabe, T.; Liu, Y.; Kaneda-Nakashima, K.; Shirakami, Y.; Lindner, T.; Ooe, K.; Toyoshima, A.; Nagata, K.; Shimosegawa, E.; Haberkorn, U.; et al. Theranostics Targeting Fibroblast Activation Protein in the Tumour Stroma: ^{64}Cu- and ^{225}Ac-Labeled FAPI-04 in Pancreatic Cancer Xenograft Mouse Models. *J. Nucl. Med.* **2020**, *61*, 563–569. https://doi.org/10.2967/jnumed.119.233122.

27. Qin, Y.; Imobersteg, S.; Blanc, A.; Frank, S.; Schibli, R.; Béhé, M.P.; Grzmil, M. Evaluation of Actinium-225 Labeled Minigastrin Analogue [^{225}Ac]Ac-DOTA-PP-F11N for Targeted Alpha Particle Therapy. *Pharmaceutics* **2020**, *12*, 1088. https://doi.org/10.3390/pharmaceutics12111088.

28. Bell, M.M.; Gutsche, N.T.; King, A.P.; Baidoo, K.E.; Kelada, O.J.; Choyke, P.L.; Escorcia, F.E. Glypican-3-Targeted Alpha Particle Therapy for Hepatocellular Carcinoma. *Molecules* **2020**, *26*, 4. https://doi.org/10.3390/molecules26010004.

29. Fichou, N.; Gouard, S.; Maurel, C.; Barbet, J.; Ferrer, L.; Morgenstern, A.; Bruchertseifer, F.; Faivre-Chauvet, A.; Bigot-Corbel, E.; Davodeau, F.; et al. Single-Dose Anti-CD138 Radioimmunotherapy: Bismuth-213 Is More Efficient than Lutetium-177 for Treatment of Multiple Myeloma in a Preclinical Model. *Front. Med.* **2015**, *2*, 76. https://doi.org/10.3389/fmed.2015.00076.

30. Teiluf, K.; Seidl, C.; Blechert, B.; Gaertner, F.C.; Gilbertz, K.-P.; Fernandez, V.; Bassermann, F.; Endell, J.; Boxhammer, R.; Leclair, S.; et al. α-Radioimmunotherapy with ^{213}Bi-Anti-CD38 Immunoconjugates Is Effective in a Mouse Model of Human Multiple Myeloma. *Oncotarget* **2015**, *6*, 4692–4703. https://doi.org/10.18632/oncotarget.2986.

31. Fazel, J.; Rötzer, S.; Seidl, C.; Feuerecker, B.; Autenrieth, M.; Weirich, G.; Bruchertseifer, F.; Morgenstern, A.; Senekowitsch-Schmidtke, R. Fractionated Intravesical Radioimmunotherapy with ^{213}Bi-Anti-EGFR-MAb Is Effective without Toxic Side-Effects in a Nude Mouse Model of Advanced Human Bladder Carcinoma. *Cancer Biol. Ther.* **2015**, *16*, 1526–1534. https://doi.org/10.1080/15384047.2015.1071735.

32. Kunikowska, J.; Królicki, L. Targeted α-Emitter Therapy of Neuroendocrine Tumours. *Semin. Nucl. Med.* **2020**, *50*, 171–176. https://doi.org/10.1053/j.semnuclmed.2019.11.003.

33. Chan, H.S.; Konijnenberg, M.W.; de Blois, E.; Koelewijn, S.; Baum, R.P.; Morgenstern, A.; Bruchertseifer, F.; Breeman, W.A.; de Jong, M. Influence of Tumour Size on the Efficacy of Targeted Alpha Therapy with ^{213}Bi-[DOTA^{0},Tyr^{3}]-Octreotate. *EJNMMI Res.* **2016**, *6*, 6. https://doi.org/10.1186/s13550-016-0162-2.

34. Eriksson, S.E.; Bäck, T.; Elgström, E.; Jensen, H.; Nilsson, R.; Lindegren, S.; Tennvall, J. Successful Radioimmunotherapy of Established Syngeneic Rat Colon Carcinoma with ^{211}At-mAb. *EJNMMI Res.* **2013**, *3*, 23. https://doi.org/10.1186/2191-219X-3-23.
35. Eriksson, S.E.; Elgström, E.; Bäck, T.; Ohlsson, T.; Jensen, H.; Nilsson, R.; Lindegren, S.; Tennvall, J. Sequential Radioimmunotherapy with ^{177}Lu- and ^{211}At-Labeled Monoclonal Antibody BR96 in a Syngeneic Rat Colon Carcinoma Model. *Cancer Biother. Radiopharm.* **2014**, *29*, 238–246. https://doi.org/10.1089/cbr.2014.1625.
36. Liu, W.; Ma, H.; Liang, R.; Chen, X.; Li, H.; Lan, T.; Yang, J.; Liao, J.; Qin, Z.; Yang, Y.; et al. Targeted Alpha Therapy of Glioma Using ^{211}At-Labeled Heterodimeric Peptide Targeting Both VEGFR and Integrins. *Mol. Pharm.* **2022**, *19*, 3206–3216. https://doi.org/10.1021/acs.molpharmaceut.2c00349.
37. Lyczko, M.; Pruszynski, M.; Majkowska-Pilip, A.; Lyczko, K.; Was, B.; Meczynska-Wielgosz, S.; Kruszewski, M.; Szkliniarz, K.; Jastrzebski, J.; Stolarz, A.; et al. 211At Labeled Substance P (5-11) as Potential Radiopharmaceutical for Glioma Treatment. *Nucl. Med. Biol.* **2017**, *53*, 1–8. https://doi.org/10.1016/j.nucmedbio.2017.05.008.
38. Meyer, G.J.; Walte, A.; Sriyapureddy, S.R.; Grote, M.; Krull, D.; Korkmaz, Z.; Knapp, W.H. Synthesis and Analysis of 2-[^{211}At]-L-Phenylalanine and 4-[^{211}At]-L-Phenylalanine and Their Uptake in Human Glioma Cell Cultures in-Vitro. *Appl. Radiat. Isot. Data Instrum. Methods Use Agric. Ind. Med.* **2010**, *68*, 1060–1065. https://doi.org/10.1016/j.apradiso.2009.12.043.
39. Borrmann, N.; Friedrich, S.; Schwabe, K.; Hedrich, H.J.; Krauss, J.K.; Knapp, W.H.; Nakamura, M.; Meyer, G.-J.; Walte, A. Systemic Treatment with 4-^{211}Atphenylalanine Enhances Survival of Rats with Intracranial Glioblastoma. *Nukl. Nucl. Med.* **2013**, *52*, 212–221. https://doi.org/10.3413/Nukmed-0580-13-05.
40. Zalutsky, M.R.; Stabin, M.G.; Larsen, R.H.; Bigner, D.D. Tissue Distribution and Radiation Dosimetry of Astatine-211-Labeled Chimeric 81C6, an Alpha-Particle-Emitting Immunoconjugate. *Nucl. Med. Biol.* **1997**, *24*, 255–261. https://doi.org/10.1016/s0969-8051(97)00060-7.
41. Cheng, J.; Ekberg, T.; Engström, M.; Nestor, M.; Jensen, H.J.; Tolmachev, V.; Anniko, M. Radioimmunotherapy With Astatine-211 Using Chimeric Monoclonal Antibody U36 in Head and Neck Squamous Cell Carcinoma. *Laryngoscope* **2007**, *117*, 1013–1018. https://doi.org/10.1097/MLG.0b013e31804b1a6d.
42. Walte, A.; Sriyapureddy, S.S.R.; Korkmaz, Z.; Krull, D.; Bolte, O.; Hofmann, M.; Meyer, G.-J.; Knapp, W.H. Preparation and Evaluation of 211At Labelled Antineoplastic Antibodies. *J. Pharm. Pharm. Sci. Publ. Can. Soc. Pharm. Sci. Soc. Can. Sci. Pharm.* **2007**, *10*, 277–285.
43. Orozco, J.J.; Bäck, T.; Kenoyer, A.; Balkin, E.R.; Hamlin, D.K.; Wilbur, D.S.; Fisher, D.R.; Frayo, S.L.; Hylarides, M.D.; Green, D.J.; et al. Anti-CD45 Radioimmunotherapy Using ^{211}At with Bone Marrow Transplantation Prolongs Survival in a Disseminated Murine Leukemia Model. *Blood* **2013**, *121*, 3759–3767. https://doi.org/10.1182/blood-2012-11-467035.
44. Nakamae, H.; Wilbur, D.S.; Hamlin, D.K.; Thakar, M.S.; Santos, E.B.; Fisher, D.R.; Kenoyer, A.L.; Pagel, J.M.; Press, O.W.; Storb, R.; et al. Biodistributions, Myelosuppression, and Toxicities in Mice Treated with an Anti-CD45 Antibody Labeled with the Alpha-Emitting Radionuclides Bismuth-213 or Astatine-211. *Cancer Res.* **2009**, *69*, 2408–2415. https://doi.org/10.1158/0008-5472.CAN-08-4363.
45. Oriuchi, N.; Aoki, M.; Ukon, N.; Washiyama, K.; Tan, C.; Shimoyama, S.; Nishijima, K.; Takahashi, K.; Ito, H.; Ikezoe, T.; et al. Possibility of Cancer-Stem-Cell-Targeted Radioimmunotherapy for Acute Myelogenous Leukemia Using 211At-CXCR4 Monoclonal Antibody. *Sci. Rep.* **2020**, *10*, 6810. https://doi.org/10.1038/s41598-020-63557-9.
46. Laszlo, G.S.; Orozco, J.J.; Kehret, A.R.; Lunn, M.C.; Huo, J.; Hamlin, D.K.; Scott Wilbur, D.; Dexter, S.L.; Comstock, M.L.; O'Steen, S.; et al. Development of [^{211}At]Astatine-Based Anti-CD123 Radioimmunotherapy for Acute Leukemias and Other CD123+ Malignancies. *Leukemia* **2022**, *36*, 1485–1491. https://doi.org/10.1038/s41375-022-01580-7.
47. Green, D.J.; Shadman, M.; Jones, J.C.; Frayo, S.L.; Kenoyer, A.L.; Hylarides, M.D.; Hamlin, D.K.; Wilbur, D.S.; Balkan, E.R.; Lin, Y.; et al. Astatine-211 Conjugated to an Anti-CD20 Monoclonal Antibody Eradicates Disseminated B-Cell Lymphoma in a Mouse Model. *Blood* **2015**, *125*, 2111–2119. https://doi.org/10.1182/blood-2014-11-612770.
48. Zhao, B.; Qin, S.; Chai, L.; Lu, G.; Yang, Y.; Cai, H.; Yuan, X.; Fan, S.; Huang, Q.; Yu, F. Evaluation of Astatine-211-Labeled Octreotide as a Potential Radiotherapeutic Agent for NSCLC Treatment. *Bioorg. Med. Chem.* **2018**, *26*, 1086–1091. https://doi.org/10.1016/j.bmc.2018.01.023.
49. Link, E.M.; Costa, D.C.; Lui, D.; Ell, P.J.; Blower, P.J.; Spittle, M.F. Targeting Disseminated Melanoma with Radiolabelled Methylene Blue: Comparative Bio-Distribution Studies in Man and Animals. *Acta Oncol.* **1996**, *35*, 331–341. https://doi.org/10.3109/02841869609101650.
50. O'Steen, S.; Comstock, M.L.; Orozco, J.J.; Hamlin, D.K.; Wilbur, D.S.; Jones, J.C.; Kenoyer, A.; Nartea, M.E.; Lin, Y.; Miller, B.W.; et al. The α-Emitter Astatine-211 Targeted to CD38 Can Eradicate Multiple Myeloma in a Disseminated Disease Model. *Blood* **2019**, *134*, 1247–1256. https://doi.org/10.1182/blood.2019001250.

51. Gouard, S.; Maurel, C.; Marionneau-Lambot, S.; Dansette, D.; Bailly, C.; Guérard, F.; Chouin, N.; Haddad, F.; Alliot, C.; Gaschet, J.; et al. Targeted-Alpha-Therapy Combining Astatine-211 and Anti-CD138 Antibody in A Preclinical Syngeneic Mouse Model of Multiple Myeloma Minimal Residual Disease. *Cancers* **2020**, *12*, 2721. https://doi.org/10.3390/cancers12092721.
52. Makvandi, M.; Dupis, E.; Engle, J.W.; Nortier, F.M.; Fassbender, M.E.; Simon, S.; Birnbaum, E.R.; Atcher, R.W.; John, K.D.; Rixe, O.; et al. Alpha-Emitters and Targeted Alpha Therapy in Oncology: From Basic Science to Clinical Investigations. *Target. Oncol.* **2018**, *13*, 189–203. https://doi.org/10.1007/s11523-018-0550-9.
53. Vaidyanathan, G.; Friedman, H.S.; Keir, S.T.; Zalutsky, M.R. Evaluation of Meta-[^{211}At]Astatobenzylguanidine in an Athymic Mouse Human Neuroblastoma Xenograft Model. *Nucl. Med. Biol.* **1996**, *23*, 851–856. https://doi.org/10.1016/0969-8051(96)00115-1.
54. Sudo, H.; Tsuji, A.B.; Sugyo, A.; Nagatsu, K.; Minegishi, K.; Ishioka, N.S.; Ito, H.; Yoshinaga, K.; Higashi, T. Preclinical Evaluation of the Acute Radiotoxicity of the α-Emitting Molecular-Targeted Therapeutic Agent 211At-MABG for the Treatment of Malignant Pheochromocytoma in Normal Mice. *Transl. Oncol.* **2019**, *12*, 879–888. https://doi.org/10.1016/j.tranon.2019.04.008.
55. Palm, S.; Bäck, T.; Aneheim, E.; Hallqvist, A.; Hultborn, R.; Jacobsson, L.; Jensen, H.; Lindegren, S.; Albertsson, P. Evaluation of Therapeutic Efficacy of ^{211}At-Labeled Farletuzumab in an Intraperitoneal Mouse Model of Disseminated Ovarian Cancer. *Transl. Oncol.* **2021**, *14*, 100873. https://doi.org/10.1016/j.tranon.2020.100873.
56. Elgqvist, J.; Andersson, H.; Bäck, T.; Claesson, I.; Hultborn, R.; Jensen, H.; Lindegren, S.; Olsson, M.; Palm, S.; Warnhammar, E.; et al. Fractionated Radioimmunotherapy of Intraperitoneally Growing Ovarian Cancer in Nude Mice with ^{211}At-MX35 F(Ab′)2: Therapeutic Efficacy and Myelotoxicity. *Nucl. Med. Biol.* **2006**, *33*, 1065–1072. https://doi.org/10.1016/j.nucmedbio.2006.07.009.
57. Gustafsson, A.M.E.; Bäck, T.; Elgqvist, J.; Jacobsson, L.; Hultborn, R.; Albertsson, P.; Morgenstern, A.; Bruchertseifer, F.; Jensen, H.; Lindegren, S. Comparison of Therapeutic Efficacy and Biodistribution of ^{213}Bi- and ^{211}At-Labeled Monoclonal Antibody MX35 in an Ovarian Cancer Model. *Nucl. Med. Biol.* **2012**, *39*, 15–22. https://doi.org/10.1016/j.nucmedbio.2011.07.003.
58. Palm, S.; Bäck, T.; Claesson, I.; Danielsson, A.; Elgqvist, J.; Frost, S.; Hultborn, R.; Jensen, H.; Lindegren, S.; Jacobsson, L. Therapeutic Efficacy of Astatine-211–Labeled Trastuzumab on Radioresistant SKOV-3 Tumours in Nude Mice. *Int. J. Radiat. Oncol.* **2007**, *69*, 572–579. https://doi.org/10.1016/j.ijrobp.2007.06.023.
59. Li, H.K.; Morokoshi, Y.; Nagatsu, K.; Kamada, T.; Hasegawa, S. Locoregional Therapy with α-Emitting Trastuzumab against Peritoneal Metastasis of Human Epidermal Growth Factor Receptor 2-Positive Gastric Cancer in Mice. *Cancer Sci.* **2017**, *108*, 1648–1656. https://doi.org/10.1111/cas.13282.
60. Dekempeneer, Y.; Bäck, T.; Aneheim, E.; Jensen, H.; Puttemans, J.; Xavier, C.; Keyaerts, M.; Palm, S.; Albertsson, P.; Lahoutte, T.; et al. Labeling of Anti-HER2 Nanobodies with Astatine-211: Optimization and the Effect of Different Coupling Reagents on Their in Vivo Behavior. *Mol. Pharm.* **2019**, *16*, 3524–3533. https://doi.org/10.1021/acs.molpharmaceut.9b00354.
61. Feng, Y.; Meshaw, R.; Zhao, X.-G.; Jannetti, S.; Vaidyanathan, G.; Zalutsky, M.R. Effective Treatment of Human Breast Carcinoma Xenografts with Single-Dose ^{211}At-Labeled Anti-HER2 Single-Domain Antibody Fragment. *J. Nucl. Med. Off. Publ. Soc. Nucl. Med.* **2023**, *64*, 124–130. https://doi.org/10.2967/jnumed.122.264071.
62. Robinson, M.K.; Shaller, C.; Garmestani, K.; Plascjak, P.S.; Hodge, K.M.; Yuan, Q.-A.; Marks, J.D.; Waldmann, T.A.; Brechbiel, M.W.; Adams, G.P. Effective Treatment of Established Human Breast Tumour Xenografts in Immunodeficient Mice with a Single Dose of the Alpha-Emitting Radioisotope Astatine-211 Conjugated to Anti-HER2/Neu Diabodies. *Clin. Cancer Res. Off. J. Am. Assoc. Cancer Res.* **2008**, *14*, 875–882. https://doi.org/10.1158/1078-0432.CCR-07-1250.
63. Kiess, A.P.; Minn, I.; Vaidyanathan, G.; Hobbs, R.F.; Josefsson, A.; Shen, C.; Brummet, M.; Chen, Y.; Choi, J.; Koumarianou, E.; et al. (2 *S*)-2-(3-(1-Carboxy-5-(4-^{211}At-Astatobenzamido)Pentyl)Ureido)-Pentanedioic Acid for PSMA-Targeted α-Particle Radiopharmaceutical Therapy. *J. Nucl. Med.* **2016**, *57*, 1569–1575. https://doi.org/10.2967/jnumed.116.174300.
64. Watabe, T.; Kaneda-Nakashima, K.; Shirakami, Y.; Liu, Y.; Ooe, K.; Teramoto, T.; Toyoshima, A.; Shimosegawa, E.; Nakano, T.; Kanai, Y.; et al. Targeted Alpha Therapy Using Astatine (^{211}At)-Labeled Phenylalanine: A Preclinical Study in Glioma Bearing Mice. *Oncotarget* **2020**, *11*, 1388–1398. https://doi.org/10.18632/oncotarget.27552.
65. Bäck, T.A.; Jennbacken, K.; Hagberg Thulin, M.; Lindegren, S.; Jensen, H.; Olafsen, T.; Yazaki, P.J.; Palm, S.; Albertsson, P.; Damber, J.-E.; et al. Targeted Alpha Therapy with Astatine-211-Labeled Anti-PSCA A11 Minibody Shows Antitumour Efficacy in Prostate Cancer Xenografts and Bone Microtumours. *EJNMMI Res.* **2020**, *10*, 10. https://doi.org/10.1186/s13550-020-0600-z.
66. Aoki, M.; Zhao, S.; Takahashi, K.; Washiyama, K.; Ukon, N.; Tan, C.; Shimoyama, S.; Nishijima, K.-I.; Ogawa, K. Preliminary Evaluation of Astatine-211-Labeled Bombesin Derivatives for Targeted Alpha Therapy. *Chem. Pharm. Bull.* **2020**, *68*, 538–545. https://doi.org/10.1248/cpb.c20-00077.
67. Carlin, S.; Mairs, R.J.; Welsh, P.; Zalutsky, M.R. Sodium-Iodide Symporter (NIS)-Mediated Accumulation of [^{211}At]Astatide in NIS-Transfected Human Cancer Cells. *Nucl. Med. Biol.* **2002**, *29*, 729–739. https://doi.org/10.1016/s0969-8051(02)00332-3.

68. Petrich, T.; Helmeke, H.-J.; Meyer, G.J.; Knapp, W.H.; Pötter, E. Establishment of Radioactive Astatine and Iodine Uptake in Cancer Cell Lines Expressing the Human Sodium/Iodide Symporter. *Eur. J. Nucl. Med. Mol. Imaging* **2002**, *29*, 842–854. https://doi.org/10.1007/s00259-002-0784-7.
69. Petrich, T.; Quintanilla-Fend, L.; Knapp, W.; Pötter, E. Effective Cancer Therapy by the α-Particle Emitter [Astatine-211]-Astatine in a Mouse Model of Genetically Modified NIS-Expressing Tumours. *Exp. Clin. Endocrinol. Diabetes* **2005**, *113*, s-2005-862947. https://doi.org/10.1055/s-2005-862947.
70. Sporer, E.; Poulie, C.B.M.; Lindegren, S.; Aneheim, E.; Jensen, H.; Bäck, T.; Kempen, P.J.; Kjaer, A.; Herth, M.M.; Jensen, A.I. Surface Adsorption of the Alpha-Emitter Astatine-211 to Gold Nanoparticles Is Stable In Vivo and Potentially Useful in Radionuclide Therapy. *J. Nanotheranostics* **2021**, *2*, 196–207. https://doi.org/10.3390/jnt2040012.
71. Aso, A.; Nabetani, H.; Matsuura, Y.; Kadonaga, Y.; Shirakami, Y.; Watabe, T.; Yoshiya, T.; Mochizuki, M.; Ooe, K.; Kawakami, A.; et al. Evaluation of Astatine-211-Labeled Fibroblast Activation Protein Inhibitor (FAPI): Comparison of Different Linkers with Polyethylene Glycol and Piperazine. *Int. J. Mol. Sci.* **2023**, *24*, 8701. https://doi.org/10.3390/ijms24108701.
72. Li, M.; Baumhover, N.J.; Liu, D.; Cagle, B.S.; Boschetti, F.; Paulin, G.; Lee, D.; Dai, Z.; Obot, E.R.; Marks, B.M.; et al. Preclinical Evaluation of a Lead Specific Chelator (PSC) Conjugated to Radiopeptides for 203Pb and 212Pb-Based Theranostics. *Pharmaceutics* **2023**, *15*, 414. https://doi.org/10.3390/pharmaceutics15020414.
73. Horak, E.; Hartmann, F.; Garmestani, K.; Wu, C.; Brechbiel, M.; Gansow, O.A.; Landolfi, N.F.; Waldmann, T.A. Radioimmunotherapy Targeting of HER2/Neu Oncoprotein on Ovarian Tumour Using Lead-212-DOTA-AE1. *J. Nucl. Med. Off. Publ. Soc. Nucl. Med.* **1997**, *38*, 1944–1950.
74. Banerjee, S.R.; Minn, I.; Kumar, V.; Josefsson, A.; Lisok, A.; Brummet, M.; Chen, J.; Kiess, A.P.; Baidoo, K.; Brayton, C.; et al. Preclinical Evaluation of $^{203/212}$Pb-Labeled Low-Molecular-Weight Compounds for Targeted Radiopharmaceutical Therapy of Prostate Cancer. *J. Nucl. Med.* **2020**, *61*, 80–88. https://doi.org/10.2967/jnumed.119.229393.
75. Stenberg, V.Y.; Juzeniene, A.; Chen, Q.; Yang, X.; Bruland, Ø.S.; Larsen, R.H. Preparation of the Alpha-emitting Prostate-specific Membrane Antigen Targeted Radioligand [^{212}Pb]Pb-NG001 for Prostate Cancer. *J. Label. Compd. Radiopharm.* **2020**, *63*, 129–143. https://doi.org/10.1002/jlcr.3825.
76. Rold, T.L.; Devanny, E.A.; Okoye, N.C.; Quinn, T.P.; Hoffman, T.J. Abstract 5347: Preliminary Evaluation of BB2r TAT Using 212Pb-RM2 in a PC3 Human Prostate Cancer Xenograft Model. *Cancer Res.* **2020**, *80*, 5347. https://doi.org/10.1158/1538-7445.AM2020-5347.
77. Beyer, G.-J.; Miederer, M.; Vranješ-Đurić, S.; Čomor, J.J.; Künzi, G.; Hartley, O.; Senekowitsch-Schmidtke, R.; Soloviev, D.; Buchegger, F.; and the ISOLDE Collaboration Targeted Alpha Therapy in Vivo: Direct Evidence for Single Cancer Cell Kill Using 149Tb-Rituximab. *Eur. J. Nucl. Med. Mol. Imaging* **2004**, *31*, 547–554. https://doi.org/10.1007/s00259-003-1413-9.
78. Müller, C.; Reber, J.; Haller, S.; Dorrer, H.; Köster, U.; Johnston, K.; Zhernosekov, K.; Türler, A.; Schibli, R. Folate Receptor Targeted Alpha-Therapy Using Terbium-149. *Pharmaceuticals* **2014**, *7*, 353–365. https://doi.org/10.3390/ph7030353.
79. Henriksen, G.; Fisher, D.R.; Roeske, J.C.; Bruland, Ø.S.; Larsen, R.H. Targeting of Osseous Sites with Alpha-Emitting 223Ra: Comparison with the Beta-Emitter 89Sr in Mice. *J. Nucl. Med. Off. Publ. Soc. Nucl. Med.* **2003**, *44*, 252–259.
80. Larsen, R.H.; Saxtorph, H.; Skydsgaard, M.; Borrebaek, J.; Jonasdottir, T.J.; Bruland, O.S.; Klastrup, S.; Harling, R.; Ramdahl, T. Radiotoxicity of the Alpha-Emitting Bone-Seeker 223Ra Injected Intravenously into Mice: Histology, Clinical Chemistry and Hematology. *Vivo Athens Greece* **2006**, *20*, 325–331.
81. Suominen, M.I.; Fagerlund, K.M.; Rissanen, J.P.; Konkol, Y.M.; Morko, J.P.; Peng, Z.; Alhoniemi, E.J.; Laine, S.K.; Corey, E.; Mumberg, D.; et al. Radium-223 Inhibits Osseous Prostate Cancer Growth by Dual Targeting of Cancer Cells and Bone Microenvironment in Mouse Models. *Clin. Cancer Res. Off. J. Am. Assoc. Cancer Res.* **2017**, *23*, 4335–4346. https://doi.org/10.1158/1078-0432.CCR-16-2955.
82. Dahle, J.; Jonasdottir, T.J.; Heyerdahl, H.; Nesland, J.M.; Borrebaek, J.; Hjelmerud, A.K.; Larsen, R.H. Assessment of Long-Term Radiotoxicity after Treatment with the Low-Dose-Rate Alpha-Particle-Emitting Radioimmunoconjugate ^{227}Th-Rituximab. *Eur. J. Nucl. Med. Mol. Imaging* **2010**, *37*, 93–102. https://doi.org/10.1007/s00259-009-1197-7.
83. Heyerdahl, H.; Abbas, N.; Brevik, E.M.; Mollatt, C.; Dahle, J. Fractionated Therapy of HER2-Expressing Breast and Ovarian Cancer Xenografts in Mice with Targeted Alpha Emitting 227Th-DOTA-p-Benzyl-Trastuzumab. *PLoS ONE* **2012**, *7*, e42345. https://doi.org/10.1371/journal.pone.0042345.
84. Hagemann, U.B.; Wickstroem, K.; Wang, E.; Shea, A.O.; Sponheim, K.; Karlsson, J.; Bjerke, R.M.; Ryan, O.B.; Cuthbertson, A.S. In Vitro and In Vivo Efficacy of a Novel CD33-Targeted Thorium-227 Conjugate for the Treatment of Acute Myeloid Leukemia. *Mol. Cancer Ther.* **2016**, *15*, 2422–2431. https://doi.org/10.1158/1535-7163.MCT-16-0251.

85. Wickstroem, K.; Hagemann, U.B.; Kristian, A.; Ellingsen, C.; Sommer, A.; Ellinger-Ziegelbauer, H.; Wirnitzer, U.; Hagelin, E.-M.; Larsen, A.; Smeets, R.; et al. Preclinical Combination Studies of an FGFR2 Targeted Thorium-227 Conjugate and the ATR Inhibitor BAY 1895344. *Int. J. Radiat. Oncol. Biol. Phys.* **2019**, *105*, 410–422. https://doi.org/10.1016/j.ijrobp.2019.06.2508.
86. Murray, I.; Rojas, B.; Gear, J.; Callister, R.; Cleton, A.; Flux, G.D. Quantitative Dual-Isotope Planar Imaging of Thorium-227 and Radium-223 Using Defined Energy Windows. *Cancer Biother. Radiopharm.* **2020**, *35*, 530–539.
87. Benabdallah, N.; Scheve, W.; Dunn, N.; Silvestros, D.; Schelker, P.; Abou, D.; Jammalamadaka, U.; Laforest, R.; Li, Z.; Liu, J.; et al. Practical considerations for quantitative clinical SPECT/CT imaging of alpha particle emitting radioisotopes. *Theranostics* **2021**, *11*, 9721–9737.
88. Chittenden, S.J.; Hindorf, C.; Parker, C.C.; Lewington, V.J.; Pratt, B.E.; Johnson, B.; Flux, G.D. A Phase 1, Open-Label Study of the Biodistribution, Pharmacokinetics, and Dosimetry of ^{223}Ra-Dichloride in Patients with Hormone-Refractory Prostate Cancer and Skeletal Metastases. *J. Nucl. Med.* **2015**, *56*, 1304–1309.
89. Pacilio, M.; Ventroni, G.; De Vincentis, G.; Cassano, B.; Pellegrini, R.; Di Castro, E.; Frantellizzi, V.; Follacchio, G.A.; Garkavaya, T.; Lorenzon, L.; et al. Dosimetry of bone metastases in targeted radionuclide therapy with alpha-emitting ^{223}Ra-dichloride. *Eur. J. Nucl. Med. Mol. Imaging* **2016**, *43*, 21–33.
90. Ocak, M.; Toklu, T.; Demirci, E.; Selçuk, N.; Kabasakal, L. Post-therapy imaging of 225Ac-DOTATATE treatment in a patient with recurrent neuroendocrine tumor. *Eur. J. Nucl. Med. Mol. Imaging* **2020**, *47*, 2711–2712.
91. Liubchenko, G.; Böning, G.; Zacherl, M.; Rumiantcev, M.; Unterrainer, L.M.; Gildehaus, F.J.; Brendel, M.; Resch, S.; Bartenstein, P.; Ziegler, S.I.; et al. Image-based dosimetry for [^{225}Ac]Ac-PSMA-I&T therapy and the effect of daughter-specific pharmacokinetics. *Eur. J. Nucl. Med. Mol. Imaging* **2024**, *51*, 2504–2514.
92. Sgouros, G.; Ballangrud, A.M.; Jurcic, J.G.; McDevitt, M.R.; Humm, J.L.; Erdi, Y.E.; Mehta, B.M.; Finn, R.D.; Larson, S.M.; Scheinberg, D.A. Pharmacokinetics and dosimetry of an alpha-particle emitter labeled antibody: ^{213}Bi-HuM195 (anti-CD33) in patients with leukemia. *J. Nucl. Med.* **1999**, *40*, 1935–1946.
93. Kratochwil, C.; Giesel, F.L.; Bruchertseifer, F.; Mier, W.; Apostolidis, C.; Boll, R.; Murphy, K.; Haberkorn, U.; Morgenstern, A. ^{213}Bi-DOTATOC receptor-targeted alpha-radionuclide therapy induces remission in neuroendocrine tumours refractory to beta radiation: A first-in-human experience. *Eur. J. Nucl. Med. Mol. Imaging* **2014**, *41*, 2106–2119.
94. Kvassheim, M.; Revheim, M.E.R.; Stokke, C. Quantitative SPECT/CT imaging of lead-212: A phantom study. *EJNMMI Phys.* **2022**, *9*, 52.
95. Kästner, D.; Hartmann, H.; Freudenberg, R.; Pretze, M.; Brogsitter, C.; Schultz, M.K.; Kotzerke, J.; Michler, E. Gamma camera imaging characteristics of $^{203/212}$Pb as a theragnostic pair for targeted alpha therapy: A feasibility study. *EJNMMI Phys.* **2025**, *12*, 50. https://doi.org/10.1186/s40658-025-00763-2.
96. Ramonaheng, K.; Qebetu, M.; Banda, K.; Goorhoo, P.; Legodi, K.; Mdanda, S.; Sibiya, S.; Mzizi, Y.; Ndlovu, H.; Kabunda, J.; et al. Advances in Dosimetry and Imaging for ^{203}Pb and ^{212}Pb Radiotheranostics. *Semin. Nucl. Med.* 2025, *in press*.
97. Griffiths, M.R.; Pattison, D.A.; Latter, M.; Kuan, K.; Taylor, S.; Tieu, W.; Kryza, T.; Meyrick, D.; Lee, B.Q.; Hansen, A.; et al. First-in-Human ^{212}Pb-PSMA–Targeted a-Therapy SPECT/CT Imaging in a Patient with Metastatic Castration-Resistant Prostate Cancer. *J. Nucl. Med.* **2024**, *65*, 664.
98. Andersson, H.; Cederkrantz, E.; Bäck, T.; Divgi, C.; Elgqvist, J.; Himmelman, J.; Horvath, G.; Jacobsson, L.; Jensen, H.; Lindegren, S.; et al. Intraperitoneal alpha-particle radioimmunotherapy of ovarian cancer patients: Pharmacokinetics and dosimetry of ^{211}At-$MX_{35}F(ab')_2$—A phase I study. *J. Nucl. Med.* **2009**, *50*, 1153–1160.
99. Turkington, T.G.; Zalutsky, M.R.; Jaszczak, R.J.; Garg, P.K.; Vaidyanathan, G.; Coleman, R.E. Measuring astatine-211 distributions with SPECT. *Phys. Med. Biol.* **1993**, *38*, 1121–1130.
